# Individual and combined effects of *GSTM1*, *GSTT1*, and *GSTP1* polymorphisms on lung cancer risk: A meta-analysis and re-analysis of systematic meta-analyses

**DOI:** 10.1097/MD.0000000000026104

**Published:** 2021-07-02

**Authors:** Wen-Ping Zhang, Chen Yang, Ling-Jun Xu, Wei Wang, Liang Song, Xiao-Feng He

**Affiliations:** a Department of Cardiothoracic Surgery, Heping Hospital Affiliated to Changzhi Medical College; b Teaching Reform Class of 2016 of the First Clinical College, Changzhi Medical College, Shanxi, Changzhi City; c Department of Pain Management, the First Affiliated Hospital, Jinan University, Guangzhou City; d Beijing Zhendong Guangming Pharmaceutical Research Institute Co Ltd, Beijing City; e Endoscopy Room; f Department of Science and Education, Heping Hospital Affiliated to Changzhi Medical College, Shanxi, Changzhi City, PR China.

**Keywords:** BFDP, FPRP, GSTM1, GSTP1, GSTT1, lung cancer

## Abstract

Thirty-five previous meta-analyses have been reported on the individual glutathione S-transferase M1 (*GSTM1*) present/null, glutathione S-transferase T1 (*GSTT1*) present/null, and glutathione S-transferase P1 (*GSTP1*) IIe105Val polymorphisms with lung cancer (LC) risk. However, they did not appraise the credibility and explore the combined effects between the 3 genes and LC risk.

We performed a meta-analysis and re-analysis of systematic previous meta-analyses to solve the above problems.

Meta-analyses of Observational Studies in Epidemiology guidelines were used. Moreover, we employed false-positive report probability (FPRP), Bayesian false discovery probability (BFDP), and the Venice criteria to verify the credibility of current and previous meta-analyses.

Significantly increased LC risk was considered as “highly credible” or “positive” for *GSTM1* null genotype in Japanese (odds ratio (OR) = 1.30, 95% confidence interval (CI) = 1.17–1.44, *I*^2^ = 0.0%, statistical power = 0.997, FPRP = 0.008, BFDP = 0.037, and Venice criteria: AAB), for *GSTT1* null genotype in Asians (OR = 1.23, 95% CI = 1.12–1.36, *I*^2^ = 49.1%, statistical power = 1.000, FPRP = 0.051, BFDP = 0.771, and Venice criteria: ABB), especially Chinese populations (OR = 1.31, 95% CI = 1.16–1.49, *I*^2^ = 48.9%, Statistical power = 0.980, FPRP = 0.039, BFDP = 0.673, and Venice criteria: ABB), and for *GSTP1* IIe105Val polymorphism in Asians (Val vs IIe: OR = 1.28, 95% CI = 1.17–1.42, *I*^2^ = 30.3%, statistical power = 0.999, FPRP = 0.003, BFDP = 0.183, and Venice criteria: ABB). Significantly increased lung adenocarcinoma (AC) risk was also considered as “highly credible” or “positive” in Asians for the *GSTM1* (OR = 1.35, 95% CI = 1.22–1.48, *I*^2^ = 25.5%, statistical power = 0.988, FPRP < 0.001, BFDP < 0.001, and Venice criteria: ABB) and *GSTT1* (OR = 1.36, 95% CI = 1.17–1.58, *I*^2^ = 30.2%, statistical power = 0.900, FPRP = 0.061, BFDP = 0.727, and Venice criteria: ABB) null genotype.

This study indicates that *GSTM1* null genotype is associated with increased LC risk in Japanese and lung AC risk in Asians; *GSTT1* null genotype is associated with increased LC risk in Chinese, and *GSTP1* IIe105Val polymorphism is associated with increased LC risk in Asians.

## 1 Introduction

Lung cancer (LC) is the most common malignancy worldwide, accounting for more deaths than any other cancer in India.^[1,2]^ There were about 228,190 new LC cases and 159,480 deaths in America in 2013.^[3]^ It is calculated that over one million Chinese may be diagnosed with LC by 2025 in China.^[4]^ Up to now, it is still not clear on the mechanism of LC. Studies have indicated that smoking was one of the most important risk factors,^[5,6]^ however, only a small fraction of people, who are exposed to such risk factors, will develop LC. This indicates that host factors including genetic polymorphism may be an important role in LC development.

The glutathione S-transferases (*GSTs*) are a supergene family of phase II detoxifying enzymes, which play important role in the detoxification of toxic, potentially carcinogenic compounds, and a series of basic physiological processes of the human body.^[7–9]^ In human, *GSTs* enzymes have been observed to be five classes (α, μ, π, σ, and θ)^[10]^ and each class is encoded as an independent gene or family gene (such as *GSTA*, *GSTM*, *GSTP*, *GSTO*, and *GSTT* genes). Glutathione S-transferase M1 (*GSTM1*), glutathione S-transferase P1 (*GSTP1*), and glutathione S-transferase T1 (*GSTT1*) polymorphisms have been identified resulting in possible impaired activity for the elimination of carcinogenic compounds and raised risk of cancer.^[11]^ The *GSTM1* and *GSTT1* show deletion (null genotype), which causes enzyme activity loss.^[11]^ They are located on chromosome 1 (1p13.3) and chromosome 22 (22q11.2), respectively.^[12]^ A codon 105 A to G mutation at exon 5 in *GSTP1* polymorphism leads to change in isoleucine (IIe) to valine (Val), which also brings about decreased enzymatic activity.^[13–14]^

To date, 291 publications^[supplemental^^reference^^1–291]^ have been reported on the individual and combined effects of *GSTM1* present/null, *GSTT1* present/null, and *GSTP1* IIe105Val polymorphisms with LC risk. However, these results were still contradictory. In addition, 35 meta-analyses^[15–29,31–50]^ have been reported on the individual and the combined effects of *GSTM1* present/null, *GSTT1* present/null, and *GSTP1* IIe105Val polymorphisms with LC risk. However, a lot of studies have been published to investigate these associations recently. Hence, an updated meta-analysis should be performed to explore these problems. For all we know, this is the first meta-analysis to investigate the combined effects of *GSTM1* and *GSTP1*, *GSTT1* and *GSTP1*, and *GSTM1*, *GSTT1*, and *GSTP1* with LC risk in the overall population. Moreover, there has been no study investigating whether the previous meta-analyses are “credible” on these associations. Therefore, 2 Bayesian methods (false-positive report probability (FPRP) and Bayesian false discovery probability (BFDP)) and the Venice criteria were applied to evaluate the credibility of these findings. We aimed to provide true associations on these problems and discuss the identified positive findings in terms of biological mechanisms involved in LC.

## 2 Materials and methods

### 2.1 Search strategy

Meta-analyses of Observational Studies in Epidemiology guidelines were used.^[51]^ PubMed and China National Knowledge Infrastructure (CNKI) databases were applied to search literature in this meta-analysis (update to April 22, 2019). The following search strategy (it was designed to be sensitive and broad) was applied: (glutathione S-transferase T1 OR *GSTT1* OR glutathione S-transferase P1 OR *GSTP1* OR glutathione S-transferase M1 OR *GSTM1*) AND lung AND (polymorphism OR genotype OR allele OR variant OR mutation). In addition, the reference lists of identified articles and reviews (including published meta-analyses) were examined as appropriate. Moreover, Finally, the corresponding authors were contacted via e-mail if necessary. There was no limit or restriction on language in this study.

### 2.2 Inclusion and exclusion criteria

Inclusion criteria were as listed below: (1) case–control or cohort studies; (2) publications on *GSTM1* present/null, *GSTT1* present/null, *GSTP1* IIe105Val, and their combined effects with LC risk; and (3) complete genotype data between LC cases and controls. Exclusion criteria were as listed below: (1) duplicate genotype data; (2) no case–control studies; (3) meta-analyses, reviews, or letters; and (4) other SNP.

### 2.3 Data extraction and quality score assessment

Two authors independently collected data of all eligible studies applying Excel. If necessary, any disagreement was resolved by discussion. The following data were extracted: (1) first author's surname, (2) year of publication, (3) country, (4) ethnicity, (5) sample size, (6) cases source, (7) controls source, (8) type of controls, (9) matching, (10) material used for assessment of genotype, and (11) genotype distribution of *GSTM1* present/null, *GSTT1* present/null, *GSTP1* IIe105Val, and their combined effects in cases and controls. Races were considered as “Caucasians,” “Asians,” “Indians,” and “Africans.” “Mixed populations” was defined if race was not stated or the sample size cannot be separated. The scale of quality assessment criteria are listed by 2 previous meta-analyses^[52,53]^ in Table 12, Supplemental Digital Content. Tables 2 and 3, Supplemental Digital Content list the quality assessment by included studies. Studies scoring >12 will be considered as high quality.

### 2.4 Statistical analysis

Crude odds ratios (ORs) and their 95% confidence intervals (CIs) were used to assess the associations between the individual and combined effects of *GSTM1*, *GSTT1*, and *GSTP1* IIe105Val polymorphisms with LC risk. Either a fixed-effect model (Mantel–Haenszel method)^[54]^ or a random-effect model (DerSimonian–Laird model)^[55]^ was applied in this meta-analysis. Between-study heterogeneity was evaluated by calculating the *Q* statistic and *I*^2^ value (a random-effect model was applied if *P* < .10 and/or *I*^2^ > 50%). Subgroups were also calculated if heterogeneity was significant. In addition, we applied a meta-regression analysis to assess the source of heterogeneity. Sensitivity analysis was performed by removing a single study each time. Begg funnel plot^[56]^ and Egger regression asymmetry test^[57]^ were used to identify publication bias. A nonparametric “trim and fill” method^[58]^ was considered to add missing studies if publication bias was observed in this meta-analysis. Moreover, Chi-square goodness-of-fit test was applied to check Hardy–Weinberg equilibrium (HWE), and significant deviation was considered in control groups if *P* < .05. All statistical analyses were calculated using STATA version 12.0 (STATA Corporation, College Station, TX).

### 2.5 Credibility of genetic association

We employed FPRP,^[59]^ BFDP,^[60]^ and the Venice criteria^[61]^ to verify the credibility of current and previous meta-analyses. FPRP and BFDP can clarify the probability of no true association between genetic association and disease. The FPRP and BFDP were calculated by applying the Excel spreadsheet. A cutoff value of FPRP and BRDP was set up to be a level of 0.2 and 0.8 to assess whether the significant associations were noteworthy or not, respectively. Concerning the Venice criteria, we evaluated the criteria of the amount of evidence by statistical power^[62]^: A: 80% or more; B: 50% to 79%; and C: <50%. For replication, we applied the *I*^2^ recommended by Ioannidis et al^[61]^: A: less than 25%, B: 25% to 50%, and C: more than 50%. For protection from bias, we considered using the criteria proposed by Ioannidis et al^[61]^ The following criteria were applied to assess the credibility of genetic association by FPRP, BFDP, and the Venice criteria. Firstly, associations were considered as positive results if they met the following criteria^[62]^: (1) statistically significant associations were observed in at least 2 of the genetic model (individual *GSTM1* and *GSTT1* polymorphisms with LC risk did not need to meet the criteria); (2) FPRP < 0.2 and BFDP < 0.8; (3) *I*^2^ < 50%; and (4) statistical power >80%. All other significant results were considered as less-credible positives. Previous meta-analyses were selected to assess the credibility by the following criteria: (1) more recent meta-analysis with the larger number of participants was selected and (2) studies supplying complete information involving OR and 95% CI.

## 3 Results

### 3.1 Study characteristics

**Figure 1:**
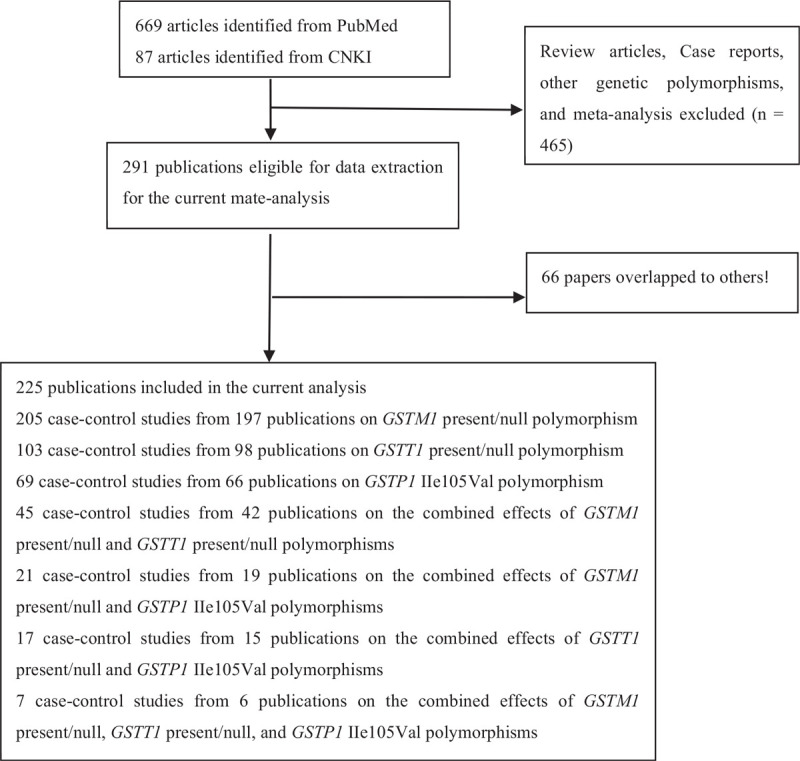
Flow diagram for identifying and including studies in the current meta-analysis.

A flowchart of study selection is listed in Figure 1. Overall, 756 publications were identified by PubMed and CNKI databases. Among these publications, 291 were selected by carefully screening titles, abstracts, and full text. In addition, 66 studies^[supplemental references 5, 11, 12, 16, 18, 23, 24, 33, 38, 48, 54, 62, 73, 86, 89, 96, 101, 103, 112, 116, 166, 173, 177, 180, 183, 187, 189, 190, 193, 195, 196, 198, 199, 202, 206, 208, 212, 217, 218, 221, 222, 223, 224, 225, 228, 233, 235, 237, 238, 239, 241, 243, 244, 245, 251, 253, 254, 264, 267, 269, 270, 272, 280, 283, 284, 285]^ were excluded because another 47 publications included their cases and controls. Finally, 225 publications met the inclusion criteria. The general characteristics of publications are listed in Table 1, Supplemental Digital Content. There were 205 case–control studies from 197 articles (involving 45,726 cases and 58,788 controls, as shown in Table 4, Supplemental Digital Content) on *GSTM1* present/null polymorphism, 103 case–control studies from 98 articles (involving 29,476 cases and 35,305 controls, as shown in Table 4, Supplemental Digital Content) on *GSTT1* present/null polymorphism, 69 case–control studies from 66 publications regarding *GSTP1* IIe105Val polymorphism (including 18,852 cases and 21,941 controls, as shown in Table 4, Supplemental Digital Content), 45 case–control studies from 42 publications on the combined effects of *GSTM1* and *GSTT1* present/null polymorphisms (involving 15,560 cases and 15,914 controls, as shown in Table 8, Supplemental Digital Content), 21 case–control studies from 19 publications on the combined effects of *GSTM1* present/null and *GSTP1* IIe105Val polymorphisms (involving 4538 cases and 5604 controls, as shown in Table 9, Supplemental Digital Content), 17 case–control studies from 15 publications regarding the combined effects of *GSTT1* present/null and *GSTP1* IIe105Val polymorphisms (involving 3507 cases and 4151 controls, as shown in Table 10, Supplemental Digital Content), and 7 case–control studies from 6 publications concerning the combined effects of the *GSTM1* present*/*null, *GSTT1* present*/*null, and *GSTP1* IIe105Val polymorphisms (including 436 cases and 672 controls, as shown in Table 11, Supplemental Digital Content) with LC risk. In addition, we also collected the genotype frequencies of the *GSTM1*, *GSTT1*, and *GSTP1* polymorphisms by histological type, smoking status, and gender, as shown in Tables 5 to 7, Supplemental Digital Content, respectively. In the end, Tables 2 and 3, Supplemental Digital Content show the quality assessment in this meta-analysis by scale for quality assessment of molecular association studies of lung cancer (as shown in Table 12, Supplemental Digital Content).

### 3.2 Quantitative synthesis

The *GSTM1* null genotype was associated with an increased LC risk (OR = 1.24, 95% CI: 1.19–1.30) in the overall analysis and some subgroups, such as Asians, Caucasians, Chinese populations, Japanese populations, and so on, as shown in Table 13, Supplemental Digital Content.

The *GSTT1* null genotype was also associated with an increased LC risk (OR = 1.16, 95% CI: 1.08–1.24) in the overall analysis and several subgroups, such as Indians, Asians, Chinese populations, Japanese populations, high-quality studies, large-sized studies, smokers, and so on, as shown in Table 14, Supplemental Digital Content.

The pooled data from all eligible studies yielded a significant association between the *GSTP1* IIe105Val polymorphism and LC risk (Val/Val + IIe/Val vs IIe/IIe: OR = 1.06, 95% CI = 1.00–2.11 and Val vs IIe: OR = 1.40, 95% CI = 1.34–1.46, Table 15, Supplemental Digital Content). In addition, a significantly increased LC risk was also found in several subgroups, such as Africans, Asians, Caucasians, and so on, as shown in Table 15, Supplemental Digital Content.

A significant association was observed (model 1: OR = 1.34, 95% CI = 1.11–1.61; model 2: OR = 1.27, 95% CI = 1.11–1.46; model 3: OR = 1.53, 95% CI = 1.30–1.80; model 4: OR = 1.20, 95% CI = 1.08–1.33; model 5: OR = 1.28, 95% CI = 1.15–1.42; and model 6: OR = 1.30, 95% CI = 1.17–1.45, Table 16, Supplemental Digital Content) between the combined effects of *GSTM1* and *GSTT1* null genotypes in the overall analysis and several subgroups, such as Caucasians, Asians, Indians, population-based studies, high-quality studies, and so on, as shown in Table 16, Supplemental Digital Content.

A significantly increased LC risk was found (model 1: OR = 1.15, 95% CI = 1.01–1.31; model 4: OR = 1.31, 95% CI = 1.09–1.56; model 5: OR = 1.18, 95% CI = 1.03–1.36; and model 6: OR = 1.20, 95% CI = 1.06–1.35, Table 17, Supplemental Digital Content) between the combined effects of *GSTM1* present/null and *GSTP1* IIe105Val polymorphisms in the overall analysis and several subgroups, such as Caucasians, Asians, Indians, Africans, and so on (Table 17, Supplemental Digital Content).

A significantly increased LC risk was observed (model 1: OR = 1.32, 95% CI = 1.10–1.58; model 4: OR = 1.55, 95% CI = 1.18–2.02; and model 6: OR = 1.47, 95% CI = 1.15–1.88) between the combined effects of *GSTT1* present/null and *GSTP1* IIe105Val polymorphism in the overall analysis and several subgroups, such as Caucasians, Asians, Indians, and so on, as shown in Table 18, Supplemental Digital Content.

Last, a significant association was observed (model 7: OR = 2.81, 95% CI = 1.02–7.79; model 8: OR = 1.44, 95% CI = 1.13–1.85; model 9: OR = 2.09, 95% CI = 1.42–3.08; and model 10: OR = 1.73, 95% CI = 1.17–2.56) between the combined effects of *GSTM1* present/null, *GSTT1* present/null and *GSTP1* IIe105Val polymorphisms when all eligible studies were pooled, as shown in Table 19, Supplemental Digital Content.

### 3.3 Heterogeneity and sensitivity analyses

Between-studies heterogeneity was observed, as shown in Tables 13 to 19, Supplemental Digital Content. A meta-regression analysis indicates that ethnicity (*P* = .006) and type of controls (*P* = .019) are sources of heterogeneity between the *GSTM1* null genotype and LC risk. For the *GSTT1* null genotype, a meta-regression analysis suggests that ethnicity (*P* = .017), source of controls (*P* < .001), and type of controls (*P* < .001) are sources of heterogeneity. We found that HWE (model 1: *P* = .046) and quality score (model 6: *P* = .043) were the sources of heterogeneity by meta-regression analysis for the combined effects of *GSTM1* present/null and *GSTP1* IIe105Val polymorphisms. Moreover, we have not observed any change when 1 single study was excluded each time in the overall analysis.

### 3.4 Evaluation of publication bias

There was obvious evidence of publication bias for *GSTM1* null genotype (*P* < .001), *GSTT1* null genotype (*P* = .044), *GSTP1* IIe105Val (Val/Val vs IIe/IIe: *P* = .010; IIe/Val vs IIe/IIe: *P* < .001; Val/Val + IIe/Val vs IIe/IIe: *P* < .001), the combined effects of *GSTM1* and *GSTT1* (model 1: *P* = .022; model 2: *P* = .013; model 3: *P* = .032; model 5: *P* = .037; and model 6: *P* = .004), the combined effects of *GSTM1* present/null and *GSTP1* IIe105Val (model 4: *P* = .001 and model 6: *P* = .002) by the Begg funnel plot shape and Egger test in the current meta-analysis. Figures 1 to 12, Supplemental Digital Content lists the funnel plots of the nonparametric “trim and fill” method. No significant association was observed (Val/Val + IIe/Val vs IIe/IIe: OR = 0.97, 95% CI = 0.92–1.03) for the *GSTP1* IIe105Val when we applied the nonparametric “trim and fill” method in the overall analysis. The results of a pooled analysis from all studies changed in the following genetic models (model 1: OR = 1.08, 95% CI = 0.89–1.31; model 2: OR = 1.03, 95% CI = 0.89–1.19; model 5: OR = 1.10, 95% CI = 0.98–1.24; and model 6: OR = 1.10, 95% CI = 0.98–1.24) for the combined effects of *GSTM1* and *GSTT1* null genotypes when we applied the nonparametric “trim and fill” method. The results of a pooled analysis from all studies changed in model 4 (OR = 1.01, 95% CI = 0.83–1.24) and model 6 (OR = 1.00, 95% CI = 0.87–1.14) when we applied the nonparametric “trim and fill” method.

### 3.5 Credibility of the previous meta-analyses

To evaluate the credibility of the previously published meta-analyses with the largest number of cases and controls on the associations between the *GSTM1* present/null, *GSTT1* present/null, and/or *GSTP1* IIe105Val polymorphisms with LC risk, we applied the FPRP, BFDP, and the Venice criteria. Table 1 shows the results of the credibility on these issues. Gao et al^[18]^ on the combined effects of *GSTM1* present/null and *GSTT1* present/null polymorphisms with LC risk will be considered as “positive” result in the overall population, Ye et al^[15]^ on the *GSTM1* null genotype with LC risk in all races, Liu et al^[41]^ on the *GSTM1* null genotype with LC risk in Chinese populations, and Xu et al^[33]^ on the *GSTP1* IIe105Val polymorphism with LC risk will be considered as “positive” results because their studies represent the most credible findings. Li et al,^[28]^ Sengupta et al,^[50]^ Yang et al,^[19]^ Yang et al,^[34]^ Wang et al,^[40]^ and Feng et al^[21]^ will be classified as less-credible results (higher heterogeneity, lower statistical power, FPRP > 0.2 and BFDP > 0.8).

**Table 1:** Credibility of previously published meta-analysis with the largest number of participants.

								Credibility			
									Prior probability of 0.001		
Author	Gene	Model	n	Case/control	Variable	OR (95% CI)	*I*^2^ (%)	Statistical power	FPRP	BFDP	Venice criteria
Gao et al^[18]^ 2017	*GSTM1-GSTT1*	Model 1	34	5886/5224	Overall	1.58 (1.34–1.87)	57.8	0.273	<0.001	0.006	CCB
Gao et al^[18]^ 2017	*GSTM1-GSTT1*	Model 2	23	3309/2063	Overall	1.26 (1.13–1.42)	4.7	0.998	0.131	0.885	AAB
Gao et al^[18]^ 2017	*GSTM1-GSTT1*	Model 3	23	4447/3198	Overall	1.26 (1.08–1.48)	31.5	0.983	0.832	0.993	ABB
Gao et al^[18]^ 2017	*GSTM1-GSTT1*	Model 4	34	8177/6586	Overall	1.27 (1.13–1.42)	28.2	**0.998**	**0.026**	**0.619**	**ABB**
Gao et al^[18^] 2017	*GSTM1-GSTT1*	Model 5	44	13,706/13,093	Overall	1.33 (1.19–1.48)	45.9	**0.986**	**<0.001**	**0.013**	**ABB**
Gao et al^[18^] 2017	*GSTM1-GSTT1*	Model 1	16	2608/2893	Caucasian	1.23 (1.07–1.41)	12	0.998	0.748	0.991	AAB
Gao et al^[18]^ 2017	*GSTM1-GSTT1*	Model 1	3	348/391	Indian	2.53 (1.61–3.98)	0.0	0.012	0.833	0.727	CAB
Gao et al^[18]^ 2017	*GSTM1-GSTT1*	Model 4	3	348/273	Indian	1.69 (1.07–2.67)	2.0	0.305	0.988	0.997	CAB
Gao et al^[18]^ 2017	*GSTM1-GSTT1*	Model 5	3	632/632	Indian	2.11 (1.36–3.28)	1.2	0.065	0.933	0.959	CAB
Gao et al^[18]^ 2017	*GSTM1-GSTT1*	Model 3	10	2948/1592	Asian	1.24 (1.10–1.41)	33.2	0.998	0.508	0.977	ABB
Gao et al^[18]^ 2017	*GSTM1-GSTT1*	Model 4	11	4159/2403	Asian	1.45 (1.19–1.77)	39.8	0.631	0.292	0.898	BBB
Gao et al^[18]^ 2017	*GSTM1-GSTT1*	Model 5	14	5766/4337	Asian	1.53 (1.24–1.90)	68.1	0.429	0.217	0.806	CCB
Li et al^[28]^ 2015	*GSTM1-GSTP1*	Model a	2	209/316	Chinese	1.68 (1.08–2.60)	NA	0.306	0.985	0.996	C-B
Li et al^[28]^ 2015	*GSTM1-GSTP1*	Model b	2	209/316	Chinese	2.13 (1.27–3.56)	NA	0.090	0.977	0.987	C-B
Li et al^[28]^ 2015	*GSTM1-GSTP1*	Model c	2	209/316	Chinese	2.29 (1.33–3.93)	NA	0.062	0.977	0.983	C-B
Ye et al^[15]^ 2006	*GSTM1*	null vs present	119	19,729/25,931	Overall	1.22 (1.14–1.23)	44	**1.000**	**<0.001**	**<0.001**	**ABB**
Liu et al^[41]^ 2014	*GSTM1*	null vs present	68	8649/10,380	Chinese	1.20 (1.16–1.25)	45.1	**1.000**	**<0.001**	**<0.001**	**ABB**
Sengupta et al^[50]^ 2017	*GSTM1*	null vs present	13	NA	Indian	1.30 (1.01–1.68)	11.9	0.863	0.981	0.998	AAB
Yang et al^[19]^ 2014	*GSTT1*	null vs present	55	15,140/16,662	Overall	1.14 (1.03–1.25)	62.8	1.000	0.841	0.996	ACB
Yang et al^[34]^ 2013	*GSTT1*	null vs present	23	4065/5390	Asian	1.28 (1.10–1.49)	62.0	0.980	0.596	0.981	ACB
Wang et al^[40]^ 2015	*GSTT1*	null vs present	20	3351/4683	Chinese	1.31 (1.12–1.52)	59	0.963	0.278	0.937	ACB
Sengupta et al^[50]^ 2017	*GSTT1*	null vs present	12	NA	Indian	1.39 (1.04–1.87)	34.7	0.693	0.977	0.998	BBB
Li et al^[28]^ 2015	*GSTP1*	Val/Val vs IIe/IIe	13	2026/2451	Chinese	1.36 (1.01–1.84)	31.7	0.737	0.984	0.998	BBB
Xu et al^[33]^ 2014	*GSTP1*	Val/Val vs IIe/IIe	18	3175/5516	Asian	1.22 (1.16–1.59)	NA	0.937	0.993	0.999	A-B
Xu et al^[33]^ 2014	*GSTP1*	Val/Val + IIe/Val vs IIe/IIe	18	3175/5516	Asian	1.24 (1.12–1.37)	18.4	**1.000**	**0.023**	**0.609**	**AAB**
Feng et al^[21]^ 2012	*GSTP1*	Val vs IIe	44	12,363/13,948	Overall	1.08 (1.02–1.15)	44	1.000	0.942	0.999	ABB

CI = confidence interval, OR = odds ratio, SC = squamous carcinoma, Model a = *M1* null/*P1* IIe/IIe vs *M1* present/*P1* IIe/IIe, Model b = *M1 *null/*P1* Val^∗^ vs *M1* present/*P1* IIe/IIe, Model c = T*1 *null/*P1* Val^∗^ vs T*1* present/*P1* IIe/IIe, Model 1 = M1 null/T1 null vs M1 present/T1 present, Model 2 = M1 null/T1 null vs M1 present/T1 null, Model 3 = M1 null/T1 null vs M1 null/T1 present, Model 4 = M1 null/T1 null vs All 1 risk genotypes, Model 5 = M1 null/T1 null vs (M1 present/T1 present + M1 present/T1 null + M1 null/T1 present). The significance of bold values indicated that these positive results were credible.

### 3.6 Credibility of the current meta-analysis

To evaluate the credibility of the present meta-analysis, we also applied the FPRP, BFDP, and the Venice criteria. Table 2 lists the credibility of the current meta-analysis on the individual and combined effects of *GSTM1* present/null, *GSTT1* present/null, and *GSTP1* IIe105Val polymorphisms with LC risk. They will be considered as “positive” results on the *GSTM1* null genotype with LC risk in Japanese population (OR = 1.30, 95% CI = 1.17–1.44, *I*^2^ = 0.0%, statistical power = 0.997, FPRP = 0.008, BFDP = 0.037, and Venice criteria: AAB), *GSTM1* null genotype with lung AC risk in Asians (OR = 1.35, 95% CI = 1.22–1.48, *I*^2^ = 25.5%, statistical power = 0.988, FPRP < 0.001, BFDP < 0.001, and Venice criteria: ABB), *GSTT1* null genotype with LC risk in Asians (OR = 1.23, 95% CI = 1.12–1.36, *I*^2^ = 49.1%, statistical power = 1.000, FPRP = 0.051, BFDP = 0.771, and Venice criteria: ABB), especially in Chinese population (OR = 1.31, 95% CI = 1.16–1.49, *I*^2^ = 48.9%, statistical power = 0.980, FPRP = 0.039, BFDP = 0.673, and Venice criteria: ABB), *GSTT1* null genotype with lung AC risk in Asians (OR = 1.36, 95% CI = 1.17–1.58, *I*^2^ = 30.2%, statistical power = 0.900, FPRP = 0.061, BFDP = 0.727, and Venice criteria: ABB), and *GSTP1* IIe105Val polymorphism with LC risk in overall population, especially in Asians (Val vs IIe: OR = 1.28, 95% CI = 1.17–1.42, *I*^2^ = 30.3%, statistical power = 0.999, FPRP = 0.003, BFDP = 0.183, and Venice criteria: ABB). All other significant associations will be considered as less-credible results, as also shown in Table 2.

**Table 2:** Credibility of the current meta-analysis.

				Credibility			
					Prior probability of 0.001		
Variables	Model	OR (95% CI)	*I*^2^ (%)	Statistical power	FPRP	BFDP	Venice criteria
*GSTM1*							
Overall	Null vs present	1.24 (1.19–1.30)	58.5	1.000	<0.001	<0.001	ACB
Asian	Null vs present	1.43 (1.33–1.53)	54.8	0.988	<0.001	<0.001	ACB
Caucasian	Null vs present	1.07 (1.01–1.13)	39.4	1.000	0.938	0.999	ABB
China	Null vs present	1.52 (1.40–1.65)	53.3	1.000	<0.001	<0.001	ACB
Japan	Null vs present	**1.30 (1.17–1.44)**	**0.0**	**0.997**	**0.008**	**0.037**	AAB
HB	Null vs present	1.30 (1.21–1.39)	64.0	1.000	<0.001	<0.001	ACB
PB	Null vs present	1.14 (1.05–1.24)	55.6	1.000	0.718	0.992	ACB
Matching	Null vs present	1.18 (1.10–1.25)	55.1	1.000	<0.001	0.003	ACB
Non-matching	Null vs present	1.30 (1.23–1.39)	60.8	1.000	<0.001	<0.001	ACB
Quality score >12	Null vs present	1.14 (1.07–1.21)	57.8	1.000	0.017	0.637	ACB
Quality score ≤12	Null vs present	1.31 (1.24–1.39)	56.9	1.000	<0.001	<0.001	ACB
Sample size >200	Null vs present	1.21 (1.16–1.27)	63.2	1.000	<0.001	<0.001	ACB
Sample size ≤200	Null vs present	**1.42 (1.29–1.57)**	**18.8**	**0.858**	**<0.001**	**<0.001**	AAB
SCLC	Null vs present	1.38 (1.16–1.63)	50.2	0.837	0.152	0.855	ACB
SCLC/Asian	Null vs present	1.43 (1.04–1.97)	43.3	0.615	0.979	0.997	BBB
SCLC/Caucasian	Null vs present	1.33 (1.01–1.76)	65.7	0.800	0.983	0.998	ACB
SCLC/Indian	Null vs present	1.66 (1.21–2.28)	0.0	0.266	0.868	0.975	CAB
SC	Null vs present	1.33 (1.22–1.45)	55.2	0.997	<0.001	<0.001	ACB
SC/Asian	Null vs present	1.52 (1.38–1.66)	10.9	0.403	<0.001	<0.001	CAB
SC/Indian	Null vs present	1.37 (1.13–1.67)	0.0	0.815	0.692	0.981	AAB
AC	Null vs present	1.24 (1.13–1.36)	52.0	1.000	0.005	0.277	ACB
AC/Asian	Null vs present	**1.35 (1.22–1.48)**	**25.5**	**0.988**	**<0.001**	**<0.001**	ABB
AC/Indian	Null vs present	1.49 (1.17–1.90)	19.2	0.522	0.714	0.971	BAB
Smoking	Null vs present	1.27 (1.17–1.39)	61.7	1.000	<0.001	0.019	ACB
Non-smoking	Null vs present	1.36 (1.21–1.53)	50.4	0.948	<0.001	0.022	ACB
Male	Null vs present	1.16 (1.06–1.26)	47.1	1.000	0.303	0.966	ABB
*GSTT1*							
Overall	Null vs present	1.16 (1.08–1.24)	59.2	1.000	0.013	0.558	ACB
Indian	Null vs present	1.54 (1.13–2.11)	78.5	0.435	0.943	0.992	CCB
Asian	Null vs present	**1.23 (1.12–1.36)**	**49.1**	**1.000**	**0.051**	**0.771**	**ABB**
China	Null vs present	**1.31 (1.16–1.49)**	**48.9**	**0.980**	**0.039**	**0.673**	**ABB**
Japan	Null vs present	1.22 (1.01–1.47)	8.2	0.985	0.974	0.999	AAB
North India	Null vs present	2.99 (1.88–4.78)	51.8	0.002	0.706	0.267	CCB
HB	Null vs present	1.17 (1.06–1.29)	63.1	1.000	0.619	0.988	ACB
Matching	Null vs present	1.12 (1.02–1.24)	56.3	1.000	0.967	0.999	ACB
Non-matching	Null vs present	1.19 (1.08–1.30)	61.9	1.000	0.103	0.885	ACB
Quality score >12	Null vs present	1.11 (1.02–1.21)	54.8	1.000	0.947	0.999	ACB
Quality score ≤12	Null vs present	1.20 (1.08–1.33)	62.0	1.000	0.339	0.964	ACB
Sample size >200	Null vs present	1.15 (1.08–1.23)	60.7	1.000	0.044	0.808	ACB
LCLC	Null vs present	0.39 (0.17–0.94)	36.3	0.114	0.997	0.998	CBB
SC/Asian	Null vs present	1.38 (1.02–1.87)	63.5	0.705	0.982	0.998	BCB
AC/Asian	Null vs present	**1.36 (1.17–1.58)**	**30.2**	**0.900**	**0.061**	**0.727**	**ABB**
AC/Indian	Null vs present	2.02 (1.51–2.70)	0.0	0.022	0.084	0.094	CAB
Smoking	Null vs present	1.23 (1.08–1.40)	56.1	0.999	0.633	0.985	ACB
*GSTP1*							
Overall	Val/Val + IIe/Val vs IIe/IIe	1.06 (1.00–1.11)	29.0	1.000	0.930	0.999	ABB
	Val vs IIe	**1.40 (1.34–1.46)**	**23.3**	**1.000**	**<0.001**	**<0.001**	**AAB**
African	Val vs IIe	1.65 (1.27–2.15)	0.0	0.240	0.465	0.865	CAB
Asian	Val/Val vs IIe/IIe	1.45 (1.16–1.80)	7.6	0.621	0.549	0.957	BAB
	IIe/Val vs IIe/IIe	1.13 (1.02–1.24)	12.0	1.000	0.908	0.998	AAB
	Val/Val vs IIe/IIe + IIe/Val	1.39 (1.12–1.72)	0.0	0.758	0.763	0.984	BAB
	Val/Val + IIe/Val vs IIe/IIe	1.16 (1.06–1.26)	23.0	1.000	0.303	0.966	AAB
	Val vs IIe	**1.28 (1.17–1.42)**	**30.3**	**0.999**	**0.003**	**0.183**	**ABB**
Caucasian	Val vs IIe	1.44 (1.38–1.50)	0.0	1.000	<0.001	<0.001	AAB
HB	Val/Val vs IIe/IIe	1.12 (1.01–1.25)	11.5	1.000	0.977	0.999	AAB
	Val/Val vs IIe/IIe + IIe/Val	1.12 (1.01–1.24)	6.0	1.000	0.967	0.999	AAB
	Val/Val + IIe/Val vs IIe/IIe	1.08 (1.01–1.16)	35.5	1.000	0.972	0.999	ABB
	Val vs IIe	**1.38 (1.31–1.47)**	**26.4**	**1.000**	**<0.001**	**<0.001**	**ABB**
PB	Val vs IIe	1.42 (1.34–1.51)	2.5	1.000	<0.001	<0.001	AAB
Matching	Val vs IIe	1.38 (1.32–1.45)	18.4	1.000	<0.001	<0.001	AAB
Non-matching	Val vs IIe	1.42 (1.33–1.51)	29.0	1.000	<0.001	<0.001	ABB
Quality score >12	Val vs IIe	1.40 (1.34–1.46)	0.0	1.000	<0.001	<0.001	AAB
Quality score ≤12	Val/Val vs IIe/IIe	1.23 (1.06–1.42)	9.8	0.997	0.826	0.993	AAB
	IIe/Val vs IIe/IIe	1.13 (1.05–1.23)	24.6	1.000	0.825	0.996	AAB
	Val/Val vs IIe/IIe + IIe/Val	1.16 (1.01–1.34)	1.8	1.000	0.978	0.999	AAB
	Val/Val + IIe/Val vs IIe/IIe	1.16 (1.07–1.25)	26.5	1.000	0.090	0.886	ABB
	Val vs IIe	**1.39 (1.27–1.51)**	**44.1**	**0.964**	**<0.001**	**<0.001**	**ABB**
Sample size >200	Val/Val + IIe/Val vs IIe/IIe	1.06 (1.00–1.12)	32.9	1.000	0.974	1.000	ABB
	Val vs IIe	**1.41 (1.35–1.47)**	**23.2**	**1.000**	**<0.001**	**<0.001**	**ABB**
HWE (yes)	Val/Val vs IIe/IIe	1.08 (1.00–1.17)	17.6	1.000	0.983	1.000	AAB
	Val vs IIe	**1.41 (1.36–1.46)**	**9.6**	**1.000**	**<0.001**	**<0.001**	**AAB**
HWE (no)	Val/Val vs IIe/IIe	0.73 (0.54–0.99)	0.0	0.709	0.984	0.998	BAB
	Val/Val vs IIe/IIe + IIe/Val	0.71 (0.53–0.95)	0.0	0.652	0.970	0.997	BAB
SCLC	Val/Val vs IIe/IIe	1.34 (1.01–1.77)	0.0	0.787	0.980	0.998	BAB
	Val/Val vs IIe/IIe + IIe/Val	1.32 (1.01–1.72)	21.8	0.828	0.980	0.998	AAB
SCLC/Caucasian	Val/Val vs IIe/IIe	1.42 (1.05–1.92)	0.0	0.639	0.973	0.997	BAB
	Val/Val vs IIe/IIe + IIe/Val	1.41 (1.07–1.87)	20.6	0.666	0.962	0.996	BAB
Smoking	Val/Val vs IIe/IIe	1.33 (1.08–1.64)	0.0	0.870	0.898	0.994	AAB
	Val/Val vs IIe/IIe + IIe/Val	1.29 (1.01–1.57)	0.0	0.934	0.922	0.996	AAB
	Val vs IIe	1.10 (1.01–1.21)	0.0	1.000	0.980	0.999	AAB
The combined effects of *GSTM1* and *GSTT1* polymorphisms							
Overall	Model 1	1.34 (1.11–1.61)	54.7	0.886	0.667	0.981	ACB
	Model 2	1.27 (1.11–1.46)	57.0	0.990	0.440	0.968	ACB
	Model 3	1.53 (1.30–1.80)	61.6	0.406	0.001	0.017	CCB
	Model 4	1.20 (1.08–1.33)	51.5	1.000	0.339	0.964	ACB
	Model 5	1.28 (1.15–1.42)	61.3	0.999	0.003	0.183	ACB
	Model 6	1.30 (1.17–1.45)	50.6	0.995	0.002	0.147	ACB
Caucasian	Model 3	1.14 (1.02–1.28)	20.5	1.000	0.964	0.999	AAB
	Model 5	1.14 (1.02–1.27)	43.3	1.000	0.946	0.998	ABB
Asian	Model 1	1.40 (1.06–1.84)	47.1	0.690	0.958	0.996	BBB
	Model 2	1.52 (1.17–1.98)	48.2	0.461	0.805	0.978	CBB
	Model 3	1.99 (1.40–2.85)	75.6	0.061	0.738	0.846	CCB
	Model 4	1.40 (1.10–1.79)	56.8	0.709	0.911	0.993	BCB
	Model 5	1.61 (1.22–2.11)	69.9	0.304	0.647	0.938	CCB
	Model 6	1.51 (1.22–1.86)	68.1	0.475	0.183	0.793	CCB
Indian	Model 2	1.53 (1.13–2.07)	0.0	0.449	0.928	0.991	CAB
	Model 3	2.53 (1.61–3.98)	0.0	0.012	0.833	0.727	CAB
	Model 4	1.49 (1.18–1.88)	0.0	0.522	0.597	0.956	BAB
	Model 5	1.62 (1.29–2.02)	0.0	0.247	0.069	0.427	CAB
	Model 6	2.11 (1.36–3.28)	1.2	0.065	0.933	0.959	CAB
HB	Model 1	1.30 (1.01–1.68)	42.2	0.863	0.981	0.998	ABB
	Model 2	1.36 (1.12–1.66)	48.2	0.832	0.750	0.985	ABB
	Model 3	1.57 (1.27–1.94)	45.1	0.336	0.080	0.539	CBB
	Model 4	1.24 (1.06–1.45)	50.3	0.991	0.876	0.995	ACB
	Model 5	1.32 (1.13–1.55)	55.8	0.941	0.428	0.962	ACB
	Model 6	**1.36 (1.18–1.57)**	**39.2**	**0.909**	**0.029**	**0.572**	**ABB**
PB	Model 1	1.73 (1.13–2.65)	75.1	0.256	0.979	0.994	CCB
	Model 3	1.54 (1.12–2.13)	76.1	0.437	0.954	0.994	CCB
	Model 5	1.25 (1.02–1.53)	70.3	0.961	0.969	0.998	ACB
	Model 6	1.27 (1.05–1.53)	64.1	0.960	0.925	0.996	ACB
Matching	Model 3	1.43 (1.04–1.97)	51.7	0.615	0.979	0.997	BCB
	Model 5	1.34 (1.01–1.78)	70.9	0.782	0.982	0.998	BCB
Non-matching	Model 1	1.34 (1.10–1.64)	49.0	0.863	0.840	0.991	ABB
	Model 2	1.21 (1.06–1.38)	40.1	0.999	0.818	0.994	ABB
	Model 3	1.57 (1.29–1.91)	66.0	0.324	0.020	0.230	CCB
	Model 4	1.16 (1.05–1.28)	38.9	1.000	0.757	0.993	ABB
	Model 5	1.25 (1.11–1.40)	56.8	0.999	0.102	0.860	ACB
	Model 6	1.39 (1.20–1.61)	60.1	0.845	0.013	0.366	ACB
Quality score >12	Model 2	1.24 (1.01–1.55)	78.4	0.953	0.984	0.999	ACB
	Model 3	1.50 (1.17–1.92)	71.8	0.500	0.720	0.970	BCB
	Model 4	1.19 (1.00–1.41)	69.0	0.996	0.978	0.999	ACB
	Model 5	1.27 (1.06–1.52)	74.8	0.965	0.904	0.996	ACB
	Model 6	1.25 (1.08–1.45)	55.4	0.992	0.764	0.991	ACB
Quality score ≤12	Model 1	1.39 (1.06–1.84)	37.4	0.703	0.968	0.997	BBB
	Model 2	1.28 (1.10–1.49)	0.0	0.980	0.596	0.981	AAB
	Model 3	1.56 (1.24–1.96)	47.5	0.368	0.267	0.819	CBB
	Model 4	1.17 (1.07–1.28)	10.0	1.000	0.381	0.973	AAB
	Model 5	1.28 (1.13–1.46)	32.5	0.991	0.192	0.915	ABB
	Model 6	1.36 (1.15–1.61)	47.1	0.872	0.289	0.928	ABB
Sample size >200	Model 1	1.37 (1.11–1.69)	64.1	0.801	0.804	0.988	ACB
	Model 2	1.25 (1.08–1.46)	65.7	0.989	0.831	0.993	ACB
	Model 3	1.53 (1.29–1.83)	66.5	0.414	0.008	0.139	CCB
	Model 4	1.19 (1.07–1.33)	58.6	1.000	0.685	0.990	ACB
	Model 5	1.27 (1.13–1.42)	66.8	0.998	0.020	0.619	ACB
	Model 6	1.27 (1.14–1.42)	53.0	0.998	0.026	0.619	ACB
Sample size ≤200	Model 2	1.49 (1.02–2.20)	0.0	0.513	0.989	0.998	BAB
	Model 3	1.48 (1.01–2.17)	18.6	0.527	0.988	0.998	BAB
	Model 5	1.39 (1.01–1.92)	0.0	0.678	0.985	0.998	BAB
	Model 6	1.52 (1.16–1.99)	63.0	0.462	0.834	0.981	CCB
The combined effects of *GSTM1* and *GSTP1* polymorphisms							
Overall	Model a	1.15 (1.01–1.31)	24.9	1.000	0.973	0.999	AAB
	Model d	1.31 (1.09–1.56)	46.8	0.936	0.723	0.986	ABB
	Model e	1.18 (1.03–1.36)	51.7	1.000	0.957	1.000	ACB
	Model f	1.20 (1.06–1.35)	30.0	1.000	0.707	0.990	ABB
Caucasian	Model d	1.21 (1.00–1.47)	41.2	0.985	0.982	0.999	ABB
	Model f	1.16 (1.01–1.35)	39.6	1.000	0.982	0.999	ABB
Asian	Model a	1.68 (1.08–2.60)	0.0	0.306	0.985	0.996	CAB
	Model c	1.56 (1.03–2.35)	0.0	0.426	0.987	0.997	CAB
	Model d	2.54 (1.50–4.33)	0.0	0.026	0.959	0.952	CAB
	Model e	1.76 (1.19–2.60)	0.0	0.211	0.955	0.988	CAB
	Model f	1.90 (1.20–3.03)	0.0	0.160	0.978	0.992	CAB
Indian	Model a	1.44 (1.09–1.90)	48.8	0.614	0.942	0.994	BBB
African	Model c	1.99 (1.00–3.94)	46.8	0.209	0.996	0.998	CBB
	Model e	1.98 (1.02–3.86)	43.4	0.207	0.995	0.998	CBB
PB	Model c	1.43 (1.05–1.94)	12.5	0.621	0.972	0.997	BAB
	Model d	1.46 (1.04–2.05)	0.0	0.562	0.981	0.997	BAB
	Model e	1.44 (1.08–1.93)	18.0	0.608	0.960	0.996	BAB
Matching	Model a	1.34 (1.12–1.61)	37.9	0.886	0.667	0.981	ABB
	Model c	1.32 (1.09–1.61)	46.7	0.896	0.873	0.993	ABB
	Model d	1.55 (1.17–2.06)	56.5	0.411	0.860	0.982	CCB
	Model e	1.39 (1.14–1.71)	55.2	0.764	0.706	0.980	BCB
	Model f	1.28 (1.05–1.57)	45.6	0.936	0.950	0.997	ABB
Quality score >12	Model a	1.32 (1.01–1.71)	53.6	0.833	0.977	0.998	ACB
	Model c	1.26 (1.05–1.52)	47.6	0.966	0.942	0.997	ABB
	Model d	1.31 (1.02–1.68)	52.5	0.857	0.975	0.998	ACB
	Model e	1.29 (1.06–1.57)	56.9	0.934	0.922	0.996	ACB
Quality score ≤12	Model d	1.30 (1.07–1.58)	39.1	0.925	0.901	0.995	ABB
	Model f	1.34 (1.14–1.57)	0.0	0.919	0.242	0.919	AAB
HWE (yes)	Model d	1.34 (1.10–1.62)	51.0	0.878	0.740	0.986	ACB
	Model e	1.17 (1.02–1.34)	42.5	1.000	0.959	0.998	ABB
	Model f	1.22 (1.07–1.39)	36.7	0.999	0.738	0.990	ABB
The combined effects of *GSTT1* and *GSTP1* polymorphisms							
Overall	Model g	1.32 (1.10–1.58)	0.0	0.918	0.729	0.986	AAB
	Model h	1.55 (1.18–2.02)	53.7	0.404	0.745	0.967	CCB
	Model k	1.47 (1.15–1.88)	52.7	0.564	0.792	0.981	BCB
Caucasian	Model h	1.42 (1.03–1.95)	50.2	0.633	0.979	0.997	BCB
	Model k	1.41 (1.03–1.93)	55.8	0.650	0.980	0.998	BCB
Asian	Model h	2.29 (1.33–3.93)	0.0	0.062	0.977	0.983	CAB
	Model j	1.47 (1.01–2.14)	0.0	0.542	0.988	0.998	BAB
Indian	Model g	1.75 (1.21–2.55)	20.5	0.211	0.944	0.986	CAB
HB	Model g	1.32 (1.06–1.64)	14.5	0.876	0.933	0.996	AAB
	Model h	1.54 (1.01–2.37)	67.9	0.452	0.991	0.998	CCB
	Model k	1.50 (1.03–2.18)	62.8	0.500	0.985	0.998	BCB
PB	Model h	1.70 (1.16–2.49)	26.7	0.260	0.961	0.991	CBB
Matching	Model h	1.41 (1.02–1.95)	40.5	0.646	0.983	0.998	BBB
Non-matching	Model g	1.50 (1.11–2.01)	0.0	0.500	0.930	0.992	BAB
	Model h	1.71 (1.09–2.67)	63.4	0.282	0.985	0.996	CCB
	Model k	1.76 (1.18–2.61)	57.4	0.213	0.958	0.989	CCB
Quality score >12	Model h	1.52 (1.09–2.12)	50.1	0.469	0.967	0.995	CCB
	Model k	1.43 (1.04–1.99)	55.6	0.612	0.982	0.998	BCB
Quality score ≤12	Model g	1.47 (1.11–1.94)	0.5	0.557	0.921	0.992	BAB
	Model k	1.53 (1.03–2.26)	50.4	0.460	0.986	0.997	CCB
HWE (yes)	Model g	1.29 (1.06–1.58)	0.0	0.928	0.937	0.997	AAB
	Model h	1.58 (1.18–2.10)	56.5	0.360	0.819	0.974	CCB
	Model k	1.48 (1.13–1.93)	56.7	0.539	0.876	0.988	BCB
The combined effects of *GSTT1* and *GSTP1* polymorphisms							
Overall	Model 7	2.81 (1.02–7.79)	–	0.114	0.998	0.998	C–B
	Model 8	1.44 (1.13–1.85)	0.0	0.625	0.874	0.989	BAB
	Model 9	2.09 (1.42–3.08)	0.0	0.047	0.806	0.862	CAB
	Model 10	1.73 (1.17–2.56)	0.0	0.238	0.963	0.991	CAB
HWE (yes)	Model 9	2.10 (1.41–3.14)	0.0	0.051	0.856	0.901	CAB

Model 1 = M1 present/T1 null vs M1 present/T1 present, Model 2 = M1 null/T1 present vs M1 present/T1 present, Model 3 = M1 null/T1 null vs M1 present/T1 present, Model 4 = all 1 risk genotypes vs M1 present/T1 present, Model 5 = all risk genotypes vs M1 present/T1 present, Model 6 = M1 null/T1 null vs M1 present/T1 present + M1 present/T1 null + M1 null/T1 present, Model a = M1 null/P1 IIe/IIe vs M1 present/P1 IIe/IIe, Model c = (M1 null/P1 IIe/IIe + M1 present/P1 Val^∗^) vs M1 present/P1 IIe/IIe, Model d = M1 null/P1 Val^∗^ vs M1 present/P1 IIe/IIe, Model e = all risk genotypes vs M1 present/P1 IIe/IIe, Model f = M1 null/P1 Val^∗^ vs (M1 present/P1 IIe/IIe + M1 null/P1 IIe/IIe + M1 present/P1 Val^∗^), Model g = T1 null/P1 IIe/IIe vs T1 present/P1 IIe/IIe, Model h = T1 null/P1 Val^∗^ vs T1 present/P1 IIe/IIe, Model j = all risk genotypes vs T1 present/P1 IIe/IIe, Model k = T1 null/P1 Val^∗^ vs (T1 present/P1 IIe/IIe + T1 null/P1 IIe/IIe + T1 present/P1 Val^∗^), Model 7 = M1 present/T1 null/P1 Val 1 vs M1 present/T1 present/P1 IIe/IIe, Model 8 = all 2 high-risk genotype vs M1 present/T1 present/P1 IIe/IIe, Model 9 = M1 null/T1 null/P1 Val1 vs M1 present/T1 present/P1 IIe/IIe, Model 10 = M1 null/T1 null/P1 Val1 vs (M1 present/T1 present/P1 IIe/IIe + all 1 high-risk genotype + all 2 high-risk genotypes). CI = confidence interval, HB = hospital-based studies, HWE = Hardy–Weinberg equilibrium, LC = lung cancer, LCLC = large cell lung carcinoma, ORs = odds ratios, PB = population-based studies, SC = squamous carcinoma, SCLC = small-cell lung cancer. The significance of bold values indicated that these positive results were credible.

## 4 Discussion

To the best of our knowledge, we reported the first meta-analysis to investigate the combined effects of *GSTM1* and *GSTP1*, *GSTT1* and *GSTP1*, and *GSTM1*, *GSTT1*, and *GSTP1* IIe105Val polymorphisms with LC risk in the overall population. We also firstly reported the credibility of these genetic polymorphisms with LC risk using the FPRP, BFDP, and the Venice criteria.

Overall, a statistically significantly increased LC risk was observed in both individual and combined effects of the *GSTM1*, *GSTT1*, and *GSTP1* polymorphisms in the current meta-analysis. However, the pooled *P* value must be adjusted because the present meta-analysis applied several subgroup analyses and genetic models at the expense of multiple comparisons.^[63]^ In addition, random error and bias were common in the studies with small sample sizes so that the results were unreliable, especially in molecular epidemiological studies. Furthermore, small sample studies were easier to accept if there were positive reports as they tend to yield false-positive results because they may be not rigorous and are often of low quality. Figures 1 to 12, Supplemental Digital Content indicated that the asymmetry of the funnel plot was caused by a study of low-quality small samples. FPRP was reported to be an appropriate approach for assessing the probability of a positive result, “noteworthiness,” on the multiple hypothesis testing of molecular epidemiology studies.^[59]^ Wakefield^[60]^ in 2007 proposed a more precise Bayesian measure of false discovery in genetic epidemiology studies, for determining the “noteworthiness” of the positive association.^[60]^ Hence, we considered FPRP and BFDP test to assess the false discovery in the current meta-analysis. Lack of replication or higher between-study heterogeneity (*I*^2^ > 50%) may be potential errors and biases, including genotype error, phenotype misclassification, population stratification, and selective reporting biases.^[64–67]^ In addition, statistical power was also an important influence factor. A large amount of evidence (statistical power >80%) can reach a more stringent level of statistical significance or decreased lower false-discovery rate.^[9]^ Therefore, we also applied for the Venice criteria to assess the credibility of the current meta-analysis.

Based on biochemical properties described for *GSTM1*, *GSTT1*, and *GSTP1* polymorphisms, we expected that the individual and the combined effects of these genes were associated with the risk of LC risk in all races. However, the significant associations were considered in the Japanese population on the *GSTM1* null genotype with LC risk, Asians on *GSTM1* null genotype with lung AC risk, Chinese population on *GSTT1* null genotype with LC risk, *GSTT1* null genotype with lung AC risk in Asians, and Asians on *GSTP1* IIe105Val polymorphism with LC risk as “highly credible” or “positive” results when we applied the FPRP, BFDP, and the Venice criteria to assess the credibility. These results indicated that the same genes may play different roles in cancer susceptibility in different races and countries, because cancer is a complicated multi genetic disease, and different genetic backgrounds and environmental factors (smoking or lifestyle) may contribute to the discrepancy.^[30]^ It was a pity that all other significant associations were considered as “less-credible” (higher heterogeneity, lower statistic power, FPRP > 0.2 and BRDP > 0.8), such as the combined effects of *GSTM1* and *GSTT1* polymorphisms, *GSTM1* and *GSTP1* polymorphisms, *GSTT1* and *GSTP1* polymorphisms, *GSTM1*, *GSTT1*, and *GSTP1* polymorphisms with lung cancer risk, and so on. These results indicated that potential gene–gene interactions are still required to investigate by a very much larger sample size. In addition, *GSTM1* present/null (Table 13, Supplemental Digital Content) and GSTP1 IIe105Val (Table 15, Supplemental Digital Content) polymorphisms were not associated with LCLC risk, however, *GSTT1* present/null was associated with LCLC risk (OR = 0.39, 95% CI = 0.17–0.94, Table 14, Supplemental Digital Content) in this meta-analysis.

We found that 8 studies only included 108 LCLC cases on *GSTM1* present/null polymorphism, 3 studies only included 51 LCLC cases on *GSTT1* present/null polymorphism, and 4 studies only included 193 LCLC cases on *GSTP1* IIe105Val polymorphism. The results might be unreliable because random error and bias were common in the pooled meta-analysis with small sample sizes. Therefore, the results should be interpreted with caution and it is necessary that a well-designed large sample study to explore the true association on the 3 genetic polymorphisms with LCLC risk.

A total of 35 published meta-analyses^[15–29,31–50]^ from 1995 to 2017 had been reported to investigate the individual and combined effects of *GSTM1* present/null, *GSTT1* present/null, and/or *GSTP1* IIe105Val polymorphisms with LC risk. Several previous meta-analyses^[15,18,19,21,28,33,34,40,41,50]^ indicated that the *GSTM1* null genotype, *GSTT1* null genotype, *GSTP1* IIe105Val, the combined effects of *GSTM1* present/null and *GSTT1* present/null polymorphisms, and the combined effects of *GSTM1* and *GSTP1* were associated with significantly increased LC risk. However, when we applied the FPRP, BFDP, and the Venice criteria to evaluate the credibility of these meta-analyses, only 3 studies^[18,33,41]^ were considered as “positive” results. In addition, a lot of studies did not be involved in the previously published meta-analysis, therefore their meta-analyses^[18,33,41]^ are still not credible.

The present study has several limitations. First, only published studies were included in the current meta-analysis while positive results are known to be published more readily than negative ones. If negative results were included, an underestimation of the *GSTM1* null effect may be observed. Second, we did not consider whether the genotype distribution in the controls was in HWE for *GSTM1* and *GSTT1* polymorphism because we cannot calculate the HWE on both genes. The current study also has several advantages over previously published meta-analyses.^[15–29,31–50]^ First, the sample size was larger. There were 205 studies (45,726 LC cases and 58,788 controls for the *GSTM1* null genotype, 103 studies (29,476 LC cases and 35,305 controls) for the *GSTT1* null genotype, 69 studies (18,852 LC cases and 21,941 controls) for the *GSTP1* IIe105Val polymorphism, and so on. Second, this is the first meta-analysis to investigate the combined effects of the 3 gene polymorphisms with LC risk in the overall population. Third, we collected more detailed data. Fourth, we evaluated the quality of the eligible studies. Fifth, we assess the credibility of the significant association in the current and previous meta-analyses.

In summary, this meta-analysis strongly indicated that the *GSTM1* null genotype significantly increased LC risk in Japanese, *GSTM1* null genotype was significantly increased lung AC risk in Asians, *GSTT1* null genotype significantly increased LC risk in the Chinese population, and *GSTP1* IIe105Val polymorphisms have an association with increased LC risk. Another significant association should be interpreted with caution and it is essential that future analyses be based on sample sizes well-powered to identify these variants having modest effects on LC risk, especially the combined effects of gene-gene.

## Author contributions

The study was designed by Xiao-Feng He and Wei Wang. Chen Yang, Ling-Jun Xu, and Liang Song did the literature search, study quality assessment, and data extraction. Xiao-Feng He and Ling-Jun Xu performed the statistical analysis and drafted the tables and figures. Wen-Ping Zhang wrote the first draft of this analysis, and Xiao-Feng He and Wei Wang helped to finish the final version. All authors approved the conclusions of our study.

**Conceptualization:** Xiao-Feng He.

**Data curation:** Chen Yang, Ling-Jun Xu, Liang Song, Wen-Ping Zhang.

**Formal analysis:** Wei Wang, Liang Song, Xiao-Feng He, Wen-Ping Zhang.

**Funding acquisition:** No.

**Investigation:** Wei Wang, Ling-Jun Xu, Liang Song, Wen-Ping Zhang.

**Methodology:** Wei Wang, Liang Song, Wen-Ping Zhang, Xiao-Feng He.

**Resources:** Wei Wang, Chen Yang, Liang Song.

**Software:** Xiao-Feng He.

**Supervision:** Xiao-Feng He.

**Validation:** Xiao-Feng He.

**Visualization:** Xiao-Feng He.

**Writing – original draft:** Wen-Ping Zhang.

**Writing – review & editing:** Xiao-Feng He, Wei Wang.

## Supplementary Material

Supplemental Digital Content

## Supplementary Material

Supplemental Digital Content
