# Supplementary material for: Individual and combined effects of GSTM1, GSTT1, and GSTP1 polymorphisms on lung cancer risk: A meta-analysis and re-analysis of systematic meta-analyses
Source: Medicine (Baltimore). 2021 Jul 2;100(26):e26104. doi: 10.1097/MD.0000000000026104 (PMC8257913; doi:10.1097/MD.0000000000026104)
Supplement: Supplemental Digital Content [file medi-100-e26104-s001.pdf]

**Supplemental references**


[1] Seidegård J, Pero RW, Miller DG, et al. A glutathione transferase in human leukocytes as a marker for the susceptibility to lung cancer. Carcinogenesis 1986;7:751–3.

[2] Seidegård J, Pero RW, Markowitz MM, et al. Isoenzyme(s) of glutathione transferase (class Mu) as a marker for the susceptibility to lung cancer: a follow up study. Carcinogenesis 1990;11:33–6.

[3] Zhong S, Howie AF, Ketterer B, et al. Glutathione S-transferase mu locus: use of genotyping and phenotyping assays to assess association with lung cancer susceptibility. Carcinogenesis 1991;12:1533–7.

[4] Heckbert SR, Weiss NS, Hornung SK, et al. Glutathione S-transferase and epoxide hydrolase activity in human leukocytes in relation to risk of lung cancer and other smoking-related cancers. J Natl Cancer Inst 1992;84:414–22.

[5] Hayashi S, Watanabe J, Kawajiri K. High susceptibility to lung cancer analyzed in terms of combined genotypes of P450IA1 and Mu-class glutathione S-transferase genes. Jpn J Cancer Res 1992;83:866–70.

[6] Hirvonen A, Husgafvel-Pursiainen K, Anttila S, et al. The GSTM1 null genotype as a potential risk modifier for squamous cell carcinoma of the lung. Carcinogenesis 1993;14:1479–81.

[7] Brockmöller J, Kerb R, Drakoulis N, et al. Genotype and phenotype of glutathione S-transferase class mu isoenzymes mu and psi in lung cancer patients and controls. Cancer Res 1993;53:1004–11.

[8] Nakachi K, Imai K, Hayashi S, et al. Polymorphisms of the CYP1A1 and glutathione S-transferase genes associated with susceptibility to lung cancer in relation to cigarette dose in a Japanese population. Cancer Res 1993;53:2994–9.

[9] Nazar-Stewart V, Motulsky AG, Eaton DL, et al. The glutathione S-transferase mu polymorphism as a marker for susceptibility to lung carcinoma. Cancer Res 1993;53:2313–8.

[10] Katoh T. [The frequency of glutathione-S-transferase M1 (GSTM1) gene deletion in patients with lung and oral cancer]. Sangyo Igaku 1994;36:435–9.

[11] Alexandrie AK, Sundberg MI, Seidegård J, et al. Genetic susceptibility to lung cancer with special emphasis on CYP1A1 and GSTM1: a study on host factors in relation to age at onset, gender and histological cancer types. Carcinogenesis 1994;15:1785–90.

[12] Kihara M, Kihara M, Noda K. Lung cancer risk of GSTM1 null genotype is dependent on the extent of tobacco smoke exposure. Carcinogenesis 1994;15:415–8.

[13] London SJ, Daly AK, Cooper J, et al. Polymorphism of glutathione S-transferase M1 and lung cancer risk among African-Americans and Caucasians in Los Angeles County, California. J Natl Cancer Inst 1995;87:1246–53.

[14] Nakajima T, Elovaara E, Anttila S, et al. Expression and polymorphism of glutathione S-transferase in human lungs: risk factors in smoking-related lung cancer. Carcinogenesis 1995;16:707–11.

[15] Kihara M, Noda K, Kihara M. Distribution of GSTM1 null genotype in relation to gender, age and smoking status in Japanese lung cancer patients. Pharmacogenetics 1995;5:S74–9.

[16] Kihara M, Kihara M, Noda K. Risk of smoking for squamous and small cell carcinomas of the lung modulated by combinations of CYP1A1 and GSTM1 gene polymorphisms in a Japanese population. Carcinogenesis 1995;16:2331–6.

[17] Katoh T. [Application of molecular biology to occupational health field--the frequency of gene polymorphism of cytochrome P450 1A1 and glutathione S-transferase M1 in patients with lung, oral and urothelial cancer]. J UOEH 1995;17:271–8.

[18] To-Figueras J, Gene M, Gomez-Catalan J, et al. Glutathione-S-Transferase M1 and codon 72 p53 polymorphisms in a northwestern Mediterranean population and their relation to lung cancer susceptibility. Cancer Epidemiol Biomarkers Prev 1996;5:337–42.

[19] Moreira A, Martins G, Monteiro MJ, et al. Glutathione S-transferase mu polymorphism and susceptibility to lung cancer in the Portuguese population. Teratog Carcinog Mutagen 1996;16:269–74.

[20] Ge H, Lam WK, Lee J, et al. Analysis of L-myc and GSTM1 genotypes in Chinese non-small cell lung carcinoma patients. Lung Cancer 1996;15:355–66.

[21] Deakin M, Elder J, Hendrickse C, et al. Glutathione S-transferase GSTT1 genotypes and susceptibility to cancer: studies of interactions with GSTM1 in lung, oral, gastric and colorectal cancers. Carcinogenesis 1996;17:881–84.

[22] el-Zein R, Zwischenberger JB, Wood TG, et al. Combined genetic polymorphism and risk for development of lung cancer. Mutat Res 1997;381:189–200.

[23] Jourenkova N, Reinikanen M, Bouchardy C, et al. Effects of glutathione S-transferases GSTM1 and GSTT1 genotypes on lung cancer risk in smokers. Pharmacogenetics. 1997;7:515–8.

[24] To-Figueras J, Gené M, Gómez-Catalán J, et al. Glutathione S-transferase M1 (GSTM1) and T1 (GSTT1) polymorphisms and lung cancer risk among Northwestern Mediterraneans. Carcinogenesis 1997;18:1529–33.

[25] Harrison DJ, Cantlay AM, Rae F, et al. Frequency of glutathione S-transferase M1 deletion in smokers with emphysema and lung cancer. Hum Exp Toxicol 1997;16:356–60.

[26] Kelsey KT, Spitz MR, Zuo ZF, et al. Polymorphisms in the glutathione S-transferase class mu and theta genes interact and increase susceptibility to lung cancer in minority populations (Texas, United States). Cancer Causes Control 1997;8:554–9.

[27] Garcia-Closas M, Kelsey KT, Wiencke JK, et al. A case-control study of cytochrome P450 1A1, glutathione S-transferase M1, cigarette smoking and lung cancer susceptibility (Massachusetts, United States). Cancer Causes Control 1997;8:544–53.

[28] Ryberg D, Skaug V, Hewer A, et al. Genotypes of glutathione transferase M1 and P1 and their significance for lung DNA adduct levels and cancer risk. Carcinogenesis 1997;18:1285–9.

[29] Li WY, Lai BT, Zhan XP. The relationship between genetic polymorphism of metabolizing enzymes and the genetic susceptibility to lung cancer. Chin J Epidemiol 2004;25:1042–5.

[30] Sun GF, Shimojo N, Pi JB, et al. Gene deficiency of glutathione S-transferase mu isoform associated with susceptibility to lung cancer in a Chinese population. Cancer Lett 1997;113:169–72.
[31] Salagovic J, Kalina I, Stubna J, et al. Genetic polymorphism of glutathione S-transferases M1 and T1 as a risk factor in lung and bladder cancers. Neoplasma 1998;45:312–7.
[32] Hong YS, Chang JH, Kwon OJ, et al. Polymorphism of the CYP1A1 and glutathione-S-transferase gene in Korean lung cancer patients. Exp Mol Med 1998;30:192–8.
[33] Benhamou S, Reinikainen M, Bouchardy C, et al. Association between lung cancer and microsomal epoxide hydrolase genotypes. Cancer Res 1998;58:5291–3.
[34] Le Marchand L, Sivaraman L, Pierce L, et al. Associations of CYP1A1, GSTM1, and CYP2E1 polymorphisms with lung cancer suggest cell type specificities to tobacco carcinogens. Cancer Res 1998;58:4858–63.
[35] Nyberg F, Hou SM, Hemminki K, et al. Glutathione S-transferase mu1 and N-acetyltransferase 2 genetic polymorphisms and exposure to tobacco smoke in nonsmoking and smoking lung cancer patients and population controls. Cancer Epidemiol Biomarkers Prev 1998;7:875–83.
[36] Saarikoski ST, Voho A, Reinikainen M, et al. Combined effect of polymorphic GST genes on individual susceptibility to lung cancer. Int J Cancer 1998;77:516–21.
[37] Jourenkova-Mironova N, Wikman H, Bouchardy C, et al. Role of glutathione S-transferase GSTM1, GSTM3, GSTP1 and GSTT1 genotypes in modulating susceptibility to smoking-related lung cancer. Pharmacogenetics 1998;8:495–502.
[38] Stücker I, de Waziers I, Cenée S, et al. GSTM1, smoking and lung cancer: a case-control study. Int J Epidemiol 1999;28:829–35.
[39] Gao Y, Zhang Q. Polymorphisms of the GSTM1 and CYP2D6 genes associated with susceptibility to lung cancer in Chinese. Mutat Res 1999;444:441–9.
[40] To-Figueras J, Gené M, Gómez-Catalán J, et al. Genetic polymorphism of glutathione S-transferase P1 gene and lung cancer risk. Cancer Causes Control 1999;10:65–70.
[41] Persson I, Johansson I, Lou YC, et al. Genetic polymorphism of xenobiotic metabolizing enzymes among Chinese lung cancer patients. Int J Cancer 1999;81:325–9.
[42] Katoh T, Kaneko S, Takasawa S, et al. Human glutathione S-transferase P1 polymorphism and susceptibility to smoking related epithelial cancer; oral, lung, gastric, colorectal and urothelial cancer. Pharmacogenetics 1999;9:165–9.
[43] Kihara M, Kihara M, Noda K. Lung cancer risk of the GSTM1 null genotype is enhanced in the presence of the GSTP1 mutated genotype in male Japanese smokers. Cancer Lett 1999;137:53–60.
[44] Kiyohara C, Yamamura KI, Nakanishi Y, et al. Polymorphism in GSTM1, GSTT1, and GSTP1 and Susceptibility to Lung Cancer in a Japanese Population. Asian Pac J Cancer Prev 2000;1:293–8.
[45] Dresler CM, Fratelli C, Babb J, et al. Gender differences in genetic susceptibility for lung cancer. Lung Cancer 2000;30:153–60.
[46] London SJ, Yuan JM, Chung FL, et al. Isothiocyanates, glutathione S-transferase M1 and T1 polymorphisms, and lung-cancer risk: a prospective study of men in Shanghai, China. Lancet 2000;356:724–9.
[47] Ford JG, Li Y, O'Sullivan MM, et al. Glutathione S-transferase M1 polymorphism and lung cancer risk in African-Americans. Carcinogenesis 2000;21:1971–5.
[48] Stücker I, Jacquet M, de Waziers I, et al. Relation between inducibility of CYP1A1, GSTM1 and lung cancer in a French population. Pharmacogenetics 2000;10:617–27.
[49] Malats N, Camus-Radon AM, Nyberg F, et al. Lung cancer risk in nonsmokers and GSTM1 and GSTT1 genetic polymorphism. Cancer Epidemiol Biomarkers Prev 2000;9:827–33.
[50] Wang N, Wu YJ, Zhou XL, et al. Association between genetic polymorphism of metabolizing enzymes and DNA repairing enzymes and the susceptibility of lung cancer in Henan population. JOURNAL OF HYGIENE RESEARCH 2012;41:251–6.
[51] Hou SM, Ryberg D, Fält S, et al. GSTM1 and NAT2 polymorphisms in operable and non-operable lung cancer patients. Carcinogenesis 2000;21:49–54.
[52] Spitz MR, Duphorne CM, Detry MA, et al. Dietary intake of isothiocyanates: evidence of a joint effect with glutathione S-transferase polymorphisms in lung cancer risk. Cancer Epidemiol Biomarkers Prev 2000;9:1017–20.
[53] Cheng YW, Chen CY, Lin P, et al. DNA adduct level in lung tissue may act as a risk biomarker of lung cancer. Eur J Cancer 2000;36:1381–8.
[54] Belogubova EV, Togo AV, Kondratieva TV, et al. GSTM1 genotypes in elderly tumour-free smokers and non-smokers. Lung Cancer 2000;29:189–95.
[55] Lan Q, He X, Costa DJ, et al. Indoor coal combustion emissions, GSTM1 and GSTT1 genotypes, and lung cancer risk: a case-control study in Xuan Wei, China. Cancer Epidemiol Biomarkers Prev 2000;9:605–8.
[56] Liu G, Miller DP, Zhou W, et al. Differential association of the codon 72 p53 and GSTM1 polymorphisms on histological subtype of non-small cell lung carcinoma. Cancer Res 2001;61:8718–22.
[57] Risch A, Wikman H, Thiel S, et al. Glutathione-S-transferase M1, M3, T1 and P1 polymorphisms and susceptibility to non-small-cell lung cancer subtypes and hamartomas. Pharmacogenetics 2001;11:757–64.
[58] Quiñones L, Lucas D, Godoy J, et al. CYP1A1, CYP2E1 and GSTM1 genetic polymorphisms. The effect of single and combined genotypes on lung cancer susceptibility in Chilean people. Cancer Lett 2001;174:35–44.
[59] Zhao B, Seow A, Lee EJ, et al. Dietary isothiocyanates, glutathione S-transferase -M1, -T1 polymorphisms and lung cancer risk among Chinese women in Singapore. Cancer Epidemiol Biomarkers Prev 2001;10:1063–7.
[60] Chen SQ, Xue KX, Xu L, et al. Polymorphisms of the CYP1A1 and GSTM1 genes in relation to individual susceptibility to lung carcinoma in Chinese population. Mutat Res 2001;458:41–7.
[61] Hou SM, Fält S, Yang K, et al. Differential interactions between GSTM1 and NAT2 genotypes on aromatic DNA

adduct level and HPRT mutant frequency in lung cancer patients and population controls. Cancer Epidemiol Biomarkers Prev 2001;10:133–40.
[62] Benhamou S, Voho A, Bouchardy C, et al. Role of NAD(P)H:quinone oxidoreductase polymorphism at codon 187 in susceptibility to lung, laryngeal and oral/pharyngeal cancers. Biomarkers 2001;6:440–47.
[63] Gsur A, Haidinger G, Hollaus P, et al. Genetic polymorphisms of CYP1A1 and GSTM1 and lung cancer risk. Anticancer Res 2001;21:2237–42.
[64] Hou SM, Fält S, Nyberg F. Glutathione S-transferase T1-null genotype interacts synergistically with heavy smoking on lung cancer risk. Environ Mol Mutagen 2001;38:83–6.
[65] Qian BY, Han HW, Gu F, et al. Case-Control Study Genetic Polymorphism in CYP1A1 and GSTM1 and Smoking and Susceptibility to Lung Cancer. Chin J Clin Oncol 2006;33:500–2.
[66] Liu Q, Liu J, Song B, et al. Relationship between susceptibility to lung cancer and genetic polymorphism in CYP1A1 and GSTM1. Shandong Medical Journal 2008;48:32–4.
[67] Perera FP, Mooney LA, Stampfer M, et al. Associations between carcinogen-DNA damage, glutathione S-transferase genotypes, and risk of lung cancer in the prospective Physicians' Health Cohort Study. Carcinogenesis 2002;23:1641–6.
[68] Stücker I, Hirvonen A, de Waziers I, et al. Genetic polymorphisms of glutathione S-transferases as modulators of lung cancer susceptibility. Carcinogenesis 2002;23:1475–81.
[69] Lewis SJ, Cherry NM, Niven RM, et al. GSTM1, GSTT1 and GSTP1 polymorphisms and lung cancer risk. Cancer Lett 2002;180:165–71.
[70] Sunaga N, Kohno T, Yanagitani N, et al. Contribution of the NQO1 and GSTT1 polymorphisms to lung adenocarcinoma susceptibility. Cancer Epidemiol Biomarkers Prev 2002;11:730–8.
[71] Miller DP, Liu G, De Vivo I, et al. Combinations of the variant genotypes of GSTP1, GSTM1, and p53 are associated with an increased lung cancer risk. Cancer Res 2002;62:2819–23.
[72] Lu W, Xing D, Qi J, et al. Genetic polymorphism in myeloperoxidase but not GSTM1 is associated with risk of lung squamous cell carcinoma in a Chinese population. Int J Cancer 2002;102:275–9.
[73] Reszka E, Wasowicz W, Rydzynski K, et al. Glutathione S-transferase M1 and P1 metabolic polymorphism and lung cancer predisposition. Neoplasma 2003;50:357–62.
[74] Cajas-Salazar N, Sierra-Torres CH, Salama SA, et al. Combined effect of MPO, GSTM1 and GSTT1 polymorphisms on chromosome aberrations and lung cancer risk. Int J Hyg Environ Health 2003;206:473–83.
[75] Wang J, Deng Y, Cheng J, et al. GST genetic polymorphisms and lung adenocarcinoma susceptibility in a Chinese population. Cancer Lett 2003;201:185–93.
[76] Pinarbasi H, Silig Y, Cetinkaya O, et al. Strong association between the GSTM1-null genotype and lung cancer in a Turkish population. Cancer Genet Cytogenet 2003;146:125–9.
[77] Dialyna IA, Miyakis S, Georgatou N, et al. Genetic polymorphisms of CYP1A1, GSTM1 and GSTT1 genes and lung cancer risk. Oncol Rep 2003;10:1829–35.
[78] Tsai YY, McGlynn KA, Hu Y, et al. Genetic susceptibility and dietary patterns in lung cancer. Lung Cancer 2003;41:269–81.
[79] Kiyohara C, Wakai K, Mikami H, et al. Risk modification by CYP1A1 and GSTM1 polymorphisms in the association of environmental tobacco smoke and lung cancer: a case-control study in Japanese nonsmoking women. Int J Cancer 2003;107:139–44.
[80] Wang J, Deng Y, Li L, et al. Association of GSTM1, CYP1A1 and CYP2E1 genetic polymorphisms with susceptibility to lung adenocarcinoma: a case-control study in Chinese population. Cancer Sci 2003;94:448–52.
[81] Nazar-Stewart V, Vaughan TL, Stapleton P, et al. A population-based study of glutathione S-transferase M1, T1 and P1 genotypes and risk for lung cancer. Lung Cancer 2003;40:247–58.
[82] Hung RJ, Boffetta P, Brockmöller J, et al. CYP1A1 and GSTM1 genetic polymorphisms and lung cancer risk in Caucasian non-smokers: a pooled analysis. Carcinogenesis 2003;24:875–82.
[83] Taioli E, Gaspari L, Benhamou S, et al. Polymorphisms in CYP1A1, GSTM1, GSTT1 and lung cancer below the age of 45 years. Int J Epidemiol 2003;32:60–3.
[84] Lin P, Hsueh YM, Ko JL, et al. Analysis of NQO1, GSTP1, and MnSOD genetic polymorphisms on lung cancer risk in Taiwan. Lung Cancer 2003;40:123–9.
[85] Wang Y, Spitz MR, Schabath MB, et al. Association between glutathione S-transferase p1 polymorphisms and lung cancer risk in Caucasians: a case–control study. Lung Cancer 2003;40:25–32.
[86] Miller DP, De Vivo I, Neuberg D, et al. Association between self-reported environmental tobacco smoke exposure and lung cancer: modification by GSTP1 polymorphism. Int J Cancer 2003;104:758–63.
[87] Oztürk O, Isbir T, Yaylim I, et al. GST M1 and CYP1A1 gene polymorphism and daily fruit consumption in Turkish patients with non-small cell lung carcinomas. In Vivo 2003;17:625–32.
[88] Ruano-Ravina A, Figueiras A, Loidi L, et al. GSTM1 and GSTT1 polymorphisms, tobacco and risk of lung cancer: a case-control study from Galicia, Spain. Anticancer Res 2003;23:4333–7.
[89] Miller DP, Neuberg D, de Vivo I, et al. Smoking and the risk of lung cancer: susceptibility with GSTP1 polymorphisms. Epidemiology 2003;14:545–51.
[90] Wang LI, Giovannucci EL, Hunter D, et al. Dietary intake of Cruciferous vegetables, Glutathione S-transferase (GST) polymorphisms and lung cancer risk in a Caucasian population. Cancer Causes Control 2004;15:977–985.
[91] Vineis P, Veglia F, Anttila S, et al. CYP1A1, GSTM1 and GSTT1 polymorphisms and lung cancer: a pooled analysis of gene-gene interactions. Biomarkers 2004;9:298–305.
[92] Sobti RC, Sharma S, Joshi A, et al. Genetic polymorphism of the CYP1A1, CYP2E1, GSTM1 and GSTT1 genes and lung cancer susceptibility in a north indian population. Mol Cell Biochem 2004;266:1–9.
[93] Habalová V, Salagovic J, Kalina I, et al. Combined analysis of polymorphisms in glutathione S-transferase M1 and microsomal epoxide hydrolase in lung cancer patients. Neoplasma 2004;51:352–7.

[94] Yang XR, Wacholder S, Xu Z, et al. CYP1A1 and GSTM1 polymorphisms in relation to lung cancer risk in Chinese women. Cancer Lett 2004;214:197–204.
[95] Chan-Yeung M, Tan-Un KC, Ip MS, et al. Lung cancer susceptibility and polymorphisms of glutathione-S-transferase genes in Hong Kong. Lung Cancer 2004;45:155–60.
[96] Harms C, Salama SA, Sierra-Torres CH, et al. Polymorphisms in DNA repair genes, chromosome aberrations, and lung cancer. Environ Mol Mutagen 2004;44:74–82.
[97] Yang P, Bamlet WR, Ebbert JO, et al. Glutathione pathway genes and lung cancer risk in young and old populations. Carcinogenesis 2004;25:1935–44.
[98] Alexandrie AK, Nyberg F, Warholm M, et al. Influence of CYP1A1, GSTM1, GSTT1, and NQO1 genotypes and cumulative smoking dose on lung cancer risk in a Swedish population. Cancer Epidemiol Biomarkers Prev 2004;13:908–14.
[99] Belogubova EV, Togo AV, Karpova MB, et al. A novel approach for assessment of cancer predisposing roles of GSTM1 and GSTT1 genes: use of putatively cancer resistant elderly tumor-free smokers as the referents. Lung Cancer 2004;43:259–66.
[100] Schneider J, Bernges U, Philipp M, et al. GSTM1, GSTT1, and GSTP1 polymorphism and lung cancer risk in relation to tobacco smoking. Cancer Lett 2004;208:65–74.
[101] Sørensen M, Autrup H, Tjønneland A, et al. Glutathione S-transferase T1 null-genotype is associated with an increased risk of lung cancer. Int J Cancer 2004;110:219–24.
[102] Gallegos-Arreola MP, Gómez-Meda BC, Morgan-Villela G, et al. GSTT1 gene deletion is associated with lung cancer in Mexican patients. Dis Markers 2003–4;19:259–61.
[103] Lan Q, He X. Molecular epidemiological studies on the relationship between indoor coal burning and lung cancer in Xuan Wei, China. Toxicology 2004;198:301–5.
[104] Sreeja L, Syamala V, Hariharan S, et al. Possible risk modification by CYP1A1, GSTM1 and GSTT1 gene polymorphisms in lung cancer susceptibility in a South Indian population. J Hum Genet 2005;50:618–27.
[105] Chan EC, Lam SY, Fu KH, et al. Polymorphisms of the GSTM1, GSTP1, MPO, XRCC1, and NQO1 genes in Chinese patients with non-small cell lung cancers: relationship with aberrant promoter methylation of the CDKN2A and RARB genes. Cancer Genet Cytogenet 2005;162:10–20.
[106] Adonis M, Martínez V, Marín P, et al. Smoking habit and genetic factors associated with lung cancer in a population highly exposed to arsenic. Toxicol Lett 2005;159:32–7.
[107] Raimondi S, Boffetta P, Anttila S, et al. Metabolic gene polymorphisms and lung cancer risk in non-smokers. An update of the GSEC study. Mutat Res 2005;592:45–57.
[108] Skuladottir H, Autrup H, Autrup J, et al. Polymorphisms in genes involved in xenobiotic metabolism and lung cancer risk under the age of 60 years. A pooled study of lung cancer patients in Denmark and Norway. Lung Cancer 2005;48:187–99.
[109] Wenzlaff AS, Cote ML, Bock CH, et al. GSTM1, GSTT1 and GSTP1 polymorphisms, environmental tobacco smoke exposure and risk of lung cancer among never smokers: a population-based study. Carcinogenesis 2005;26:395–401.
[110] Cote ML, Kardia SL, Wenzlaff AS, et al. Combinations of glutathione S-transferase genotypes and risk of early-onset lung cancer in Caucasians and African Americans: a population-based study. Carcinogenesis 2005;26:811–9.
[111] Brennan P, Hsu CC, Moullan N, et al. Effect of cruciferous vegetables on lung cancer in patients stratified by genetic status: a mendelian randomisation approach. Lancet 2005;366:1558–60.
[112] Liang G, Pu Y, Yin L. Rapid detection of single nucleotide polymorphisms related with lung cancer susceptibility of Chinese population. Cancer Lett 2005;223:265–74.
[113] Lee KM, Kang D, Lee SJ, et al. Interactive effect of genetic polymorphism of glutathione S-transferase M1 and smoking on squamous cell lung cancer risk in Korea. Oncol Rep 2006;16:1035–9.
[114] Chen HC, Cao YF, Hu WX, et al. Genetic polymorphisms of phase II metabolic enzymes and lung cancer susceptibility in a population of Central South China. Dis Markers 2006;22:141–52.
[115] Pisani P, Srivatanakul P, Randerson-Moor J, et al. GSTM1 and CYP1A1 Polymorphisms, Tobacco, Air Pollution, and Lung Cancer: A Study in Rural Thailand. Cancer Epidemiol Biomarkers Prev 2006;15:667–74.
[116] Belogubova EV, Ulibina YM, Suvorova IK, et al. Combined CYP1A1/GSTM1 at-risk genotypes are overrepresented in squamous cell lung carcinoma patients but underrepresented in elderly tumor-free subjects. J Cancer Res Clin Oncol 2006;132:327–31.
[117] Larsen JE, Colosimo ML, Yang IA, et al. CYP1A1 Ile462Val and MPO G-463A interact to increase risk of adenocarcinoma but not squamous cell carcinoma of the lung. Carcinogenesis 2006;27:525–32.
[118] Miller DP, Asomaning K, Liu G, et al. An association between glutathione S-transferase P1 gene polymorphism and younger age at onset of lung carcinoma. Cancer 2006;107:1570–7.
[119] Reszka E, Wasowicz W, Gromadzinska J. Antioxidant defense markers modulated by glutathione S-transferase genetic polymorphism: results of lung cancer case-control study. Genes Nutr 2007;2:287–94.
[120] Osawa Y, Osawa KK, Miyaishi A, et al. NAT2 and CYP1A2 polymorphisms and lung cancer risk in relation to smoking status. Asian Pac J Cancer Prev 2007;8:103–8.
[121] Yang M, Choi Y, Hwangbo B, et al. Combined effects of genetic polymorphisms in six selected genes on lung cancer susceptibility. Lung Cancer 2007;57:135–42.
[122] Sørensen M, Raaschou-Nielsen O, Brasch-Andersen C, et al. Interactions between GSTM1, GSTT1 and GSTP1 polymorphisms and smoking and intake of fruit and vegetables in relation to lung cancer. Lung Cancer 2007;55:137–44.
[123] Zhang LZ, Wang X, Hao XZ, et al. Relationship between susceptibility to lung cancer and genetic polymorphism in P4501A1, GSTM1. Chin J Clin Oncol 2002;29:536–40.

[124] Loft S, Svoboda P, Kasai H, et al. Prospective study of urinary excretion of 7-methylguanine and the risk of lung cancer: Effect modification by mu class glutathione-S-transferases. Int J Cancer 2007;121:1579-1584.
[125] Shah PP, Singh AP, Singh M, et al. Association of functionally important polymorphisms in cytochrome P4501B1 with lung cancer. Mutat Res 2008;643:4–10.
[126] Chang FH, Hu TM, Wang G. Relationship between CYP1A1 and GSTM1 genetic polymorphisms and lung cancer susceptibility in population of Inner Mongolia. Chin J Lung Cancer 2006;9:413–7.
[127] Sobti RC, Kaur P, Kaur S, et al. Combined effect of GSTM1, GSTT1 and GSTP1 polymorphisms on histological subtypes of lung cancer. Biomarkers 2008;13:282–95.
[128] Honma HN, De Capitani EM, Perroud MW Jr, et al. Influence of p53 codon 72 exon 4, GSTM1, GSTT1 and GSTP1*B polymorphisms in lung cancer risk in a Brazilian population. Lung Cancer 2008;61:152–62.
[129] Dong CT, Yang Q, Wang MZ, et al. A study on the relationship between polymorphism of CYP1A1, lack of GSTM1 and susceptibility to lung cancer. J Environ Occup Med 2004;21:440–2.
[130] Zienolddiny S, Campa D, Lind H, et al. A comprehensive analysis of phase I and phase II metabolism gene polymorphisms and risk of non-small cell lung cancer in smokers. Carcinogenesis 2008;29:1164–9.
[131] Yoon KA, Kim JH, Gil HJ, et al. CYP1B1, CYP1A1, MPO, and GSTP1 polymorphisms and lung cancer risk in never-smoking Korean women. Lung Cancer 2008;60:40–6.
[132] Sreeja L, Syamala V, Hariharan S, et al. Glutathione S-transferase M1, T1 and P1 polymorphisms: susceptibility and outcome in lung cancer patients. J Exp Ther Oncol 2008;7:73–85.
[133] Matakova T, Sivonova M, Halasova E, et al. Gene polymorphisms of biotransforming enzymes (GSTs) and their association with lung cancer in the Slovakian population. Eur J Med Res 2009;14:275–9.
[134] Klinchid J, Chewaskulyoung B, Saeteng S, et al. Effect of combined genetic polymorphisms on lung cancer risk in northern Thai women. Cancer Genet Cytogenet 2009;195:143–9.
[135] Carpenter CL, Yu MC, London SJ. Dietary isothiocyanates, glutathione S-transferase M1 (GSTM1), and lung cancer risk in African Americans and Caucasians from Los Angeles County, California. Nutr Cancer 2009;61:492–9.
[136] Zupa A, Sgambato A, Bianchino G, et al. GSTM1 and NAT2 polymorphisms and colon, lung and bladder cancer risk: a case-control study. Anticancer Res 2009;29:1709–14.
[137] Lam TK, Ruczinski I, Helzlsouer K, et al. Copy number variants of GSTM1 and GSTT1 in relation to lung cancer risk in a prospective cohort study. Ann Epidemiol 2009;19:546–52.
[138] Cote ML, Yoo W, Wenzlaff AS, et al. Tobacco and estrogen metabolic polymorphisms and risk of non-small cell lung cancer in women. Carcinogenesis 2009;30:626–35.
[139] Kumar M, Agarwal SK, Goel SK. Lung cancer risk in north Indian population: role of genetic polymorphisms and smoking. Mol Cell Biochem 2009;322:73–9.
[140] Yadav DS, Devi TR, Ihsan R, et al. Polymorphisms of glutathione-S-transferase genes and the risk of aerodigestive tract cancers in the Northeast Indian population. Genet Test Mol Biomarkers 2010;14:715–23.
[141] Jin Y, Xu H, Zhang C, et al. Combined effects of cigarette smoking, gene polymorphisms and methylations of tumor suppressor genes on non small cell lung cancer: a hospital-based case-control study in China. BMC Cancer 2010;10:422.
[142] Gervasini G, San Jose C, Carrillo JA, et al. GST polymorphisms interact with dietary factors to modulate lung cancer risk: study in a high-incidence area. Nutr Cancer 2010;62:750–8.
[143] Timofeeva M, Kropp S, Sauter W, et al. Genetic polymorphisms of MPO, GSTT1, GSTM1, GSTP1, EPHX1 and NQO1 as risk factors of early-onset lung cancer. Int J Cancer 2010;127:1547–61.
[144] Cabral RE, Caldeira-de-Araujo A, Cabral-Neto JB, et al. Analysis of GSTM1 and GSTT1 polymorphisms in circulating plasma DNA of lung cancer patients. Mol Cell Biochem 2010;338:263–9.
[145] Altinisik J, Balta ZB, Aydin G, et al. Investigation of glutathione S-transferase M1 and T1 deletions in lung cancer. Mol Biol Rep 2010;37:263–7.
[146] Sreelekha TT, Rajesh M, Anil Kumar V, et al. CYP1A1m2 polymorphisms regulate estrogen and interleukin-6 in lung cancer. Mol Med Rep 2010;3:971–6.
[147] Lam TK, Rotunno M, Lubin JH, et al. Dietary quercetin, quercetin-gene interaction, metabolic gene expression in lung tissue and lung cancer risk. Carcinogenesis 2010;31:634–42.
[148] Tamaki Y, Arai T, Sugimura H, et al. Association between cancer risk and drug-metabolizing enzyme gene (CYP2A6, CYP2A13, CYP4B1, SULT1A1, GSTM1, and GSTT1) polymorphisms in cases of lung cancer in Japan. Drug Metab Pharmacokinet 2011;26:516–22.
[149] Kohno T, Kunitoh H, Mimaki S, et al. Contribution of the TP53, OGG1, CHRNA3, and HLA-DQA1 genes to the risk for lung squamous cell carcinoma. J Thorac Oncol 2011;6:813–17.
[150] Young RP, Hopkins RJ, Hay BA, et al. GSTM1 null genotype in COPD and lung cancer: evidence of a modifier or confounding effect? Appl Clin Genet 2011;4:137–44.
[151] Singh AP, Pant MC, Ruwali M, et al. Polymorphism in cytochrome P450 1A2 and their interaction with risk factors in determining risk of squamous cell lung carcinoma in men. Cancer Biomark 2010–1;8:351–9.
[152] Ihsan R, Chauhan PS, Mishra AK, et al. Multiple analytical approaches reveal distinct gene-environment interactions in smokers and non smokers in lung cancer. PLoS One 2011;6:e29431.
[153] Atinkaya C, Taspinar M, Sakiragaoglu O, et al. The effect of CYP1A1, GSTT1 and GSTM1 polymorphisms on the risk of lung cancer: a case-control study. Hum Exp Toxicol 2012;31:1074–80.
[154] Fowke JH, Gao YT, Chow WH, et al. Urinary isothiocyanate levels and lung cancer risk among non-smoking women: a prospective investigation. Lung Cancer 2011;73:18–24.
[155] Kiyohara C, Horiuchi T, Takayama K, et al. Genetic polymorphisms involved in carcinogen metabolism and DNA repair and lung cancer risk in a Japanese population. J Thorac Oncol 2012;7:954–62.
[156] Dzian A, Halasova E, Matakova T, et al. Lung adenocarcinoma and squamous cell carcinoma in association with

genetic polymorphisms of GSTs in Slovak population. Neoplasma 2012;59:160–7.
[157] Ada AO, Kunak SC, Hancer F, et al. Association between GSTM1, GSTT1, and GSTP1 polymorphisms and lung cancer risk in a Turkish population. Mol Biol Rep 2012;39(5):5985–93.
[158] Li W, Yue W, Zhang L, et al. Polymorphisms in GSTM1, CYP1A1, CYP2E1, and CYP2D6 are associated with susceptibility and chemotherapy response in non-small-cell lung cancer patients. Lung 2012;190:91–8.
[159] López-Cima MF, Alvarez-Avellón SM, Pascual T, et al. Genetic polymorphisms in CYP1A1, GSTM1, GSTP1 and GSTT1 metabolic genes and risk of lung cancer in Asturias. BMC Cancer 2012;12:433.
[160] Liu D, Wang F, Wang Q, et al. Association of glutathione S-transferase M1 polymorphisms and lung cancer risk in a Chinese population. Clin Chim Acta 2012;414:188–90.
[161] Vural B, Yakar F, Derin D, et al. Evaluation of glutathione S-transferase P1 polymorphisms (Ile105Val and Ala114Val) in patients with small cell lung cancer. Genet Test Mol Biomarkers 2012;16:701–6.
[162] Pliarchopoulou K, Voutsinas G, Papaxoinis G, et al. Correlation of CYP1A1, GSTP1 and GSTM1 gene polymorphisms and lung cancer risk among smokers. Oncol Lett 2012;3:1301–6.
[163] Shukla RK, Tilak AR, Kumar C, et al. Associations of CYP1A1, GSTM1 and GSTT1 polymorphisms with lung cancer susceptibility in a Northern Indian population. Asian Pac J Cancer Prev 2013;14:3345–9.
[164] Shukla RK, Kant S, Mittal B, et al. Comparative study of GST polymorphism in relation to age in COPD and lung cancer. Tuberk Toraks 2013;61:275–282.
[165] Piao JM, Shin MH, Kim HN, et al. Glutathione-S-transferase (GSTM1, GSTT1) null phenotypes and risk of lung cancer in a Korean population. Asian Pac J Cancer Prev 2013;14:7165–9.
[166] Ihsan R, Chauhan PS, Mishra AK, et al. Copy number polymorphism of glutathione-S-transferase genes (GSTM1 & GSTT1) in susceptibility to lung cancer in a high-risk population from north-east India. Indian J Med Res 2014;139:720–9.
[167] Zhang H, Wu X, Xiao Y, et al. Genetic polymorphisms of glutathione S-transferase M1 and T1, and evaluation of oxidative stress in patients with non-small cell lung cancer. Eur J Med Res 2014;19:67.
[168] Bag A, Bag N, Jeena LM, et al. Glutathione S-transferase T1 and myeloperoxidase -463 G>A genotypes in lung cancer patients of Kumaun region. J Nat Sci Biol Med 2014;5:293–6.
[169] Pan C, Zhu G, Yan Z, et al. Glutathione S-transferase T1 and M1 polymorphisms are associated with lung cancer risk in a gender-specific manner. Oncol Res Treat 2014;37:164–9.
[170] Jiang XY, Chang FH, Bai TY, et al. Susceptibility of Lung Cancer with Polymorphisms of CYP1A1, GSTM1, GSTM3, GSTT1 and GSTP1 Genotypes in the Population of Inner Mongolia Region. Asian Pac J Cancer Prev 2014;15:5207–14.
[171] Sharma N, Singh A, Singh N, et al. Genetic polymorphisms in GSTM1, GSTT1 and GSTP1 genes and risk of lung cancer in a North Indian population. Cancer Epidemiol 2015;39:947–55.
[172] Mota P, Silva HC, Soares MJ, et al. Genetic polymorphisms of phase I and phase II metabolic enzymes as modulators of lung cancer susceptibility. J Cancer Res Clin Oncol 2015;141:851–60.
[173] Wang Y, Ren BU, Zhang L, et al. Correlation between metabolic enzyme GSTP1 polymorphisms and susceptibility to lung cancer. Exp Ther Med 2015;10:1521–7.
[174] Peddireddy V, Badabagni SP, Gundimeda SD, et al. Association of CYP1A1, GSTM1 and GSTT1 gene polymorphisms with risk of non-small cell lung cancer in Andhra Pradesh region of South India. Eur J Med Res 2016;21:17.
[175] Masood N, Taseer B, Yasmin A. Association of GSTM1 and GSTT1 deletion with lung cancer development in Pakistani population. J Cancer Res Ther 2016;12:731–4.
[176] Girdhar Y, Singh N, Behera D, et al. Combinations of the Variant Genotypes of CYP1A1, GSTM1 and GSTT1 are Associated with an Increased Lung Cancer Risk in North Indian Population: a Case-Control Study. Pathol Oncol Res 2016;22:647–52.
[177] Ada AO, Bilgen S, Karacaoglan V, et al. Association between the TP53 and CYP2E1*5B gene polymorphisms and non-small cell lung cancer. Arh Hig Rada Toksikol 2016;67:311–6.
[178] Liu HX, Li J, Ye BG. Correlation between gene polymorphisms of CYP1A1, GSTP1, ERCC2, XRCC1, and XRCC3 and susceptibility to lung cancer. Genet Mol Res 2016;15.
[179] Wang Z, Feng F, Zhou X, et al. Development of diagnostic model of lung cancer based on multiple tumor markers and data mining. Oncotarget 2017;8:94793–804.
[180] Chen H, Yu ZC, Jin YT, et al. Influence of genetic polymorphism of CYP1A1 gene and GSTM1 gene on lung cancer. J shandong Med 2008;48:20–2.
[181] Minina VI, Soboleva OA, Glushkov AN, et al. Polymorphisms of GSTM1, GSTT1, GSTP1 genes and chromosomal aberrations in lung cancer patients. J Cancer Res Clin Oncol 2017;143:2235–43.
[182] He Q, Wang L, Zhang J, et al. CYP2E1 and GSTM1 gene polymorphisms, environmental factors, and the susceptibility to lung cancer. J Clin Lab Anal 2018:e22403.
[183] Lv XL, Chang FH, Yin Q, et al. Associations of genetic polymorphisms of GSTP1 and CYP1A1 with susceptibility to lung cancer. Chin J public Health 2013;29:169–72.
[184] Wang QM, Lu QF, Zhen HN, et al. Relationship between CYP2C9 and GSTM1 genetic polymorphism and lung cancer susceptibility. Canc Res Prev Treat 2006;33:8–10.
[185] Gao JR, Ren CL, Zhang Q. *CYP2D6* and *GSTM1* genetic polymorphism and lung cancer susceptibility. Chinese Journal of Oncology 1998;20:185–6.
[186] Shi Y, Zhou XW, Zhou YK, et al. Analysis of *CYP2E1*, *GSTM1* genetic polymorphisms in relation to human lung cancer and esophageal carcinoma. J Huazhong Univ Sci Tech [Health Sci] 2002;1:14–7.
[187] Ma DY, Du GB, Tan BX, et al. Study on genetic polymorphism of GSTM1 and GSTT1 related with susceptibility to lung cancer in the population of northern SiChuan of China. Journal of Cancer Control and Treatment 2013;26:136–9.

[188] Wang N, Wu YM, Wu YJ, et al. Study on *GSTM1* and *GSTT1* gene deletion with lung cancer genetic susceptibility. Journal of Hygiene Research 2004;33:586–8.
[189] Yao W, Wang N, Wu YJ, et al. Relationship between deletion of GSTM1, GSTT1 genes and susceptibility to lung cancer. Chin J Public Health 2006;22:1070–2.
[190] Gao JR, Zhang Q. Study on the relationship between *GSTM1* polymorphism and lung cancer susceptibility. Carcinogenesis Teratogenesis and Mutagenesis 1998;10:149–51.
[191] Liu AS, Guo LH, Wen Y, et al. Study the correlation of GSTM1 and CYP2E1 gene polymorphism and genetic susceptibility to non-small cell lung cancer in Shenzhen area. J Clin Transfus Lab Med 2017;19:260–4.
[192] Li DR, Zhou QH, Yuan TZ, et al. Study on the association between genetic polymorphism of CYP2E1, GSTM1 and susceptibility of lung cancer. Chin J lung cancer 2005;8:14–9.
[193] Zhang JK, Hu YL, Hu CF, et al. Study on genetic polymorphisms of *GSTM1* and *GSTT1* related with inherent susceptibility to lung cancer in women. China Public Health 2002;18:273–5.
[194] Ye WY, Chen Q, Chen SD. Study on relationship between GSTM1 polymorphism, diet factors and lung cancer. Chin J Public Health 2004;20:1120–1.
[195] Du GB, Ma DY, Tan BX, et al. Relationship between genetic polymorphism of GSTM1 gene and susceptibility to lung cancer in the population of northern Sichuan of China. Chinese Clinical Oncology 2011;16:602–5.
[196] Lu QF, Chen Y, Bai M. Association between susceptibility of lung cancer and genetic polymorphism of GSTM1. Journal of Clinical Pulmonary Medicine 2008;13:1444–5.
[197] Lu QG. Association between GSTM1polymorphism and susceptibility of lung cancer. Capital Medicine 2013;6:25–7.
[198] Lei FM, Li SF, Zhou WD, et al. A case-control study of the impact of glutathione S-transferase M1 polymorphism on the risk of lung cancer. Modern Preventive Medicine 2007;34:724–6.
[199] Han RL, Bai TY, Chang FH, et al. GSTM1 gene polymorphism and lung cancer susceptibility in Man population. Central South Pharmacy 2012;10:1–3.
[200] Yao ZG, Er Y, Wang HY. The interacted effects between Glutathione S-Transferase gene polymorphism and smoking in lung cancer. Chin J Med Guide 2012;14:185–8.
[201] Qiao GB, Sun CS, Li LS, et al. A case-control study on relationship between absence of *GSTM1* gene, smoking and susceptibility to non-small cell lung cancer. J Fourth Milmed Univ 2005;26:1008–10.
[202] Chen M, Chen SD, Wang BG. A case-control study of the impact of Glutathion S-Transferase M1 on the risk of lung cancer. China Cancer 2004;13:686–8.
[203] Cao YF, Chen HC. A case-control study of GSTP1 polymorphism and lung cancer susceptibility. Journal of Changzhi Medical College 2005;19:86–7.
[204] Tao J, Han ZG, Ma L, et al. Relationship between GSTP1 genetic polymorphism and susceptibility of lung cancer in Xinjiang Uygurs and Hans. Journal of Practical Oncology 2014;29:542–6.
[205] Yuan TZ, Zhou QH, Zhu W, et al. Relationship between genetic polymorphism of GSTT1 gene and inherent susceptibility to lung cancer in Han population in Sichuan, China. Chin J Lung cancers 2005;8:107–11.
[206] Liu JN, Zhou CZ, Po HM, et al. Relationship between GSTT1 genetic polymorphism and smoking and lung cancer susceptibility. Basic & Clinical Medicine 2012;32:1194–7.
[207] Bai TY, Chang FH, Wang MJ, et al. Relationship between CYP1A1 and GSTT1 polymorphisms and lung cancer susceptibility. Chin J Public Health 2011;27:723–5.
[208] Wang YS, Jin YT, Xue SL, et al. Study on the methylation of P16 gene and genetic polymorphism of GSTM1 gene related with susceptibility to non-small cell lung cancer. Modern Preventive Medicine 2007;34:1207–9, 12.
[209] Wang J, Li SB. Relationship between XRCC1 and GSTM1 polymorphisms and lung cancer susceptibility. Chinese Journal of Gerontology 2016;36:6163–4.
[210] Gu YF, Zhang SC, Lai BT, et al. Relationship between genetic polymorphism of metabolizing enzymes and lung cancer susceptibility. Chin J Lung Cancer 2004;7:112–7.
[211] Qu YH, Shi YB, Zhong LJ, et al. The genotypes of cytochrome P450 1A1 and GSTM1 in non-smoking female lung cancer. Tumor 1998;18:80–2.
[212] Li Y, Chen J, He X, et al. CYP1A1 and GSTM1 polymorphisms and susceptibility to lung cancer. Journal of Zhengzhou University (Medical Sciences) 2006;41:1061–4.
[213] Luo CL, Chen Q, Cao WF, et al. Combined analysis of polymorphisms of GSTM1 and mutations of p53 gene in the patients with lung cancer. Chin J Clin Oncol 2004;31:1218–20,24.
[214] He DX, Chan Y. The relationship of GSTT1 polymorphism and Chromosome 15 Aberration in lung cancer patients. China Journal of Cancer Prevention and Treatment 2006;33:308–10.
[215] Zhang TY, Wu YJ, Wang ZL, et al. Analysis of glulathione-s-transferase P1 polymorphism in patients with lung cancer. Journal of Zhengzhou University (Med Sci) 2006;41:448–51.
[216] Luo CL, Chen Q, Cao WF. Analysis on genetic polymorphisms of GSTM1 in lung cancer patients and their first-degree relatives. Chin J Public Health 2005;21:786–7.
[217] Zeng M, Chen SD, Xie CM, et al. Case-control study on relationship between lung cancer and its susceptibility marker. Chin J Public Health. 2005;21:771–4.
[218] Wang N, Zhou F, Wu YJ, et al. The relationship between genetic polymorphism of four metabolizing enzymes and susceptibility to lung cancer. Modern Preventive Medicine 2012;39:4545–7.
[219] Qi XS, Lv HM, Xia Y, et al. A primary case-control study on the relationship between genetic polymorphism of GSTM1 and lung cancer susceptibility to the people living in high radon-exposed area. Radiation Protection 2009;29:90–5.
[220] Qi XS, Lv HM, Xia Y, et al. A primary case-control study on the relationship between genetic polymorphism of GSTT1 and lung cancer susceptibility to the people living in high radon-exposed area. Chin Occuo Med 2008;35:361–3,7.

[221] Zhang HY, Wu XW, Xiao Y, et al. Genetic polymorphisms of Glutathione S-transferase M1 and T1 and evaluation of oxidative stress in patients with non-small cell lung cancer. Journal of China Medical University 2014;43:432–6.
[222] Zhang JK, Hu YL, Hu CF, et al. Study on genetic polymorphisms of *GSTM1* and *GSTT1* related with inherent susceptibility to lung cancer. Chinese Journal of Pathophysiology 2002;18:352–5.
[223] Li Y, Tang XY, Ma XT, et al. Glutathione S-transferase M1 polymorphisms and susceptibility to lung cancer. Journal of Medical Forum 2005;26:10–2.
[224] Xian XZ, Chen SD, Wang BG. The relationship between polymorphism of GSTM1 and susceptibility to lung cancer. Practical Preventive Medicine 2003;10:635–6.
[225] Lan Q, He XZ, Debra C, et al. Glutathione S-transferase GSTM1 and GSTT1 genotypes and susceptibility to lung cancer. Journal of Hygiene Research 1999;28:9–11.
[226] Zhang JQ, Long XY, Xiong GS, et al. The relationship between Glutathione S-transferase M1 and susceptibility to Xuanwei's lung cancer. Journal of Kunming Medical University 2011;6:56–8.
[227] Huang XH, Chen SD, Wang BG, et al. Study on the impact of GSTM1 polymorphism on the risk of histologic types of lung cancer: a case-control study. J of Pub Health and Prev Med 2004;15:24–6.
[228] Fan J, Gan LG, Liang XM. Relationship of GSTM1 and GSTT1 polymorphisms with lung cancer susceptibility in GuangXi Zhuang population. Journal of Oncology 2010;16:922–5.
[229] Wang DQ, Chen SD, Wang BG, et al. A case-control study on relationship between lung cancer and genetic polymorphisms of CYP1A1, CYP2E1, and GSTM1 in Han nationality, in Guangzhou area. China Cancer 2006;15:579–82.
[230] Wang MJ, Chang FH, Yin Q, et al. Relationship of GSTM1 polymorphism and lung cancer susceptibility in Mongolian population. Chin J Public Health 2009;25:1447–9.
[231] Ai C. The effect of GSTM1 gene polymorphism on lung cancer risk. Contemporary Medicine. 2011;17:50.
[232] Chen LJ, Sun HL, Xu YQ. Study on the allele frequency of GSTM1 gene in normal Han population in Wannan area and the relationship between GSTM1 genotype and the risk of lung cancer. Acta Academiae Medicinae Wannan 2003;22:13–6.
[233] Li Y, Chen J, Gao YX. Influence of smoking and the polymorphisms of CYP1A1 and GSTM1 on the susceptibility of lung cancer. Journal of Chinese Practical Diagnosis and Therapy 2011;25:140–3.
[234] Song B, Liu J, Huang HY, et al. Effects of metabolic enzyme CYP1A1 and GSTM1 gene polymorphisms and smoking factors on the occurrence of male lung squamous cell carcinoma. Basic & Clinical Medicine 2010;30:1193–6.
[235] Chen SD, Zhen M, Li ZB, et al. A case-control study on the impact of CYP2E1 and GSTM1 polymorphisms on the risk of lung cancer. Tumor 2004;24:99–103.
[236] Hu YL, Gao Y, Zhang Q. Genetic polymorphisms of CYP1A1 2D6 and GSTM1 related with susceptibility to lung cancer. Tumor 1998;18:269–71.
[237] Ye WY, Chen SD, Chen Q. Interaction between serum selenium level and polymorphism of GSTM1 in lung cancer. Acta Nutrimenta Sinica 2005;27:17–20.
[238] Zheng DJ, Feng H, Mei CR, et al. Association between GSTM1 genetic polymorphism and lung cancer risk by SYBR green I real-time PCR assay. Chin J Lung Cancer 2010;13:506–10.
[239] Chen SQ, Xu L, Ma GJ, et al. Identification of genetic polymorphism of CYP1A1 and GSTM1 in lung cancer patients by using allele-specific PCR and multiplex differential PCR. Carcinogenesis Teratogenesis and Mutagenesis 1999;11:119–21.
[240] Chan Y, Wang X, Wang XY, et al. A study of genetic polymorphism of GSTM1 gene in normal population and lung cancer population in Yunnan. Journal of Yunnan Normal University 2002;22:52–4.
[241] Xue KX, Xu L, Chen SQ, et al. Polymorphisms of the CYP1A1 and GSTM1 genes and their combined effects on individual susceptibility to lung cancer in a Chinese population. Chin J Med Genet 2001;18:125–7.
[242] Liang KC, Gan LG, Ruan LG, et al. Correlational research of the relationship between the genetic polymorphism of GSTM1 and GSTT1 in the Zhuang population and lung cancer. Acta Medicine Sinica 2012;25:813–7.
[243] Liang GY, Pu YP, Yin LH. Studies of the genes related to lung cancer susceptibility in Nanjing Han population, China. Yi Chuan 2004;26:584–8
[244] Chen CM. et al. Effects of CYP1A1 and GSTM1 gene polymorphisms and BPDE-DNA adducts on lung cancer. Chin J of Med Genet 2012;29:23–7.
[245] Jia HS. Relationship between the genetic polymorphism of GSTT1 gene, smoking and different histological lung cancer susceptibility [D]. Yanbian University 2010;p1–30.
[246] Xia Y et al. Polymorphisms of the cytochrome P450 and glutathiones-transferase genes associated with lung cancer susceptibility for the residents in high radonexposed area. Chin J of Radio Med and Prot 2008;28:327–32.
[247] Yin Q. A study on the relationship between genetic polymorphisms of GSTP1 and CYP1A1 and susceptibility to lung cancer[D]. Inner Mongolia Medical College 2009;p1-49.
[248] Harris MJ, Coggan M, Langton L et al. Polymorphism of the Pi class glutathione S-transferase in normal populations and cancer patients. Pharmacogenetics 1998;8:27–31.
[249] Nie LH, Wang SY, Hu YL. Genetic polymorphism of glutathione S-transferee PI gene and susceptibility to lung cancer. China Public Health 2002;18:791–2.
[250] Yue Z, Xu Q, Xu Y et al. GSTP1 gene polymorphism and susceptibility as well as chemotherapy sensitivity to non -small cell lung cancer. Chin J Cancer Prev Treat 2009;16:1441–4.
[251] Zhu XX, Hu CP, Gu QH. CYP1A1 polymorphisms, lack of glutathione S-transferase M1 (GSTM1), cooking oil fumes and lung cancer risk in non-smoking women. Zhonghua Jie He He Hu Xi Za Zhi 2010;33:817–22.
[252] Cao YF, Chen HC, Liu XF, et al. Study on the relationship between the genetic polymorphisms of GSTM1 and GSTT1 genes and lung cancer susceptibility in the population of Hunan province of China. Life Sci Res

2004;8:126–32.
[253] Reszka E, Wasowicz W, Gromadzinska J, et al. Evaluation of selenium, zinc and copper levels related to GST genetic polymorphism in lung cancer patients. Trace Elem Electrolytes 2005;22:23–32.
[254] Wang N, Zhuang D, Wu Y. Research on relationship between GSTM1 gene deletion and lung cancer genetic susceptibility. J Henan Univ Sci Tech 2005;23:7–8.
[255] Kihara M, Noda K, Kihara M. Distribution of GSTM1 null genotype in relation to gender, age, and smoking status in Japanese lung cancer patients. Pharmacogenetics 1995;5:S74–9.
[256] Kawajiri K, Watanabe J, Eguchi H, et al. Genetic polymorphisms of drug-metabolizing enzymes and lung cancer susceptibility. Pharmacogenetics 1995;5:S70–3.
[257] Cheng TJ, Christiani DC, Wiencke JK, et al. Comparison of sister chromatid exchange frequency in peripheral lymphocytes in lung cancer cases and controls. Mutat Res 1995;348:75–82.
[258] Tang DL, Rundle A, Warburton D, et al. Associations between both genetic and environmental biomarkers and lung cancer: Evidence of a greater risk of lung cancer in women smokers. Carcinogenesis 1998;19:1949–53.
[259] Butkiewicz D, Cole KJ, Phillips DH, et al. GSTM1, GSTP1, CYP1A1, and CYP2D6 polymorphisms in lung cancer patients from an environmentally polluted region of Poland: Correlation with lung DNA adduct levels. Eur J Cancer Prev 1999;8:315–23.
[260] Li DR. A case-control study on the association between genetic polymorphisms of CYP1A1, CYP2E1, GSTM1 and lung cancer susceptibility [D]. SiChuan University 2005;p1–136.
[261] Woodson K, Stewart C, Barrett M, et al. Effect of vitamin intervention on the relationship between GSTM1, smoking, and lung cancer risk among male smokers. Cancer Epidemiol Biomarkers Prev 1999;8:965–70.
[262] Chou YC, Wu MS, Wu CC, et al. Total Urinary Isothiocyanates, Glutathione S-Transferase M1 Genotypes, and Lung Cancer Risk: A Preliminary Nested Case-Control Study in Taiwan. J Med Sci 2005;25:21–6.
[263] Sgambato A, Campisi B, Zupa A, et al. Glutathione S-transferase (GST) polymorphisms as risk factors for cancer in a highly homogeneous population from southern Italy. Anticancer Res 22:3647–52.
[264] Qiao GB, Wu YL, Zhen WS, et al. A case-control study of GSTM1 deficiency and non-small-cell lung cancer. Acad J SUMS 2002;23:25–7.
[265] Barnholtz-Sloan JS, Chakraborty R, Sellers TA, et al. (2005) Examining population stratification via individual ancestry estimates versus self-reported race. Cancer Epidemiol Biomarkers Prev 14:1545–51.
[266] Wang YS. Polymorphism of GSTM1 and methylation of P16/PARK related with susceptibility to non-small cell lung cancer [D]. AnHui Medical University 2007;p1–80.
[267] To-Figueras J, Gene M, Gomez-Catalan J, et al. Lung cancer susceptibility in relation to combined polymorphisms of microsomal epoxide hydrolase and glutathione S-transferase P1. Cancer Lett 2001;173:155–62.
[268] Li SF. A case-control study on the associations between polymorphism of GSTM1, GSTT1 and susceptibility to breast cancer and lung cancer[D]. Sichuan University 2007;p1–42.
[269] Lan Q, He XZ, Costa DJ, et al. Glutathione S-transferase GSTM1 and GSTT1 genotypes and susceptibility to lung cancer. Journal of Hygiene Research 1991;28:9–11.
[270] He XZ. Indoor Coal Combustion Emissions, Lung Cancer and Susceptibility. Journal of Practical Oncology 2001;16:369–70.
[271] Zhou XL. Association of CYP1A1 and XRCC1 and GSTM1 polymorphisms with genetic susceptibility to lung cancer [D]. Zhengzhou University 2011;p1–47.
[272] Fan J. The study on the polymorphisms of GSTM1 and GSTT1 genes associated with susceptibility to lung cancer [D]. Guang Xi Medical University 2011;p1–59.
[273] Hou Y. Polymorphism of CYP1A1/GSTM1 and level of plasma Zinc/Copper related with non-small cell lung cancer [D]. AnHui Medical University 2008;p1–72.
[274] Hu XG. Relationship between genotype of GSTM1 gene and K-ras gene mutation in patients with lung cancer [D]. Zhengzhou University 2007;p1–62.
[275] Chen H. Polymorphism of GSTM1 and CYP1A1 gene and Methylation of P16 gene related with lung cancer [D]. AnHui Medical University 2009;p1–75.
[276] Liu JN. Study on the relationship between the genetic polymorphism of GSTT1 gene, smoking and lung cancer susceptibility [D]. Yanbian University 2009;p1–44.
[277] Liang GY. Studies on susceptibility genes of lung cancer in Chinese Han population and rapid detection techniques of single nucleotide polymorphisms [D]. Southeast University 2005;p1–124.
[278] Guo ZL. A case-control study on the association between genetic polymorphisms of metabolizing enzymes of CYP2D6, GSTPl and NAT2 and lung cancer susceptibility [D]. Sichuan University 2005;p1–137.
[279] Hua F. A case-control study on the association between genetic polymorphism of CYP2A13, MPO, GSTP1 and lung cancer susceptibility in Han-Chinese [D]. Tianjin Medical University 2010;p1–135.
[280] Sun GF, Pi JB, Zheng QM, et al. Study on the relationship between the GSTM1 null genotype and lung cancer. Chin J Tuberc Respir Dis 1995;18:167–9.
[281] Wang S. A study for the development of lung cancer and chronic obstructive pulmonary disease related to the GSTMl gene polymorphisms and oxidative stress [D]. Xi’an Fourth Mil Med Univ 2003;p1–135.
[282] Li Y. Cytochrome P450 lAl and glutathione S-transferase M1 polymorphisms and susceptibility to lung cancer [D]. Zhengzhou University 2004;p1–32 .
[283] Wang N. The relationship between the deletion of GSTM1, GSTT1 and susceptibility to lung cancer [D]. ZhengZhou University 2003;p1–71.
[284] Wang DQ, Chen SD, Wang BG, et al. Study on the impact of GST genetic polymorphisms, smoking on lung cancer susceptibility. China J Prev Med 2007;8:466–7.
[285] Gu YF, Zhang ZD, Zhang SC, et al. Combined effects of genetic polymorphisms in cytochrome P450s and GSTM1 on lung cancer susceptibility. Tuber Thor Tumor 2008;1:16–21.

[286] Zhang JK. Genetic polymorphisms of Glutathione S-transferase M1 and T1 gene related with the susceptibility to lung cancer [D]. Jinan University 2002;p1–79.
[287] Zheng DJ. A case-control study on the relationship between CYP1A1 、NAT2、GSTM1 polymorphisms and lung cancer susceptibility [D]. Tianjin Med Univ 2010;p1–44.
[288] Chen CM. Metabolic enzymes gene polymorphisms and BPDE-DNA adducts with lung tumorigenesis [D]. Zhejiang University 2012;p1–49.
[289] Bai TY. The study on the polymorphisms of GSTM1, GSTM3, GSTT1, GSTP1 genes and susceptibility to lung cancer in Mongolian population. Inner Mongolia Medical College 2011;p1–44.
[290] Du GB. A study of relationship between genetic polymorphism of GSTM1 and GSTT1 gene and susceptibility to lung cancer in the population of northern Sichuan of China [D]. North Sichuan Medical College 2011;p1–53.
[291] Zhu XX. CYP1A1 and GSTM1 polymorphisms and lung cancer risk in non-smoking women [D]. Central South University 2010;p1–48.

**Supplemental Table 1 General characteristics of studies included in pooling gene effects**

| First author/Year | Country | Race | Sample size | SC | Source of control | Type of control | Matching | Materi |
|---|---|---|---|---|---|---|---|---|
| Seidegård[1] 1986 | USA | Mixed | 66/78 | HB | HB | Cancer-free patients | ND | Periphe |
| Seidegård[2] 1990 | USA | Mixed | 125/114 | HB | HB | Cancer-free patients | ND | Periphe |
| Zhong[3] 1991 | UK | Mixed | 228/225 | ND | HB and volunteers | ND | ND | Periphe |
| Heckbert[4] 1992 | USA | Caucasian | 66/120 | CR | PB | Healthy populations | Age and sex | Blood |
| Hirvonen[6] 1993 | Finland | Mixed | 138/178 | HB | BD or volunteers | Cancer-free controls | ND | Periphe |
| Brockmöller[7] 1993 | Germany | Caucasian | 117/355 | HB | HB | Cancer-free patients | ND | Blood |
| Nakachi[8] 1993 | Japan | Asian | 85/170 | HB | PB | Cancer-free controls | Age and sex | Periphe |
| Nazar-Stewart[9] 1993 | USA | Mixed | 35/43 | HB | HB | Cancer-free patients | ND | Lung c |
| Katoh[10] 1994 | Japan | Asian | 53/91 | HB | Volunteers | Healthy controls | ND | Periphe |
| London [13] 1995 | USA | Caucasian | 184/465 | HB | PB | Cancer-free controls | Age | Periphe |
| London[13] 1995 | USA | African | 158/251 | HB | PB | Cancer-free controls | Age | Periphe |
| Nakajima[14] 1995 | Finland | Caucasian | 27/11 | HB | HB | Cancer-free patients | ND | Blood l |
| Kihara[15] 1995 | Japan | Asian | 447/469 | HB | PB | Healthy controls | Age and sex | Periphe |
| Katoh[17] 1995 | Japan | Asian | 33/88 | HB | Volunteers | Healthy controls | ND | Periphe |
| Kawajiri[256] 1995 | Japan | Asian | 327/358 | HB | PB | Healthy populations | Age and sex | Periphe |
| Cheng TJ[257] 1995 | USA | Mixed | 78/78 | HB | HB | ND | ND | Periphe |
| Moreira[19] 1996 | Portugal | Caucasian | 94/84 | HB | BD | Healthy controls | ND | Periphe |
| Ge H[20] 1996 | China | Asian | 89/53 | HB | HB | Cancer-free controls | ND | Norma |
| Deakin[21] 1996 | UK | Caucasian | 106/705 | HB | HB | Cancer-free patients | ND | Periphe |
| el-Zein[22] 1997 | USA | Mixed | 54/50 | HB | Volunteers | Healthy controls | Age and sex | Periphe |
| Harrison[25] 1997 | UK | Caucasian | 168/384 | ND | BD | Healthy controls | ND | Norma |
| Kelsey[26] 1997 | USA | Mixed | 60/146 | HB | Volunteers | Healthy controls | Age | Periphe |
| Kelsey[26] 1997 | USA | African | 108/132 | HB | Volunteers | Healthy controls | Age | Periphe |
| Garcia-Closas[27] 1997 | USA | Mixed | 416/446 | HB | HB | ND | ND | Blood |
| Ryberg[28] 1997 | Norway | Caucasian | 135/342 | HB | Volunteers | Healthy controls | Sex | Norma |
| Sun[30] 1997 | China | Asian | 207/364 | HB | HB | Out-patients | ND | Blood |
| Salagovic[31] 1998 | Slovakia | Caucasian | 117/248 | HB | PB | Healthy controls | ND | Blood |
| Hong[32] 1998 | Korea | Asian | 85/63 | HB | HB | ND | ND | Blood |
| Le Marchand[34] 1998 | USA | Mixed | 341/456 | PB | PB | Cancer-free controls | Age and sex | Periphe |
| Nyberg[35] 1998 | Sweden | Caucasian | 185/164 | HB | HB | Cancer-free controls | Age and sex | Blood |
| Saarikoski[36] 1998 | Finland | Caucasian | 208/294 | HB | BD | Healthy populations | ND | Periphe |
| Jourenkova-Mironova[37] 1998 | France | Caucasian | 150/172 | HB | HB | Cancer-free patients | ND | Periphe |
| Gao JR[185] 1998 | China | Asian | 46/70 | HB | HB | ND | ND | Periphe |
| Qu YH[211] 1998 | China | Asian | 182/179 | ND | ND | ND | Age and sex | Periphe |
| Hu YL[236] 1998 | China | Asian | 59/59 | HB | HB | Cancer-free patients | ND | Periphe |
| Harris MJ[248] 1998 | Australia | Mixed | 184/199 | HB | HB | ND | ND | Blood |
| Tang DL[258] 1998 | USA | Mixed | 136/115 | HB | HB | Cancer-free patients | ND | Periphe |
| Gao Y[39] 1999 | China | Asian | 59/132 | HB | HB | Cancer-free controls | Age and sex | Periphe |
| To-Figueras[40] 1999 | Spain | Caucasian | 164/332 | HB | Volunteers | Healthy controls | ND | Periphe |
| Persson[41] 1999 | China | Asian | 76/122 | ND | ND | Healthy controls | ND | Blood |
| Katoh[42] 1999 | Japan | Asian | 47/122 | HB | Volunteers | Healthy controls | ND | Periphe |
| Kihara[43] 1999 | Japan | Asian | 382/257 | HB | HB | Healthy controls | Sex | Periphe |
| Butkiewicz[259] 1999 | Poland | Caucasian | 165/325 | HB | Volunteers | Healthy controls | ND | Lung n |
| Woodson[261] 1999 | Finland | Caucasian | 319/333 | PB | PB | ND | Age and sex | Periphe |
| Kiyohara[44] 2000 | Japan | Asian | 86/88 | HB | Volunteers | Healthy controls | Sex | Periphe |
| Dresler[45] 2000 | USA | Mixed | 180/163 | HB | HB and Community | Healthy controls | ND | The wh |
| London[46] 2000 | China | Asian | 232/710 | PB | PB | Cancer-free controls | Age and sex | Periphe |
| Ford[47] 2000 | USA | African | 117/120 | HB + CR | HB | Cancer-free patients | ND | Periphe |
| Malats[49] 2000 | Multiple | Caucasian | 122/121 | HB | HB | Healthy controls | ND | The wh |
| Hou[51] 2000 | Norway | Caucasian | 282/375 | HB | Volunteers | Healthy controls | Sex | Norma |
| Spitz[52] 2000 | USA | Caucasian | 503/465 | HB | HB | ND | Age and sex | Blood |
| Cheng YW[53] 2000 | China | Asian | 73/33 | HB | HB | Cancer-free controls | ND | Norma |
| Lan[55] 2000 | China | Asian | 122/122 | HB | PB | ND | Age and sex | Buccal |
| Liu[56] 2001 | USA | Mixed | 1,168/1,256 | HB | HB | ND | ND | Periphe |
| Risch[57] 2001 | Germany | Caucasian | 389/353 | HB | HB | Cancer-free patients | ND | Venous |
| Quiñones[58] 2001 | Chile | Mixed | 61/122 | HB | ND | Healthy controls | ND | Periphe |
| Zhao[59] 2001 | Singapore | Asian | 233/187 | HB | HB | Cancer-free patients | Age | Periphe |
| Chen SQ[60] 2001 | China | Asian | 106/106 | HB | HB | Healthy controls | Age and sex | Whole |
| Hou[61] 2001 | Sweden | Caucasian | 185/164 | HB | PB | Healthy controls | Age and sex | Blood |
| Gsur[63] 2001 | Austria | Caucasian | 134/134 | HB | HB | Cancer-free patients | Age | Blood |

| | | | | | | | | |
|---|---|---|---|---|---|---|---|---|
| Hou[64] 2001 | Sweden | Caucasian | 184/162 | HB | PB | Healthy controls | Age and sex | Blood |
| Perera[67] 2002 | USA | Caucasian | 89/173 | PB | PB | Cancer-free controls | Age and sex | Blood |
| Stücker[68] 2002 | France | Caucasian | 251/268 | HB | HB | Cancer-free controls | Age and sex | Blood |
| Lewis[69] 2002 | UK | Caucasian | 94/165 | HB | HB | Cancer-free patients | ND | Blood |
| Sunaga[70] 2002 | Japan | Asian | 198/152 | HB | HB | Cancer-free patients | ND | Whole |
| Miller[71] 2002 | USA | Caucasian | 767/927 | HB | HB | Cancer-free controls | ND | Blood |
| Lu[[72] 2002 | China | Asian | 314/320 | HB | PB | Healthy controls | Age and sex | Periph |
| Zhang LZ[123] 2002 | China | Asian | 65/60 | HB | HB | Cancer-free patients | ND | Periph |
| Shi Y[186]] 2002 | China | Asian | 120/120 | HB | HB | Cancer-free patients | ND | Whole |
| Chan Y[240] 2002 | China | Asian | 56/99 | HB | Volunteers | Healthy controls | ND | Periph |
| Nie LH[249] 2002 | China | Asian | 158/168 | HB | HB | Healthy controls | Age and sex | Periph |
| Sgambato[263] 2002 | Italy | Caucasian | 13/100 | HB | HB | Healthy controls | ND | Periph |
| Zhang JK[286] 2002 | China | Asian | 161/165 | HB | Volunteers | Healthy controls | Age and sex | Periph |
| Cajas-Salazar[74] 2003 | USA | Caucasian | 110/119 | HB | HB | Cancer-free patients | Age and sex | Periph |
| Wang J[75] 2003 | China | Asian | 112/119 | HB | HB | Cancer-free controls | Age and sex | Periph |
| Pinarbasi[76] 2003 | Turkey | Caucasian | 101/206 | HB | HB | Healthy controls | ND | Blood |
| Dialyna[77] 2003 | Greece | Caucasian | 122/178 | HB | HB | Healthy controls | ND | Whole |
| Tsai[78] 2003 | USA | Mixed | 235/94 | HB | HB | Healthy controls | ND | Whole |
| Kiyohara[79] 2003 | Japan | Asian | 158/259 | HB | HB | Cancer-free patients | Age and sex | Whole |
| Wang J[80] 2003 | China | Asian | 164/181 | HB | HB | Cancer-free controls | Age and sex | Periph |
| Nazar-Stewart[81] 2003 | USA | Mixed | 274/500 | PB | PB | ND | Age and sex | Whole |
| Hung[82] 2003 | Multiple | Caucasian | 284/1,433 | HB + PB | HB + PB | Cancer-free controls | ND | Blood |
| Taioli[83] 2003 | Multiple | Caucasian | 261/1,452 | HB + PB | HB + PB | Cancer-free controls | ND | Blood |
| Lin[84] 2003 | China | Asian | 198/332 | HB | HB | Cancer-free controls | ND | Blood |
| Wang Y[85] 2003 | USA | Caucasian | 362/419 | HB | HB | Healthy controls | Age and sex | Whole |
| Oztürk[87] 2003 | Turkey | Caucasian | 55/60 | HB | HB | Healthy controls | Age | Blood |
| Ruano-Ravina[88] 2003 | Spain | Caucasian | 132/187 | HB | HB | Cancer-free patients | ND | Blood |
| Chen LJ[232] 2003 | China | Asian | 38/99 | HB | ND | Healthy controls | ND | Periph |
| Wang S[281] 2003 | China | Asian | 97/71 | HB | HB | Cancer-free patients | ND | Tumor |
| Wang LI[90] 2004 | USA | Caucasian | 716/939 | HB | HB | ND | ND | Periph |
| Vineis[91] 2004 | Multiple | Caucasian | 1,967/2,719 | HB + PB | HB + PB | Cancer-free controls | ND | Blood |
| Sobti[92] 2004 | India | Indian | 100/76 | HB | PB | Healthy controls | ND | Periph |
| Habalová[93] 2004 | Slovak | Caucasian | 121/150 | HB | HB | Healthy controls | ND | Periph |
| Yang XR[94] 2004 | China | Asian | 186/139 | PB | PB | ND | Age and sex | Whole |
| Chan-Yeung[95] 2004 | China | Asian | 229/197 | HB | PB | Healthy controls | ND | Venous |
| Yang P[97] 2004 | USA | Mixed | 237/234 | HB | PB | Cancer-free controls | ND | Blood |
| Alexandrie[98] 2004 | Sweden | Caucasian | 524/530 | HB | HB and volunteers | Cancer-free controls | ND | Blood |
| Belogubova[99] 2004 | Russia | Caucasian | 167/663 | ND | HB | Cancer-free controls | ND | Periph |
| Schneider[100] 2004 | Germany | Caucasian | 446/622 | HB | HB | Cancer-free controls | ND | Whole |
| Gallegos[102] 2003-2004 | Mexico | Mixed | 52/178 | HB | ND | Healthy controls | ND | Periph |
| Dong CT[129] 2004 | China | Asian | 82/91 | HB | HB | Cancer-free patients | ND | Periph |
| Wang N[188] 2004 | China | Asian | 77/107 | HB | HB | Healthy controls | ND | Periph |
| Ye WY[194]] 2004 | China | Asian | 58/62 | HB | HB | Cancer-free patients | ND | Venous |
| Gu YF[210] 2004 | China | Asian | 180/224 | HB | HB and volunteers | Cancer-free controls | ND | Periph |
| Luo CL[213] 2004 | China | Asian | 63/47 | HB | HB | Cancer-free patients | ND | Norma |
| Huang XH[227] 2004 | China | Asian | 91/138 | HB | HB | Cancer-free patients | Age and sex | Venous |
| Cao YF[252] 2004 | China | Asian | 104/205 | HB | HB | ND | Age and sex | Venous |
| Li WY[29] 2004 | China | Asian | 217/200 | HB | HB | Cancer-free patients | ND | Venous |
| Li Y[282] 2004 | China | Asian | 103/138 | HB | Volunteers | Healthy controls | Age and sex | Periph |
| Sreeja[104] 2005 | India | Indian | 146/146 | HB | HB | Healthy controls | ND | Whole |
| Chan EC[105] 2005 | China | Asian | 75/162 | ND | HB | Cancer-free controls | Age and sex | Norma |
| Adonis[106] 2005 | Chile | Mixed | 57/103 | HB | HB | Healthy controls | ND | ND |
| Raimondi[107] 2005 | Multiple | Caucasian | 531/1,981 | HB + PB | HB + PB | Cancer-free controls | ND | Blood |
| Raimondi[107] 2005 | Multiple | Asian | 93/210 | HB + PB | HB + PB | Cancer-free controls | ND | Blood |
| Skuladottir[108] 2005 | Denmark | Caucasian | 320/618 | HB + PB | HB + PB | Healthy controls | Age and sex | Periph |
| Wenzlaff[109] 2005 | USA | Caucasian | 135/151 | PB | PB | ND | Age and sex | Blood, |
| Wenzlaff[109] 2005 | USA | African | 31/30 | PB | PB | ND | Age and sex | Blood, |
| Cote[110] 2005 | USA | Caucasian | 230/287 | PB | PB | ND | Age and sex | Blood, |
| Cote[110] 2005 | USA | African | 90/119 | PB | PB | ND | Age and sex | Blood, |
| Brennan[111] 2005 | Multiple | Caucasian | 2,141/2,168 | HB + PB | HB + PB | ND | Age and sex | Blood |
| Li DR[192] 2005 | China | Asian | 99/66 | HB | HB | Cancer-free patients | ND | Venous |
| Qiao GB[201] 2005 | China | Asian | 213/199 | HB | HB | Healthy controls | ND | Periph |

| | | | | | | | | |
|---|---|---|---|---|---|---|---|---|
| Cao YF[203] 2005 | China | Asian | 97/197 | HB | HB | ND | Age and sex | Venous |
| Yuan TZ[205]] 2005 | China | Asian | 150/152 | HB | HB | Healthy controls | ND | Periph |
| Luo CL[216] 2005 | China | Asian | 63/139 | HB | HB | Cancer-free controls | ND | Periph |
| Barnholtz-Sloan[265] 2005 | USA | Caucasian | 192/363 | PB | PB | ND | Age and sex | Periph |
| Barnholtz-Sloan[265] 2005 | USA | African | 60/131 | PB | PB | ND | Age and sex | Periph |
| Guo ZL[278] 2005 | China | Asian | 150/152 | HB | HB | Healthy controls | ND | Periph |
| Chou YC[262] 2005 | China | Asian | 30/60 | PB | PB | Cancer-free controls | Age and sex | Periph |
| Li DR[260] 2005 | China | Asian | 150/152 | HB | HB | Cancer-free controls | ND | Periph |
| Liang GY[277] 2005 | China | Asian | 227/227 | HB | HB | Cancer-free patients | Age and sex | Periph |
| Lee[113] 2006 | Korea | Asian | 171/196 | HB | HB | Cancer-free patients | Sex | Periph |
| Chen HC[114] 2006 | China | Asian | 97/197 | HB | HB | Healthy controls | Age and sex | Periph |
| Pisani[115] 2006 | Thailand | Asian | 166/283 | HB | HB+PB | Cancer-free controls | Age and sex | Venous |
| Larsen[117] 2006 | Australia | Caucasian | 1,103/627 | HB | HB | Cancer-free controls | ND | Fresh l |
| Miller[118] 2006 | USA | Caucasian | 1,921/1,343 | HB | HB | Cancer-free controls | ND | Blood |
| Qian BY[65] 2006 | China | Asian | 108/108 | HB | ND | Healthy controls | Sex | ND |
| Chang FH[126] 2006 | China | Asian | 163/163 | HB | HB | Cancer-free patients | Sex | Blood |
| Wang QM[184] 2006 | China | Asian | 56/42 | HB | HB | ND | ND | Venous |
| He DX[214] 2006 | China | Asian | 61/46 | HB | BD | Healthy controls | ND | Periph |
| Zhang TY[215] 2006 | China | Asian | 121/121 | HB | HB | Healthy controls | Age and sex | Periph |
| Wang DQ[229] 2006 | China | Asian | 91/91 | HB | HB | Cancer-free patients | Age and sex | Periph |
| Reszka[119] 2007 | Poland | Caucasian | 404/410 | HB | HB | Cancer-free patients | Age and sex | Whole |
| Osawa[120] 2007 | Japan | Asian | 113/121 | HB | HB | Cancer-free controls | ND | Whole |
| Yang[121] 2007 | Korea | Asian | 318/353 | HB | HB | Cancer-free controls | ND | Periph |
| Sørensen[122] 2007 | Denmark | Caucasian | 430/767 | PB | PB | Cancer-free controls | ND | Periph |
| Loft[124] 2007 | Denmark | Caucasian | 251/261 | PB | PB | Cancer-free controls | Age and sex | Periph |
| Wang YS[266] 2007 | China | Asian | 94/94 | HB | HB | Cancer-free controls | Age and sex | Periph |
| Li SF[268] 2007 | China | Asian | 42/103 | HB | HB | Cancer-free controls | Age and sex | Periph |
| Hu XG[274] 2007 | China | Asian | 112/104 | HB | HB | Cancer-free controls | Age and sex | Norma |
| Shah[125] 2008 | India | Indian | 200/200 | HB | ND | Healthy controls | ND | Blood |
| Sobti[127] 2008 | India | Indian | 151/151 | HB | HB | Cancer-free patients | ND | Periph |
| Honma[128] 2008 | Brazil | Mixed | 200/264 | HB | HB | ND | ND | Periph |
| Zienolddiny[130] 2008 | Norway | Caucasian | 365/413 | HB | PB | Healthy controls | Age and sex | Periph |
| Yoon[131] 2008 | Korea | Asian | 213/213 | HB | HB | Healthy controls | Age and sex | Periph |
| Sreeja[132] 2008 | India | Indian | 211/211 | HB | HB | Cancer-free controls | ND | Blood |
| Liu Q[66] 2008 | China | Asian | 110/125 | HB | HB | Healthy controls | ND | Periph |
| Qi XS[220] 2008 | China | Asian | 53/72 | HB | HB | Cancer-free patients | ND | Blood |
| Xia Y[246] 2008 | China | Asian | 58/116 | HB | HB | Cancer-free patients | Age and sex | Periph |
| Hou Y[273] 2008 | China | Asian | 77/77 | HB | HB | Cancer-free controls | Age and sex | Periph |
| Matakova[133] 2009 | Slovakia | Caucasian | 160/220 | HB | Volunteers | Healthy controls | Age and sex | Blood |
| Klinchid[134] 2009 | Thailand | Asian | 91/82 | HB | Volunteers | Cancer-free controls | ND | Venous |
| Carpenter[135] 2009 | USA | Caucasian | 311/622 | HB | PB | Cancer-free controls | Age and sex | Blood |
| Zupa[136] 2009 | Italy | Caucasian | 75/121 | HB | HB | Cancer-free patients | ND | Whole |
| Lam[137] 2009 | USA | Mixed | 143/447 | PB | PB | Cancer-free controls | Age and sex | Periph |
| Cote[138] 2009 | USA | Caucasian | 389/406 | PB | PB | Cancer-free controls | Age and sex | buccal |
| Cote[138] 2009 | USA | African | 115/121 | PB | PB | Cancer-free controls | Age and sex | buccal |
| Kumar[139] 2009 | India | Indian | 93/253 | HB | ND | Healthy controls | ND | Blood |
| Qi XS[219] 2009 | China | Asian | 53/72 | HB | HB | Cancer-free patients | ND | Blood |
| Wang MJ[230] 2009 | China | Asian | 304/635 | HB | HB | Healthy controls | ND | Blood |
| Yue Z[250] 2009 | China | Asian | 102/102 | HB | HB | Healthy controls | Age and sex | Blood |
| Chen H[275] 2009 | China | Asian | 158/455 | HB | HB | Healthy controls | ND | Periph |
| Liu JN[276] 2009 | China | Asian | 100/135 | HB | HB | Healthy controls | ND | Periph |
| Yin Q[247] 2009 | China | Asian | 116/216 | HB | HB | Healthy controls | ND | Periph |
| Yadav[140] 2010 | India | Indian | 101/221 | HB | HB | Healthy controls | Age and sex | Periph |
| Jin[141] 2010 | China | Asian | 150/150 | HB | HB | Cancer-free controls | Age and sex | Periph |
| Gervasini[142] 2010 | Spain | Caucasian | 103/247 | HB | HB | Cancer-free controls | Age and sex | Periph |
| Timofeeva[143] 2010 | Germany | Caucasian | 638/1,300 | HB | PB | Cancer-free controls | Age and sex | Periph |
| Cabral[144] 2010 | Brazil | Caucasian | 42/75 | HB | Volunteers | Healthy controls | ND | Blood ( |
| Cabral[144] 2010 | Brazil | African | 22/12 | HB | Volunteers | Healthy controls | ND | Blood ( |
| Altinisik[145] 2010 | Turkey | Caucasian | 75/55 | ND | ND | Cancer-free controls | ND | Periph |
| Sreelekha[146] 2010 | India | Indian | 86/60 | HB | ND | Healthy controls | Age and sex | Periph |
| Lam[147] 2010 | Italy | Caucasian | 1,822/1,991 | HB | PB | Cancer-free controls | Age and sex | Periph |
| Song B[234] 2010 | China | Asian | 125/125 | HB | HB | ND | ND | Periph |

| | | | | | | | | |
|---|---|---|---|---|---|---|---|---|
| Hua F[279] 2010 | China | Asian | 266/307 | HB | HB | Healthy controls | Age and sex | Periph[illegible] |
| Zheng DJ[287] 2010 | China | Asian | 266/307 | HB | HB | Healthy controls | Age and sex | Periph[illegible] |
| Zhu XX[291] 2010 | China | Asian | 160/160 | HB | HB | Cancer-free controls | Sex | Periph[illegible] |
| Tamaki[148] 2011 | Japan | Asian | 192/203 | HB | HB | Cancer-free controls | Age and sex | Periph[illegible] |
| Kohno[149] 2011 | Japan | Asian | 377/325 | HB | HB | Cancer-free controls | ND | Whole |
| Young[150] 2011 | New Zealand | Caucasian | 454/1,157 | HB | HB | Cancer-free controls | ND | Whole |
| Singh[151] 2010-2011 | India | Indian | 200/200 | HB | ND | Healthy controls | Age and sex | Whole |
| Ihsan[152] 2011 | India | Indian | 188/290 | HB | Volunteers | Healthy controls | Age and sex | Blood |
| Atinkaya[153] 2012 | Turkey | Caucasian | 128/122 | HB | HB | Healthy controls | ND | Periph[illegible] |
| Fowke[154] 2011 | China | Asian | 209/787 | PB | PB | Cancer-free controls | Sex | Blood |
| Bai TY[207] 2011 | China | Asian | 106/250 | HB | HB | Cancer-free patients | Age and sex | Periph[illegible] |
| Zhang JQ[226] 2011 | China | Asian | 50/50 | HB | ND | Healthy controls | Age and sex | Periph[illegible] |
| Ai C[231] 2011 | China | Asian | 50/50 | HB | HB | ND | ND | Periph[illegible] |
| Zhou XL[271] 2011 | China | Asian | 208/209 | HB | HB | Cancer-free controls | ND | Periph[illegible] |
| Bai TY[289] 2011 | China | Asian | 128/214 | HB | HB | Healthy controls | ND | Periph[illegible] |
| Du GB[290] 2011 | China | Asian | 125/125 | HB | HB | Cancer-free patients | ND | Periph[illegible] |
| Kiyohara[155] 2012 | Japan | Asian | 462/379 | HB | HB | Cancer-free patients | ND | Blood |
| Dzian[156] 2012 | Slovak | Caucasian | 230/290 | HB | HB + Volunteers | Cancer-free controls | ND | Periph[illegible] |
| Ada[157] 2012 | Turkey | Caucasian | 213/231 | HB | HB | Cancer-free controls | ND | Whole |
| López-Cima[159] 2012 | Australia | Caucasian | 789/789 | HB | HB | Cancer-free patients | Age and sex | Periph[illegible] |
| Liu D[160] 2012 | China | Asian | 360/360 | HB | PB | Healthy controls | Age and sex | Whole |
| Vural[161] 2012 | Turkey | Caucasian | 89/108 | HB | HB | Cancer-free patients | ND | Whole |
| Pliarchopoulou[162] 2012 | Greece | Caucasian | 100/125 | HB | HB | Healthy controls | ND | Blood |
| Wang N[50] 2012 | China | Asian | 209/256 | HB | HB | Healthy controls | Age and sex | Venous |
| Yao ZG[200] 2012 | China | Asian | 150/150 | HB | HB | Healthy controls | ND | Venous |
| Liang KC[242] 2012 | China | Asian | 68/70 | HB | HB | Cancer-free patients | ND | Periph[illegible] |
| Chen CM[288] 2012 | China | Asian | 200/200 | HB | HB | Cancer-free controls | ND | Periph[illegible] |
| Shukla[163] 2013 | India | Indian | 218/238 | HB | HB | Healthy controls | ND | Periph[illegible] |
| Shukla[164] 2013 | India | Indian | 218/204 | HB | HB | COPD | ND | Periph[illegible] |
| Piao[165] 2013 | Korea | Asian | 3,933/1,699 | HB | PB | ND | ND | Periph[illegible] |
| Lu QG[197] 2013 | China | Asian | 91/138 | HB | HB | ND | ND | Periph[illegible] |
| Zhang HY[167] 2014 | China | Asian | 110/100 | HB | Volunteers | Healthy controls | ND | Blood |
| Bag[168] 2014 | India | Indian | 26/33 | HB | Volunteers | Healthy controls | ND | Periph[illegible] |
| Pan[169] 2014 | China | Asian | 623/623 | HB | PB | Healthy controls | Age and sex | Periph[illegible] |
| Jiang XY[170] 2014 | China | Asian | 322/456 | HB | PB | Healthy controls | Age and sex | Whole |
| Tao J[204] 2014 | China | Asian | 160/160 | HB | HB | ND | ND | Periph[illegible] |
| Sharma[171] 2015 | India | Indian | 270/270 | HB | HB | Cancer-free controls | Age and sex | Periph[illegible] |
| Mota[172] 2015 | Portugal | Caucasian | 200/247 | HB | HB | Cancer-free controls | ND | Periph[illegible] |
| Peddireddy[174] 2016 | India | Indian | 246/250 | HB | PB | Healthy controls | Age and sex | Whole |
| Masood[175] 2016 | Pakistan | Indian | 252/270 | ND | ND | Cancer-free controls | Age and sex | ND |
| Girdhar[176] 2016 | India | Indian | 320/320 | HB | HB | Cancer-free controls | Age and sex | Periph[illegible] |
| Liu[178] 2016 | China | Asian | 308/253 | HB | Volunteers | Healthy controls | ND | Blood |
| Wang J[209] 2016 | China | Asian | 150/150 | HB | HB | ND | ND | Venous |
| Wang[179] 2017 | China | Asian | 200/200 | HB | HB | Healthy controls | ND | Periph[illegible] |
| Minina[181] 2017 | Russia | Caucasian | 353/300 | HB | HB | Healthy controls | ND | Periph[illegible] |
| Liu AS[191] 2017 | China | Asian | 71/71 | HB | HB | Cancer-free controls | ND | Venous |
| He[182] 2018 | China | Asian | 313/330 | HB | PB | Healthy controls | Age and sex | Blood |

HB = hospital-based, PB = population-based, CR = cancer registry, ND = not described, BD = Blood donors

**Supplemental Table 2 Quality assessment by included studies of meta-analysis of *GSTM1* and *GSTT1* polymorphisms**

| First author/Year | Source of case | Source of control | Ascertainment of cancer | Ascertainment of control | Matching | Genotyping examination | Specimens u determining gen |
|---|---|---|---|---|---|---|---|
| Seidegård[1] 1986 | 2 | 1 | 2 | 1 | 0 | 0 | 1 |
| Seidegård[2] 1990 | 2 | 1 | 2 | 1 | 0 | 0 | 1 |
| Zhong[3] 1991 | 0 | 1.5 | 0 | 0 | 0 | 0 | 1 |
| Heckbert[4] 1992 | 3 | 3 | 0 | 1 | 2 | 1 | 1 |
| Hirvonen[6] 1993 | 2 | 2 | 2 | 1 | 0 | 0 | 1 |
| Brockmöller[7] 1993 | 2 | 1 | 2 | 2 | 0 | 1 | 1 |
| Nakachi[8] 1993 | 2 | 3 | 2 | 1 | 2 | 0 | 1 |
| Nazar-Stewart[9] 1993 | 2 | 1 | 2 | 2 | 0 | 0 | 0 |
| Katoh[10] 1994 | 2 | 2 | 2 | 1 | 0 | 0 | 1 |
| Alexandrie[11] 1994 | 2 | 2 | 2 | 1 | 0 | 0 | 1 |
| London[13] 1995 | 2 | 3 | 2 | 1 | 1 | 0 | 1 |
| London[13] 1995 | 2 | 3 | 1 | 1 | 1 | 0 | 1 |
| Nakajima[14] 1995 | 2 | 1 | 2 | 2 | 0 | 0 | 1 |
| Kihara[15] 1995 | 2 | 3 | 2 | 2 | 2 | 0 | 1 |
| Katoh[17] 1995 | 2 | 2 | 2 | 1 | 0 | 0 | 1 |
| Kawajiri[256] 1995 | 2 | 3 | 2 | 1 | 2 | 0 | 1 |
| Cheng TJ[257] 1995 | 2 | 1 | 1 | 0 | 0 | 1 | 1 |
| Moreira[19] 1996 | 2 | 2 | 2 | 1 | 0 | 0 | 1 |
| Ge[20] 1996 | 2 | 1 | 2 | 1 | 0 | 0 | 1 |
| Deakin[21] 1996 | 2 | 1 | 0 | 1 | 0 | 0 | 1 |
| el-Zein[22] 1997 | 2 | 2 | 1 | 1 | 2 | 0 | 1 |
| Harrison[25] 1997 | 0 | 2 | 0 | 1 | 0 | 0 | 1 |
| Kelsey[26] 1997 | 2 | 2 | 2 | 1 | 1 | 2 | 1 |
| Kelsey[26] 1997 | 2 | 2 | 2 | 1 | 1 | 2 | 1 |
| Garcia-Closas[27] 1997 | 2 | 1 | 2 | 1 | 0 | 0 | 1 |
| Ryberg[28] 1997 | 2 | 2 | 2 | 1 | 1 | 0 | 0 |
| Sun[30] 1997 | 2 | 1 | 2 | 1 | 0 | 0 | 1 |
| Salagovic[31]] 1998 | 2 | 3 | 0 | 1 | 0 | 0 | 1 |
| Hong[32] 1998 | 2 | 1 | 2 | 1 | 0 | 0 | 1 |
| Le Marchand[34] 1998 | 3 | 3 | 2 | 2 | 2 | 1 | 1 |
| Nyberg[35] 1998 | 2 | 1 | 2 | 1 | 2 | 0 | 1 |
| Saarikoski[36] 1998 | 2 | 2 | 2 | 1 | 0 | 0 | 1 |
| Jourenkova-Mironova[37] 1998 | 2 | 1 | 2 | 1 | 0 | 0 | 1 |
| Gao JR[185] 1998 | 2 | 1 | 2 | 0 | 0 | 0 | 1 |
| Qu YH[211] 1998 | 0 | 0 | 0 | 1 | 2 | 0 | 1 |
| Hu YL[236] 1998 | 2 | 1 | 2 | 1 | 0 | 0 | 1 |
| Tang DL[258] 1998 | 2 | 1 | 2 | 1 | 0 | 0 | 1 |
| Gao[39] 1999 | 2 | 1 | 2 | 1 | 2 | 0 | 1 |
| To-Figueras[40] 1999 | 2 | 2 | 2 | 2 | 0 | 0 | 1 |
| Persson[41] 1999 | 0 | 0 | 1 | 1 | 0 | 0 | 1 |
| Kihara[43] 1999 | 2 | 1 | 2 | 2 | 1 | 0 | 1 |
| Butkiewicz[259] 1999 | 2 | 1 | 2 | 1 | 0 | 1 | 1 |
| Woodson[261] 1999 | 3 | 3 | 2 | 0 | 2 | 1 | 1 |
| Kiyohara[44] 2000 | 2 | 2 | 2 | 1 | 1 | 0 | 1 |
| Dresler[45] 2000 | 2 | 1 | 2 | 1 | 0 | 0 | 1 |
| London[46] 2000 | 3 | 3 | 2 | 1 | 2 | 0 | 1 |
| Ford[47] 2000 | 2 | 1 | 2 | 1 | 0 | 0 | 1 |
| Malats[49] 2000 | 2 | 1 | 2 | 1 | 0 | 1 | 1 |
| Hou[51] 2000 | 2 | 2 | 2 | 1 | 1 | 0 | 0 |
| Spitz[52] 2000 | 2 | 1 | 2 | 1 | 2 | 0 | 1 |
| Cheng[53] 2000 | 2 | 1 | 2 | 1 | 0 | 0 | 1 |
| Lan[55] 2000 | 2 | 3 | 1 | 0 | 2 | 2 | 1 |
| Liu[56] 2001 | 2 | 1 | 2 | 0 | 0 | 1 | 1 |
| Risch[57] 2001 | 2 | 1 | 2 | 1 | 0 | 0 | 1 |
| Quiñones[58] 2001 | 2 | 0 | 2 | 1 | 0 | 0 | 1 |
| Zhao[59] 2001 | 2 | 1 | 2 | 1 | 1 | 1 | 1 |
| Chen[60] 2001 | 2 | 0 | 2 | 1 | 2 | 0 | 1 |
| Hou[61] 2001 | 2 | 3 | 0 | 1 | 2 | 0 | 1 |
| Gsur[63] 2001 | 2 | 1 | 2 | 1 | 1 | 0 | 1 |
| Hou[64] 2001 | 2 | 3 | 0 | 1 | 2 | 0 | 1 |
| Perera[67] 2002 | 3 | 3 | 2 | 1 | 2 | 1 | 1 |
| Stücker[68] 2002 | 2 | 1 | 2 | 1 | 2 | 0 | 1 |
| Lewis[69] 2002 | 2 | 1 | 2 | 1 | 0 | 0 | 1 |
| Sunaga[70] 2002 | 2 | 1 | 2 | 1 | 0 | 0 | 1 |

| | | | | | | | |
|---|---|---|---|---|---|---|---|
| Miller[71] 2002 | 2 | 1 | 2 | 1 | 0 | 1 | 1 |
| Lu[72] 2002 | 2 | 3 | 2 | 1 | 2 | 2 | 1 |
| Zhang LZ[123] 2002 | 2 | 1 | 2 | 1 | 0 | 0 | 1 |
| Shi Y[186] 2002 | 2 | 1 | 2 | 1 | 0 | 0 | 1 |
| Chan Y[240] 2002 | 2 | 2 | 0 | 1 | 0 | 0 | 1 |
| Sgambato[263] 2002 | 2 | 1 | 0 | 1 | 0 | 0 | 1 |
| Zhang JK[286] 2002 | 2 | 2 | 2 | 2 | 2 | 0 | 1 |
| Cajas-Salazar[74] 2003 | 2 | 1 | 2 | 1 | 2 | 1 | 1 |
| Wang[75] 2003 | 2 | 1 | 2 | 1 | 2 | 0 | 1 |
| Pinarbasi[76] 2003 | 2 | 1 | 2 | 1 | 0 | 0 | 1 |
| Dialyna[77] 2003 | 2 | 1 | 2 | 1 | 0 | 0 | 1 |
| Tsai[78] 2003 | 2 | 1 | 1 | 1 | 0 | 0 | 1 |
| Kiyohara[79] 2003 | 2 | 1 | 2 | 1 | 2 | 0 | 1 |
| Wang[80] 2003 | 2 | 1 | 2 | 1 | 2 | 0 | 1 |
| Nazar-Stewart[81] 2003 | 3 | 3 | 2 | 1 | 2 | 1 | 1 |
| Hung[82] 2003 | 2 | 2 | 2 | 1 | 0 | 0 | 1 |
| Taioli[83] 2003 | 2 | 2 | 2 | 1 | 0 | 0 | 1 |
| Oztürk[87] 2003 | 2 | 1 | 2 | 1 | 1 | 1 | 1 |
| Ruano-Ravina[88] 2003 | 2 | 1 | 1 | 1 | 0 | 0 | 1 |
| Chen LJ[232]] 2003 | 2 | 0 | 2 | 1 | 0 | 0 | 1 |
| Wang S[281] 2003 | 2 | 1 | 2 | 1 | 0 | 0 | 0 |
| Wang[90] 2004 | 2 | 1 | 2 | 0 | 0 | 0 | 1 |
| Vineis[91] 2004 | 2 | 2 | 2 | 1 | 0 | 0 | 1 |
| Sobti[92] 2004 | 2 | 3 | 0 | 1 | 0 | 0 | 1 |
| Habalová[93]] 2004 | 2 | 1 | 2 | 1 | 0 | 1 | 1 |
| Yang[94]] 2004 | 3 | 3 | 2 | 1 | 2 | 0 | 1 |
| Chan-Yeung[95]] 2004 | 2 | 3 | 2 | 1 | 0 | 0 | 1 |
| Yang[97] 2004 | 2 | 3 | 2 | 1 | 0 | 0 | 1 |
| Alexandrie[98] 2004 | 2 | 2 | 2 | 1 | 0 | 0 | 1 |
| Belogubova[99] 2004 | 0 | 1 | 0 | 1 | 0 | 2 | 1 |
| Schneider[100] 2004 | 2 | 1 | 2 | 2 | 0 | 0 | 1 |
| Gallegos-Arreola[102] 2003-2004 | 2 | 0 | 2 | 1 | 0 | 0 | 1 |
| Dong CT[129] 2004 | 2 | 1 | 2 | 1 | 0 | 0 | 1 |
| Wang N[188] 2004 | 2 | 1 | 2 | 1 | 0 | 0 | 1 |
| Ye WY[194] 2004 | 2 | 1 | 2 | 1 | 0 | 0 | 1 |
| Gu YF[210] 2004 | 2 | 1 | 2 | 1 | 0 | 0 | 1 |
| Luo CL[213]] 2004 | 2 | 1 | 2 | 1 | 0 | 0 | 1 |
| Huang XH[227] 2004 | 2 | 1 | 2 | 1 | 2 | 0 | 1 |
| Cao YF[252] 2004 | 2 | 1 | 2 | 1 | 2 | 0 | 1 |
| Li WY[29] 2004 | 2 | 1 | 1 | 1 | 0 | 0 | 1 |
| Li Y[282] 2004 | 2 | 2 | 2 | 1 | 2 | 2 | 1 |
| Sreeja[104] 2005 | 2 | 1 | 2 | 1 | 0 | 1 | 1 |
| Chan[105] 2005 | 0 | 1 | 0 | 1 | 2 | 0 | 1 |
| Adonis[106] 2005 | 2 | 1 | 2 | 1 | 0 | 0 | 0 |
| Raimondi[107] 2005 | 2 | 2 | 2 | 1 | 0 | 0 | 1 |
| Raimondi[107] 2005 | 2 | 2 | 2 | 1 | 0 | 0 | 1 |
| Skuladottir[108] 2005 | 2 | 2 | 2 | 1 | 2 | 0 | 1 |
| Wenzlaff[109] 2005 | 3 | 3 | 2 | 0 | 2 | 1 | 0 |
| Wenzlaff[109] 2005 | 3 | 3 | 2 | 0 | 2 | 1 | 0 |
| Cote[110] 2005 | 3 | 3 | 2 | 0 | 2 | 1 | 0 |
| Cote[110] 2005 | 3 | 3 | 2 | 0 | 2 | 1 | 0 |
| Brennan[111] 2005 | 2 | 2 | 0 | 0 | 2 | 0 | 1 |
| Li DR[192] 2005 | 2 | 1 | 2 | 1 | 0 | 0 | 1 |
| Qiao GB[201]] 2005 | 2 | 1 | 2 | 1 | 0 | 0 | 1 |
| Yuan TZ[205] 2005 | 2 | 1 | 2 | 1 | 0 | 0 | 1 |
| Luo CL[216] 2005 | 2 | 1 | 2 | 1 | 0 | 0 | 1 |
| Barnholtz-Sloan[265] 2005 | 3 | 3 | 1 | 0 | 2 | 0 | 1 |
| Barnholtz-Sloan[265] 2005 | 3 | 3 | 1 | 0 | 2 | 0 | 1 |
| Chou YC[262]] 2005 | 3 | 3 | 0 | 1 | 2 | 0 | 1 |
| Li DR[260] 2005 | 2 | 1 | 2 | 1 | 0 | 0 | 1 |
| Liang GY[277]] 2005 | 2 | 1 | 2 | 1 | 2 | 1 | 1 |
| Lee[113] 2006 | 2 | 1 | 2 | 2 | 1 | 1 | 1 |
| Chen[114] 2006 | 2 | 1 | 2 | 2 | 2 | 1 | 1 |
| Pisani[115] 2006 | 2 | 2 | 2 | 2 | 2 | 0 | 1 |
| Larsen[117] 2006 | 2 | 1 | 2 | 2 | 0 | 0 | 1 |
| Qian[65] 2006 | 2 | 0 | 2 | 1 | 1 | 0 | 0 |
| Chang FH[126] 2006 | 2 | 1 | 2 | 1 | 1 | 0 | 1 |
| Wang QM[184] 2006 | 2 | 1 | 2 | 1 | 0 | 0 | 1 |

| | | | | | | |
|---|---|---|---|---|---|---|
| He DX[214] 2006 | 2 | 2 | 0 | 1 | 0 | 0 | 1 |
| Wang DQ[229] 2006 | 2 | 1 | 2 | 1 | 2 | 0 | 1 |
| Reszka[119] 2007 | 2 | 1 | 2 | 1 | 2 | 1 | 1 |
| Osawa[120] 2007 | 2 | 1 | 2 | 1 | 0 | 0 | 1 |
| Yang[121]] 2007 | 2 | 1 | 2 | 1 | 0 | 0 | 1 |
| Sørensen[122] 2007 | 3 | 3 | 2 | 1 | 0 | 0 | 1 |
| Loft[124] 2007 | 3 | 3 | 2 | 1 | 2 | 1 | 1 |
| Wang YS[266] 2007 | 2 | 1 | 2 | 1 | 2 | 1 | 1 |
| Li SF[268] 2007 | 2 | 1 | 0 | 1 | 2 | 0 | 1 |
| Hu XG[274] 2007 | 2 | 1 | 2 | 2 | 2 | 0 | 1 |
| Shah[125] 2008 | 2 | 0 | 2 | 1 | 0 | 1 | 1 |
| Sobti[127]] 2008 | 2 | 1 | 2 | 2 | 0 | 0 | 1 |
| Honma[128] 2008 | 2 | 2 | 2 | 1 | 0 | 0 | 1 |
| Zienolddiny[130] 2008 | 2 | 3 | 2 | 1 | 2 | 0 | 1 |
| Sreeja[132] 2008 | 2 | 1 | 2 | 1 | 0 | 0 | 1 |
| Liu Q[66] 2008 | 2 | 1 | 2 | 1 | 0 | 0 | 1 |
| Qi XS[220] 2008 | 2 | 1 | 2 | 1 | 0 | 0 | 1 |
| Xia Y[246] 2008 | 2 | 1 | 2 | 1 | 2 | 0 | 1 |
| Hou Y[273] 2008 | 2 | 1 | 2 | 1 | 2 | 0 | 1 |
| Matakova[133] 2009 | 2 | 2 | 2 | 1 | 2 | 2 | 1 |
| Klinchid[134] 2009 | 2 | 2 | 2 | 2 | 0 | 0 | 1 |
| Carpenter[135] 2009 | 2 | 3 | 2 | 1 | 2 | 0 | 1 |
| Zupa[136]] 2009 | 2 | 1 | 2 | 1 | 0 | 0 | 1 |
| Lam[137] 2009 | 3 | 3 | 2 | 1 | 2 | 0 | 1 |
| Cote[138] 2009 | 3 | 3 | 2 | 1 | 2 | 1 | 1 |
| Cote[138] 2009 | 3 | 3 | 2 | 1 | 2 | 1 | 1 |
| Kumar[139] 2009 | 2 | 0 | 0 | 1 | 0 | 0 | 1 |
| Qi XS[219] 2009 | 2 | 1 | 2 | 1 | 0 | 0 | 1 |
| Wang MJ[230] 2009 | 2 | 1 | 2 | 1 | 0 | 0 | 1 |
| Chen H[275] 2009 | 2 | 1 | 2 | 1 | 0 | 1 | 1 |
| Liu JN[276] 2009 | 2 | 1 | 2 | 1 | 0 | 0 | 1 |
| Yadav[140] 2010 | 2 | 1 | 2 | 1 | 2 | 1 | 1 |
| Jin[141] 2010 | 2 | 1 | 2 | 2 | 2 | 0 | 1 |
| Gervasini[142]] 2010 | 2 | 1 | 2 | 2 | 2 | 0 | 1 |
| Timofeeva[143] 2010 | 2 | 3 | 0 | 2 | 2 | 1 | 1 |
| Cabral[144] 2010 | 2 | 2 | 2 | 1 | 0 | 0 | 1 |
| Cabral[144] 2010 | 2 | 2 | 2 | 1 | 0 | 0 | 1 |
| Altinisik[145] 2010 | 0 | 0 | 2 | 1 | 0 | 0 | 1 |
| Sreelekha[146] 2010 | 2 | 0 | 0 | 1 | 2 | 0 | 1 |
| Song B[234] 2010 | 2 | 1 | 2 | 0 | 0 | 0 | 1 |
| Zheng DJ[287] 2010 | 2 | 1 | 2 | 1 | 2 | 1 | 1 |
| Zhu XX[291] 2010 | 2 | 1 | 2 | 1 | 1 | 1 | 1 |
| Tamaki[148] 2011 | 2 | 1 | 2 | 1 | 2 | 0 | 1 |
| Kohno[149] 2011 | 2 | 1 | 2 | 2 | 0 | 0 | 1 |
| Young[150] 2011 | 2 | 1 | 2 | 2 | 0 | 0 | 1 |
| Singh[151] 2010-2011 | 2 | 0 | 2 | 1 | 2 | 1 | 1 |
| Ihsan[152] 2011 | 2 | 2 | 2 | 1 | 2 | 1 | 1 |
| Atinkaya[153] 2012 | 2 | 1 | 2 | 2 | 0 | 1 | 1 |
| Fowke[154] 2011 | 3 | 3 | 2 | 2 | 1 | 1 | 1 |
| Bai TY[207] 2011 | 2 | 1 | 2 | 1 | 2 | 0 | 1 |
| Zhang JQ[226] 2011 | 2 | 0 | 2 | 1 | 2 | 0 | 1 |
| Ai C[231] 2011 | 2 | 1 | 2 | 0 | 0 | 0 | 1 |
| Zhou XL[271] 2011 | 2 | 1 | 2 | 1 | 0 | 2 | 1 |
| Bai TY[289] 2011 | 2 | 1 | 2 | 1 | 0 | 0 | 1 |
| Du GB[290] 2011 | 2 | 1 | 2 | 1 | 0 | 0 | 1 |
| Kiyohara[155] 2012 | 2 | 1 | 2 | 2 | 0 | 2 | 1 |
| Dzian[156] 2012 | 2 | 1.5 | 2 | 1 | 0 | 0 | 1 |
| Ada[157] 2012 | 2 | 1 | 2 | 2 | 0 | 2 | 1 |
| López-Cima[159] 2012 | 2 | 1 | 2 | 2 | 2 | 2 | 1 |
| Liu[160] 2012 | 2 | 3 | 2 | 2 | 2 | 1 | 1 |
| Pliarchopoulou[162] 2012 | 2 | 1 | 2 | 2 | 0 | 0 | 1 |
| Wang[50] 2012 | 2 | 1 | 2 | 1 | 2 | 0 | 1 |
| Yao ZG[200] 2012 | 2 | 1 | 2 | 1 | 0 | 0 | 1 |
| Liang KC[242] 2012 | 2 | 1 | 2 | 1 | 0 | 0 | 1 |
| Chen CM[288] 2012 | 2 | 1 | 2 | 1 | 0 | 1 | 1 |
| Shukla[163] 2013 | 2 | 1 | 2 | 1 | 0 | 0 | 1 |
| Shukla[164] 2013 | 2 | 1 | 2 | 1 | 0 | 0 | 1 |
| Piao[165] 2013 | 2 | 3 | 2 | 1 | 0 | 0 | 1 |

| | | | | | | | |
|---|---|---|---|---|---|---|---|
| Lu QG[197] 2013 | 2 | 1 | 2 | 0 | 0 | 0 | 1 |
| Zhang[167] 2014 | 2 | 2 | 2 | 1 | 0 | 0 | 1 |
| Bag[168] 2014 | 2 | 2 | 0 | 1 | 0 | 1 | 1 |
| Pan[169] 2014 | 2 | 3 | 2 | 2 | 2 | 0 | 1 |
| Jiang[170] 2014 | 2 | 3 | 1 | 1 | 2 | 0 | 1 |
| Sharma[171] 2015 | 2 | 1 | 2 | 2 | 2 | 2 | 1 |
| Mota[172] 2015 | 2 | 1 | 2 | 2 | 0 | 1 | 1 |
| Peddireddy[174] 2016 | 2 | 3 | 2 | 2 | 2 | 0 | 1 |
| Masood[175] 2016 | 0 | 0 | 2 | 1 | 0 | 1 | 0 |
| Girdhar[176] 2016 | 2 | 1 | 0 | 1 | 2 | 0 | 1 |
| Wang J[209] 2016 | 2 | 1 | 2 | 1 | 0 | 0 | 1 |
| Wang[179] 2017 | 2 | 1 | 2 | 1 | 0 | 0 | 1 |
| Minina[181] 2017 | 2 | 1 | 2 | 1 | 0 | 0 | 1 |
| Liu AS[191]] 2017 | 2 | 1 | 2 | 1 | 0 | 0 | 1 |
| He[[182] 2018 | 2 | 3 | 2 | 2 | 2 | 0 | 1 |

**Supplemental Table 3 Quality assessment by included studies of meta-analysis of *GSTP1* IIe105Val polymorphism**

| First author/Year | Source of case | Source of control | Ascertainment of cancer | Ascertainment of control | Matching | Genotyping examination | Specimens us determining geno |
|---|---|---|---|---|---|---|---|
| Ryberg[28] 1997 | 2 | 2 | 2 | 1 | 1 | 0 | 0 |
| Saarikoski[36] 1998 | 2 | 2 | 2 | 1 | 0 | 0 | 1 |
| Jourenkova-Mironova[37] 1998 | 2 | 1 | 2 | 1 | 0 | 0 | 1 |
| Harris MJ[248] 1998 | 2 | 1 | 2 | 0 | 0 | 0 | 1 |
| To-Figueras[40] 1999 | 2 | 2 | 2 | 2 | 0 | 0 | 1 |
| Katoh[42] 1999 | 2 | 2 | 2 | 1 | 0 | 0 | 1 |
| Kihara[43] 1999 | 2 | 1 | 2 | 2 | 1 | 0 | 1 |
| Butkiewicz[259] 1999 | 2 | 1 | 2 | 1 | 0 | 1 | 1 |
| Kiyohara[44] 2000 | 2 | 2 | 2 | 1 | 1 | 0 | 1 |
| Risch[57] 2001 | 2 | 1 | 2 | 1 | 0 | 0 | 1 |
| Perera[67] 2002 | 3 | 3 | 2 | 1 | 2 | 1 | 1 |
| Stücker[68] 2002 | 2 | 1 | 2 | 1 | 2 | 0 | 1 |
| Lewis[69] 2002 | 2 | 1 | 2 | 1 | 0 | 0 | 1 |
| Nie LH[249] 2002 | 2 | 1 | 2 | 1 | 2 | 0 | 1 |
| Wang[75] 2003 | 2 | 1 | 2 | 1 | 2 | 0 | 1 |
| Tsai[78] 2003 | 2 | 1 | 1 | 1 | 0 | 0 | 1 |
| Nazar-Stewart[81] 2003 | 3 | 3 | 2 | 1 | 2 | 1 | 1 |
| Lin[84] 2003 | 2 | 1 | 2 | 1 | 0 | 0 | 1 |
| Wang[85] 2003 | 2 | 1 | 2 | 1 | 2 | 0 | 1 |
| Chan-Yeung[95] 2004 | 2 | 3 | 2 | 1 | 0 | 0 | 1 |
| Yang[97] 2004 | 2 | 3 | 2 | 1 | 0 | 0 | 1 |
| Schneider[100] 2004 | 2 | 1 | 2 | 2 | 0 | 0 | 1 |
| Chan[105] 2005 | 0 | 1 | 0 | 1 | 2 | 0 | 1 |
| Skuladottir[108] 2005 | 2 | 2 | 2 | 1 | 2 | 0 | 1 |
| Wenzlaff[109] 2005 | 3 | 3 | 2 | 0 | 2 | 1 | 0 |
| Wenzlaff[109] 2005 | 3 | 3 | 2 | 0 | 2 | 1 | 0 |
| Cote[110] 2005 | 3 | 3 | 2 | 0 | 2 | 1 | 0 |
| Cote[110] 2005 | 3 | 3 | 2 | 0 | 2 | 1 | 0 |
| Cao YF[203]] 2005 | 2 | 1 | 2 | 1 | 2 | 0 | 1 |
| Guo ZL[278] 2005 | 2 | 1 | 2 | 1 | 0 | 0 | 1 |
| Liang GY[277] 2005 | 2 | 1 | 2 | 1 | 2 | 1 | 1 |
| Chen[114] 2006 | 2 | 1 | 2 | 2 | 2 | 1 | 1 |
| Larsen[117] 2006 | 2 | 1 | 2 | 2 | 0 | 0 | 1 |
| Miller[118] 2006 | 2 | 1 | 2 | 1 | 0 | 1 | 1 |
| Zhang TY[215] 2006 | 2 | 1 | 2 | 1 | 2 | 0 | 1 |
| Reszka[119] 2007 | 2 | 1 | 2 | 1 | 2 | 1 | 1 |
| Yang[121] 2007 | 2 | 1 | 2 | 1 | 0 | 0 | 1 |
| Sørensen[122] 2007 | 3 | 3 | 2 | 1 | 0 | 0 | 1 |
| Loft[124] 2007 | 3 | 3 | 2 | 1 | 2 | 1 | 1 |
| Sobti[127] 2008 | 2 | 1 | 2 | 2 | 0 | 0 | 1 |
| Honma[128] 2008 | 2 | 2 | 2 | 1 | 0 | 0 | 1 |
| Zienolddiny[130] 2008 | 2 | 3 | 2 | 1 | 2 | 0 | 1 |
| Yoon[131] 2008 | 2 | 1 | 2 | 1 | 2 | 0 | 1 |
| Sreeja[132] 2008 | 2 | 1 | 2 | 1 | 0 | 0 | 1 |
| Matakova[133] 2009 | 2 | 2 | 2 | 1 | 2 | 2 | 1 |
| Cote[138] 2009 | 3 | 3 | 2 | 1 | 2 | 1 | 1 |
| Cote[138] 2009 | 3 | 3 | 2 | 1 | 2 | 1 | 1 |
| Kumar[139] 2009 | 2 | 0 | 0 | 1 | 0 | 0 | 1 |
| Yue Z[250] 2009 | 2 | 1 | 2 | 0 | 2 | 0 | 1 |
| Yin Q[247] 2009 | 2 | 1 | 2 | 1 | 0 | 0 | 1 |
| Yadav[140] 2010 | 2 | 1 | 2 | 1 | 2 | 1 | 1 |
| Gervasini[142] 2010 | 2 | 1 | 2 | 2 | 2 | 0 | 1 |
| Timofeeva[143] 2010 | 2 | 3 | 0 | 2 | 2 | 1 | 1 |
| Lam[147] 2010 | 2 | 2 | 3 | 1 | 2 | 1 | 1 |
| Hua F[279] 2010 | 2 | 1 | 2 | 2 | 2 | 1 | 1 |
| Ihsan[152] 2011 | 2 | 2 | 2 | 1 | 2 | 1 | 1 |
| Bai TY[289] 2011 | 2 | 1 | 2 | 1 | 0 | 0 | 1 |
| Kiyohara[155] 2012 | 2 | 1 | 2 | 2 | 0 | 2 | 1 |
| Dzian[156] 2012 | 2 | 1 | 2 | 1 | 0 | 0 | 1 |
| Ada[157] 2012 | 2 | 1 | 2 | 2 | 0 | 2 | 1 |
| López-Cima[159] 2012 | 2 | 1 | 2 | 2 | 2 | 2 | 1 |
| Vural[161] 2012 | 2 | 1 | 2 | 2 | 0 | 0 | 1 |
| Pliarchopoulou[162] 2012 | 2 | 1 | 2 | 2 | 0 | 0 | 1 |
| Jiang[170] 2014 | 2 | 3 | 1 | 1 | 2 | 0 | 1 |
| Tao J[204] 2014 | 2 | 1 | 2 | 1 | 0 | 0 | 1 |

| | | | | | | | |
|---|---|---|---|---|---|---|---|
| Sharma[171] 2015 | 2 | 1 | 2 | 2 | 2 | 2 | 1 |
| Mota[172] 2015 | 2 | 1 | 2 | 2 | 0 | 1 | 1 |
| Liu[178] 2016 | 2 | 2 | 2 | 1 | 0 | 0 | 1 |
| Minina[181] 2017 | 2 | 1 | 2 | 1 | 0 | 0 | 1 |

**Supplemental Table 4 Genotype frequencies of the *GSTM1*, *GSTT1*, and *GSTP1* IIe105Val polymorphisms between lung cancer and control groups**

| First author/Year | Ethnicity | *GSTM1* genotype distribution | | | | *GSTT1* genotype distribution | | | | *GSTP1* IIe1 | |
|---|---|---|---|---|---|---|---|---|---|---|---|
| | | Case | | Control | | Case | | Control | | Case | |
| | | present | null | present | null | present | null | present | null | IIe/ IIe | IIe Va |
| Seidegård[1] 1986 | Mixed | 23 | 43 | 46 | 32 | NA | NA | NA | NA | NA | NA |
| Seidegård[2] 1990 | Mixed | 47 | 78 | 66 | 48 | NA | NA | NA | NA | NA | NA |
| Zhong[3] 1991 | Mixed | 130 | 98 | 131 | 94 | NA | NA | NA | NA | NA | NA |
| Heckbert[4] 1992 | Caucasian | 24 | 42 | 50 | 70 | NA | NA | NA | NA | NA | NA |
| Hirvonen[6] 1993 | Mixed | 65 | 73 | 100 | 78 | NA | NA | NA | NA | NA | NA |
| Brockmöller[7] 1993 | Caucasian | 55 | 62 | 174 | 181 | NA | NA | NA | NA | NA | NA |
| Nakachi[8] 1993 | Asian | 33 | 52 | 86 | 84 | NA | NA | NA | NA | NA | NA |
| Nazar-Stewart[9] 1993 | Mixed | 9 | 26 | 23 | 20 | NA | NA | NA | NA | NA | NA |
| Katoh[10] 1994 | Asian | 26 | 27 | 53 | 38 | NA | NA | NA | NA | NA | NA |
| London[13] 1995 | Caucasian | 90 | 94 | 221 | 244 | NA | NA | NA | NA | NA | NA |
| London[13] 1995 | African | 114 | 44 | 183 | 68 | NA | NA | NA | NA | NA | NA |
| Nakajima[14] 1995 | Caucasian | 16 | 11 | 6 | 5 | NA | NA | NA | NA | NA | NA |
| Kihara[15] 1995 | Asian | 197 | 250 | 241 | 228 | NA | NA | NA | NA | NA | NA |
| Katoh[17] 1995 | Asian | 18 | 15 | 53 | 35 | NA | NA | NA | NA | NA | NA |
| Kawajiri[256] 1995 | Asian | 144 | 183 | 191 | 167 | NA | NA | NA | NA | NA | NA |
| Cheng TJ[257] 1995 | Mixed | 32 | 46 | 34 | 44 | NA | NA | NA | NA | NA | NA |
| Moreira[19] 1996 | Caucasian | 55 | 43 | 40 | 44 | NA | NA | NA | NA | NA | NA |
| Ge[20] 1996 | Asian | 30 | 59 | 18 | 35 | NA | NA | NA | NA | NA | NA |
| Deakin[21] 1996 | Caucasian | 56 | 50 | 319 | 386 | 91 | 17 | 526 | 112 | NA | NA |
| el-Zein[22] 1997 | Mixed | 31 | 23 | 27 | 23 | 42 | 12 | 43 | 7 | NA | NA |
| Harrison[25] 1997 | Caucasian | 67 | 101 | 179 | 205 | NA | NA | NA | NA | NA | NA |
| Kelsey[26] 1997 | Mixed | 27 | 33 | 87 | 59 | 50 | 10 | 129 | 17 | NA | NA |
| Kelsey[26] 1997 | African | 84 | 24 | 102 | 30 | 81 | 27 | 103 | 29 | NA | NA |
| Garcia-Closas[27]] 1997 | Mixed | 190 | 226 | 214 | 232 | NA | NA | NA | NA | NA | NA |
| Ryberg[28] 1997 | Caucasian | 61 | 74 | 179 | 163 | NA | NA | NA | NA | 53 | 63 |
| Sun[30] 1997 | Asian | 60 | 147 | 178 | 186 | NA | NA | NA | NA | NA | NA |
| Salagovic[31] 1998 | Caucasian | 48 | 69 | 125 | 123 | 101 | 16 | 206 | 42 | NA | NA |
| Hong[32] 1998 | Asian | 38 | 47 | 30 | 33 | NA | NA | NA | NA | NA | NA |
| Le Marchand[34] 1998 | Mixed | 100 | 135 | 182 | 268 | NA | NA | NA | NA | NA | NA |
| Nyberg[35] 1998 | Caucasian | 100 | 84 | 80 | 81 | NA | NA | NA | NA | NA | NA |
| Saarikoski[36] 1998 | Caucasian | 108 | 100 | 157 | 137 | 178 | 26 | 255 | 39 | 48 | 20 |
| Jourenkova-Mironova[37] 1998 | Caucasian | 69 | 81 | 82 | 90 | 123 | 27 | 145 | 27 | 67 | 66 |
| Gao JR[185] 1998 | Asian | 19 | 27 | 45 | 25 | NA | NA | NA | NA | NA | NA |
| Qu YH[211] 1998 | Asian | 80 | 102 | 85 | 94 | NA | NA | NA | NA | NA | NA |
| Hu YL[236] 1998 | Asian | 25 | 34 | 30 | 29 | NA | NA | NA | NA | NA | NA |
| Harris MJ[248] 1998 | Mixed | NA | NA | NA | NA | NA | NA | NA | NA | 79 | 73 |
| Tang DL[258] 1998 | Mixed | 45 | 60 | 45 | 36 | NA | NA | NA | NA | NA | NA |
| Gao[39] 1999 | Asian | 25 | 34 | 65 | 67 | NA | NA | NA | NA | NA | NA |
| To-Figueras[40] 1999 | Caucasian | 68 | 96 | 167 | 165 | 123 | 41 | 261 | 71 | 83 | 64 |
| Persson[41] 1999 | Asian | 27 | 48 | 40 | 79 | NA | NA | NA | NA | NA | NA |
| Katoh[42] 1999 | Asian | NA | NA | NA | NA | NA | NA | NA | NA | 34 | 13 |
| Kihara[43] 1999 | Asian | 152 | 206 | 126 | 131 | NA | NA | NA | NA | 263 | 78 |
| Butkiewicz[259] 1999 | Caucasian | 79 | 86 | 174 | 151 | NA | NA | NA | NA | 77 | 72 |
| Woodson[261] 1999 | Caucasian | 159 | 160 | 171 | 162 | NA | NA | NA | NA | NA | NA |
| Kiyohara[44] 2000 | Asian | 33 | 53 | 39 | 49 | 39 | 47 | 49 | 39 | 61 | 23 |
| Dresler[45] 2000 | Mixed | 66 | 103 | 66 | 98 | NA | NA | NA | NA | NA | NA |
| London[46] 2000 | Asian | 110 | 122 | 283 | 427 | 98 | 134 | 284 | 426 | NA | NA |
| Ford[47] 2000 | African | 80 | 37 | 96 | 24 | NA | NA | NA | NA | NA | NA |
| Malats[49] 2000 | Caucasian | 56 | 66 | 68 | 53 | 90 | 32 | 77 | 44 | NA | NA |
| Hou[51] 2000 | Caucasian | 132 | 150 | 194 | 181 | NA | NA | NA | NA | NA | NA |

| | | | | | | | | | | | |
|---|---|---|---|---|---|---|---|---|---|---|---|
| Spitz[52] 2000 | Caucasian | 257 | 246 | 226 | 239 | 371 | 132 | 361 | 104 | NA | N[illegible] |
| Cheng[53] 2000 | Asian | 39 | 34 | 16 | 17 | NA | NA | NA | NA | NA | N[illegible] |
| Lan[55] 2000 | Asian | 40 | 82 | 62 | 60 | 49 | 73 | 58 | 64 | NA | N[illegible] |
| Liu[56] 2001 | Mixed | 409 | 517 | 475 | 561 | 819 | 205 | 918 | 258 | NA | N[illegible] |
| Risch[57] 2001 | Caucasian | 183 | 200 | 161 | 185 | 334 | 49 | 281 | 65 | 176 | 17 |
| Quiñones[58] 2001 | Mixed | 33 | 25 | 133 | 41 | NA | NA | NA | NA | NA | N[illegible] |
| Zhao[59] 2001 | Asian | 87 | 146 | 68 | 119 | 101 | 132 | 85 | 102 | NA | N[illegible] |
| Chen[60] 2001 | Asian | 48 | 58 | 67 | 39 | NA | NA | NA | NA | NA | N[illegible] |
| Hou[61] 2001 | Caucasian | 93 | 77 | 70 | 74 | NA | NA | NA | NA | NA | N[illegible] |
| Gsur[63] 2001 | Caucasian | 70 | 64 | 68 | 66 | NA | NA | NA | NA | NA | N[illegible] |
| Hou[64] 2001 | Caucasian | NA | NA | NA | NA | 167 | 17 | 143 | 19 | NA | N[illegible] |
| Perera[67] 2002 | Caucasian | 39 | 47 | 91 | 69 | NA | NA | NA | NA | 33 | 37 |
| Stücker[68] 2002 | Caucasian | 118 | 129 | 136 | 118 | 213 | 38 | 216 | 52 | 120 | 10 |
| Lewis[69] 2002 | Caucasian | 56 | 31 | 68 | 75 | 68 | 19 | 115 | 28 | 34 | 53 |
| Sunaga[70] 2002 | Asian | 105 | 93 | 96 | 56 | 99 | 99 | 93 | 59 | NA | N[illegible] |
| Miller[71] 2002 | Caucasian | 344 | 423 | 423 | 504 | NA | NA | NA | NA | NA | N[illegible] |
| Lu[72] 2002 | Asian | 159 | 155 | 159 | 161 | NA | NA | NA | NA | NA | N[illegible] |
| Zhang[123] 2002 | Asian | 24 | 41 | 33 | 27 | NA | NA | NA | NA | NA | N[illegible] |
| Shi Y[186] 2002 | Asian | 46 | 74 | 67 | 53 | NA | NA | NA | NA | NA | N[illegible] |
| Chan Y[240] 2002 | Asian | 13 | 43 | 34 | 65 | NA | NA | NA | NA | NA | N[illegible] |
| Nie LH[249] 2002 | Asian | NA | NA | NA | NA | NA | NA | NA | NA | 89 | 59 |
| Sgambato[263] 2002 | Caucasian | 8 | 5 | 47 | 53 | NA | NA | NA | NA | NA | N[illegible] |
| Zhang JK[286] 2002 | Asian | 67 | 94 | 73 | 92 | 87 | 74 | 93 | 72 | NA | N[illegible] |
| Cajas-Salazar[74] 2003 | Caucasian | 62 | 48 | 80 | 39 | 79 | 31 | 95 | 24 | NA | N[illegible] |
| Wang[75] 2003 | Asian | NA | NA | NA | NA | 59 | 53 | 65 | 54 | 67 | 44 |
| Pinarbasi[76] 2003 | Caucasian | 53 | 48 | 169 | 37 | NA | NA | NA | NA | NA | N[illegible] |
| Dialyna[77] 2003 | Caucasian | 59 | 63 | 82 | 96 | 101 | 11 | 158 | 20 | NA | N[illegible] |
| Tsai[78] 2003 | Mixed | 98 | 137 | 46 | 48 | 196 | 36 | 74 | 19 | 179 | 54 |
| Kiyohara[79] 2003 | Asian | 64 | 4 | 124 | 135 | NA | NA | NA | NA | NA | N[illegible] |
| Wang[80] 2003 | Asian | 67 | 97 | 91 | 90 | NA | NA | NA | NA | NA | N[illegible] |
| Nazar-Stewart[81] 2003 | Mixed | 131 | 143 | 255 | 246 | 222 | 52 | 410 | 90 | 106 | 11 |
| Hung[82] 2003 | Caucasian | 132 | 152 | 706 | 727 | NA | NA | NA | NA | NA | N[illegible] |
| Taioli[83] 2003 | Caucasian | 111 | 125 | 643 | 639 | 107 | 37 | 698 | 141 | NA | N[illegible] |
| Lin[84] 2003 | Asian | NA | NA | NA | NA | NA | NA | NA | NA | 124 | 74 |
| Wang[85] 2003 | Caucasian | NA | NA | NA | NA | NA | NA | NA | NA | 149 | 17 |
| Oztürk[87] 2003 | Caucasian | 29 | 26 | 33 | 32 | NA | NA | NA | NA | NA | N[illegible] |
| Ruano-Ravina[88] 2003 | Caucasian | 60 | 72 | 100 | 87 | 105 | 27 | 141 | 46 | NA | N[illegible] |
| Chen LJ[232] 2003 | Asian | 14 | 24 | 42 | 57 | NA | NA | NA | NA | NA | N[illegible] |
| Wang S[281] 2003 | Asian | 35 | 61 | 38 | 33 | NA | NA | NA | NA | NA | N[illegible] |
| Wang[90] 2004 | Caucasian | 312 | 404 | 423 | 516 | 573 | 138 | 750 | 185 | NA | N[illegible] |
| Vineis[91] 2004 | Caucasian | 915 | 1,052 | 1,304 | 1,415 | 1,602 | 365 | 2,177 | 542 | NA | N[illegible] |
| Sobti[92] 2004 | Indian | 62 | 38 | 52 | 24 | 82 | 18 | 65 | 11 | NA | N[illegible] |
| Habalová[93] 2004 | Caucasian | 53 | 68 | 69 | 81 | NA | NA | NA | NA | NA | N[illegible] |
| Yang[94] 2004 | Asian | 78 | 108 | 64 | 75 | NA | NA | NA | NA | NA | N[illegible] |
| Chan-Yeung[95] 2004 | Asian | 99 | 130 | 80 | 117 | 86 | 143 | 95 | 102 | 158 | 71 |
| Yang[97] 2004 | Mixed | 113 | 122 | 109 | 124 | 182 | 53 | 158 | 75 | 94 | 11 |
| Alexandrie[98] 2004 | Caucasian | 237 | 287 | 240 | 290 | 456 | 68 | 456 | 74 | NA | N[illegible] |
| Belogubova[99] 2004 | Caucasian | 76 | 91 | 333 | 330 | 137 | 30 | 538 | 125 | NA | N[illegible] |
| Schneider[100] 2004 | Caucasian | 212 | 234 | 328 | 294 | 371 | 75 | 507 | 115 | 198 | 18 |
| Gallegos-Arreola[102] 2003-2004 | Mixed | NA | NA | NA | NA | 41 | 11 | 169 | 9 | NA | N[illegible] |
| Dong CT[129] 2004 | Asian | 34 | 48 | 55 | 36 | NA | NA | NA | NA | NA | N[illegible] |
| Wang N[188] 2004 | Asian | 32 | 45 | 62 | 45 | 33 | 44 | 53 | 54 | NA | N[illegible] |
| Ye WY[194] 2004 | Asian | 23 | 35 | 37 | 25 | NA | NA | NA | NA | NA | N[illegible] |
| Gu YF[210] 2004 | Asian | 79 | 101 | 122 | 102 | NA | NA | NA | NA | NA | N[illegible] |
| Luo CL[213] 2004 | Asian | 18 | 45 | 23 | 24 | NA | NA | NA | NA | NA | N[illegible] |
| Huang XH[227] 2004 | Asian | 35 | 56 | 65 | 73 | NA | NA | NA | NA | NA | N[illegible] |

| | | | | | | | | | | | |
|---|---|---|---|---|---|---|---|---|---|---|---|
| Cao YF[252] 2004 | Asian | 39 | 65 | 110 | 95 | 35 | 69 | 118 | 87 | NA | NA |
| Li WY[29] 2004 | Asian | 90 | 127 | 105 | 95 | NA | NA | NA | NA | NA | NA |
| Li Y[282] 2004 | Asian | 40 | 63 | 77 | 61 | NA | NA | NA | NA | NA | NA |
| Sreeja[104] 2005 | Indian | 100 | 46 | 107 | 39 | 114 | 32 | 133 | 13 | NA | NA |
| Chan[105] 2005 | Asian | 44 | 31 | 71 | 91 | NA | NA | NA | NA | 45 | 28 |
| Adonis[106] 2005 | Mixed | 33 | 22 | 67 | 36 | NA | NA | NA | NA | NA | NA |
| Raimondi[107] 2005 | Caucasian | 242 | 289 | 965 | 1016 | 281 | 97 | 835 | 191 | NA | NA |
| Raimondi[107] 2005 | Asian | 42 | 51 | 92 | 118 | NA | NA | NA | NA | NA | NA |
| Skuladottir[108] 2005 | Caucasian | 165 | 133 | 265 | 284 | 173 | 43 | 326 | 93 | 128 | 13 |
| Wenzlaff[109] 2005 | Caucasian | 62 | 69 | 70 | 77 | 100 | 24 | 115 | 30 | 39 | 58 |
| Wenzlaff[109] 2005 | African | 20 | 9 | 22 | 8 | 25 | 4 | 23 | 7 | 8 | 15 |
| Cote[110] 2005 | Caucasian | 123 | 107 | 151 | 136 | 169 | 47 | 228 | 52 | 79 | 12 |
| Cote[110] 2005 | African | 64 | 26 | 88 | 31 | 71 | 17 | 92 | 26 | 16 | 60 |
| Brennan[111] 2005 | Caucasian | 1,043 | 1,023 | 1,097 | 986 | 1,775 | 340 | 1,796 | 344 | NA | NA |
| Li DR[192] 2005 | Asian | 42 | 57 | 39 | 27 | NA | NA | NA | NA | NA | NA |
| Qiao GB[201] 2005 | Asian | 83 | 130 | 104 | 95 | NA | NA | NA | NA | NA | NA |
| Cao YF[203] 2005 | Asian | NA | NA | NA | NA | NA | NA | NA | NA | 66 | 26 |
| Yuan TZ[205]] 2005 | Asian | NA | NA | NA | NA | 68 | 82 | 94 | 58 | NA | NA |
| Luo CL[216] 2005 | Asian | 18 | 45 | 53 | 86 | NA | NA | NA | NA | NA | NA |
| Barnholtz-Sloan[265] 2005 | Caucasian | 108 | 84 | 188 | 175 | NA | NA | NA | NA | NA | NA |
| Barnholtz-Sloan[265] 2005 | African | 44 | 16 | 93 | 38 | NA | NA | NA | NA | NA | NA |
| Guo ZL[278] 2005 | Asian | NA | NA | NA | NA | NA | NA | NA | NA | 74 | 67 |
| Chou YC[262] 2005 | Asian | 12 | 18 | 21 | 39 | NA | NA | NA | NA | NA | NA |
| Li DR[260] 2005 | Asian | 70 | 80 | 91 | 61 | NA | NA | NA | NA | NA | NA |
| Liang GY[277] 2005 | Asian | 96 | 131 | 103 | 124 | 108 | 119 | 140 | 87 | 135 | 83 |
| Lee[113] 2006 | Asian | 71 | 98 | 91 | 105 | 89 | 80 | 89 | 107 | NA | NA |
| Chen[114] 2006 | Asian | 37 | 60 | 108 | 89 | 38 | 59 | 112 | 85 | 66 | 31 |
| Pisani[115] 2006 | Asian | 67 | 99 | 105 | 184 | NA | NA | NA | NA | NA | NA |
| Larsen[117] 2006 | Caucasian | 503 | 591 | 258 | 367 | 862 | 226 | 510 | 114 | 501 | 48 |
| Miller[118] 2006 | Caucasian | NA | NA | NA | NA | NA | NA | NA | NA | 885 | 81 |
| Qian[65] 2006 | Asian | 39 | 69 | 55 | 53 | NA | NA | NA | NA | NA | NA |
| Chang FH[126] 2006 | Asian | 57 | 106 | 85 | 78 | NA | NA | NA | NA | NA | NA |
| Wang QM[184] 2006 | Asian | 16 | 40 | 23 | 19 | NA | NA | NA | NA | NA | NA |
| He DX[214] 2006 | Asian | NA | NA | NA | NA | 28 | 33 | 17 | 29 | NA | NA |
| Zhang TY[215] 2006 | Asian | NA | NA | NA | NA | NA | NA | NA | NA | 59 | 46 |
| Wang DQ[229] 2006 | Asian | 35 | 56 | 40 | 51 | NA | NA | NA | NA | NA | NA |
| Reszka[119] 2007 | Caucasian | 207 | 163 | 192 | 146 | 99 | 20 | 99 | 39 | 108 | 10 |
| Osawa[120] 2007 | Asian | 56 | 57 | 59 | 62 | NA | NA | NA | NA | NA | NA |
| Yang[121] 2007 | Asian | 159 | 158 | 167 | 179 | 148 | 168 | 179 | 166 | 198 | 10 |
| Sørensen[122] 2007 | Caucasian | 186 | 242 | 372 | 392 | 362 | 67 | 655 | 108 | 194 | 18 |
| Loft[124] 2007 | Caucasian | 112 | 137 | 101 | 156 | 212 | 37 | 242 | 15 | 116 | 10 |
| Wang YS[266] 2007 | Asian | 38 | 56 | 38 | 56 | NA | NA | NA | NA | NA | NA |
| Li SF[268] 2007 | Asian | 18 | 24 | 46 | 57 | 25 | 17 | 55 | 48 | NA | NA |
| Hu XG[274] 2007 | Asian | 36 | 76 | 46 | 58 | NA | NA | NA | NA | NA | NA |
| Shah[125] 2008 | Indian | 111 | 89 | 152 | 48 | NA | NA | NA | NA | NA | NA |
| Sobti[127] 2008 | Indian | 88 | 63 | 98 | 53 | 124 | 27 | 131 | 20 | 78 | 68 |
| Honma[128] 2008 | Mixed | 109 | 91 | 137 | 127 | 173 | 27 | 230 | 34 | 82 | 93 |
| Zienolddiny[130] 2008 | Caucasian | 139 | 56 | 149 | 55 | NA | NA | NA | NA | 179 | 12 |
| Yoon[131] 2008 | Asian | NA | NA | NA | NA | NA | NA | NA | NA | 137 | 65 |
| Sreeja[132] 2008 | Indian | 138 | 73 | 147 | 64 | 146 | 65 | 183 | 28 | 116 | 74 |
| Liu Q[66] 2008 | Asian | 44 | 66 | 68 | 57 | NA | NA | NA | NA | NA | NA |
| Qi XS[220] 2008 | Asian | NA | NA | NA | NA | 36 | 17 | 45 | 27 | NA | NA |
| Xia Y[246] 2008 | Asian | 24 | 34 | 55 | 76 | NA | NA | NA | NA | NA | NA |
| Hou Y[273] 2008 | Asian | 32 | 45 | 29 | 48 | NA | NA | NA | NA | NA | NA |
| Matakova[133]] 2009 | Caucasian | 66 | 94 | 115 | 105 | 119 | 41 | 174 | 46 | 81 | 66 |
| Klinchid[134] 2009 | Asian | 26 | 61 | 34 | 47 | 53 | 34 | 42 | 39 | NA | NA |

| | | | | | | | | | | |
|---|---|---|---|---|---|---|---|---|---|---|
| Carpenter[135] 2009 | Mixed | 184 | 127 | 346 | 276 | NA | NA | NA | NA | NA | NA |
| Zupa[136] 2009 | Caucasian | 33 | 42 | 53 | 68 | NA | NA | NA | NA | NA | NA |
| Lam[137] 2009 | Mixed | 62 | 75 | 219 | 222 | 106 | 29 | 335 | 87 | NA | NA |
| Cote[138] 2009 | Caucasian | 178 | 210 | 206 | 197 | 311 | 77 | 324 | 82 | 171 | 17 |
| Cote[138] 2009 | African | 78 | 36 | 93 | 28 | 87 | 28 | 95 | 26 | 28 | 61 |
| Kumar[139] 2009 | Indian | 49 | 44 | 154 | 99 | 69 | 24 | 197 | 56 | 55 | 35 |
| Qi XS[219] 2009 | Asian | 19 | 34 | 31 | 41 | NA | NA | NA | NA | NA | NA |
| Wang MJ[230] 2009 | Asian | 161 | 143 | 383 | 258 | NA | NA | NA | NA | NA | NA |
| Yue Z[250] 2009 | Asian | NA | NA | NA | NA | NA | NA | NA | NA | 64 | 36 |
| Chen H[275] 2009 | Asian | 59 | 99 | 208 | 247 | NA | NA | NA | NA | NA | NA |
| Liu JN[276] 2009 | Asian | NA | NA | NA | NA | 43 | 57 | 79 | 56 | NA | NA |
| Yin Q[247] 2009 | Asian | NA | NA | NA | NA | NA | NA | NA | NA | 62 | 51 |
| Yadav[140] 2010 | Indian | 82 | 19 | 152 | 69 | 68 | 33 | 118 | 103 | 54 | 47 |
| Jin[141] 2010 | Asian | 55 | 95 | 71 | 79 | NA | NA | NA | NA | NA | NA |
| Gervasini[142] 2010 | Caucasian | 56 | 47 | 127 | 120 | 87 | 16 | 206 | 41 | 53 | 50 |
| Timofeeva[143] 2010 | Caucasian | 279 | 334 | 607 | 644 | 511 | 101 | 1035 | 214 | 279 | 26 |
| Cabral[144] 2010 | Caucasian | 16 | 26 | 42 | 33 | 21 | 21 | 39 | 36 | NA | NA |
| Cabral[144] 2010 | African | 8 | 14 | 5 | 7 | 17 | 5 | 7 | 5 | NA | NA |
| Altinisik[145] 2010 | Caucasian | 47 | 28 | 40 | 15 | 58 | 17 | 46 | 9 | NA | NA |
| Sreelekha[146] 2010 | Indian | 63 | 23 | 50 | 10 | 52 | 34 | 56 | 4 | NA | NA |
| Lam[147] 2010 | Caucasian | NA | NA | NA | NA | NA | NA | NA | NA | 853 | 73 |
| Song B[234] 2010 | Asian | 51 | 74 | 70 | 55 | NA | NA | NA | NA | NA | NA |
| Hua F[279] 2010 | Asian | NA | NA | NA | NA | NA | NA | NA | NA | 171 | 88 |
| Zheng DJ[287] 2010 | Asian | 115 | 150 | 132 | 175 | NA | NA | NA | NA | NA | NA |
| Zhu XX[291] 2010 | Asian | 67 | 93 | 88 | 72 | NA | NA | NA | NA | NA | NA |
| Tamaki[148] 2011 | Asian | 106 | 86 | 101 | 102 | 95 | 97 | 99 | 104 | NA | NA |
| Kohno[149] 2011 | Asian | 174 | 200 | 159 | 158 | NA | NA | NA | NA | NA | NA |
| Young[150] 2011 | Caucasian | 173 | 274 | 465 | 680 | NA | NA | NA | NA | NA | NA |
| Singh[151] 2010-2011 | Indian | 129 | 71 | 147 | 53 | NA | NA | NA | NA | NA | NA |
| Ihsan[152] 2011 | Indian | 122 | 66 | 177 | 113 | 155 | 33 | 217 | 73 | 102 | 77 |
| Atinkaya[153] 2012 | Caucasian | 72 | 53 | 59 | 56 | 102 | 25 | 100 | 22 | NA | NA |
| Fowke[154] 2011 | Asian | 98 | 110 | 329 | 456 | 100 | 108 | 403 | 381 | NA | NA |
| Bai TY[207] 2011 | Asian | NA | NA | NA | NA | 56 | 50 | 139 | 111 | NA | NA |
| Zhang JQ[226] 2011 | Asian | 33 | 17 | 43 | 7 | NA | NA | NA | NA | NA | NA |
| Ai C[231] 2011 | Asian | 14 | 36 | 27 | 23 | NA | NA | NA | NA | NA | NA |
| Zhou XL[271] 2011 | Asian | 87 | 122 | 119 | 89 | NA | NA | NA | NA | NA | NA |
| Bai TY[289] 2011 | Asian | 49 | 79 | 125 | 89 | 72 | 56 | 118 | 96 | 70 | 48 |
| Du GB[290] 2011 | Asian | 52 | 73 | 54 | 71 | 68 | 57 | 69 | 56 | NA | NA |
| Kiyohara[155] 2012 | Asian | 194 | 268 | 194 | 185 | 245 | 217 | 215 | 164 | 323 | 12 |
| Dzian[156] 2012 | Caucasian | 100 | 130 | 130 | 160 | 171 | 59 | 242 | 48 | 115 | 91 |
| Ada[157] 2012 | Caucasian | 90 | 123 | 107 | 124 | 162 | 51 | 188 | 43 | 133 | 80 |
| López-Cima[159] 2012 | Caucasian | 375 | 401 | 358 | 418 | 618 | 158 | 611 | 165 | 352 | 33 |
| Liu[160] 2012 | Asian | 215 | 145 | 253 | 107 | NA | NA | NA | NA | NA | NA |
| Vural[161] 2012 | Caucasian | NA | NA | NA | NA | NA | NA | NA | NA | 52 | 32 |
| Pliarchopoulou[162] 2012 | Caucasian | 45 | 55 | 79 | 46 | NA | NA | NA | NA | 52 | 41 |
| Wang N[50] 2012 | Asian | 87 | 122 | 143 | 113 | 119 | 90 | 156 | 100 | NA | NA |
| Yao ZG[200] 2012 | Asian | 54 | 96 | 82 | 68 | NA | NA | NA | NA | NA | NA |
| Liang KC[242] 2012 | Asian | 21 | 47 | 31 | 39 | 23 | 45 | 36 | 34 | NA | NA |
| Chen CM[288] 2012 | Asian | 77 | 123 | 89 | 110 | NA | NA | NA | NA | NA | NA |
| Shukla[163] 2013 | Indian | 134 | 84 | 148 | 90 | 136 | 82 | 180 | 58 | NA | NA |
| Shukla[164] 2013 | Indian | 134 | 84 | 80 | 124 | 136 | 82 | 168 | 36 | NA | NA |
| Piao[165] 2013 | Asian | 1,696 | 2,237 | 776 | 923 | 1,863 | 2,070 | 841 | 858 | NA | NA |
| Lu QG[197] 2013 | Asian | 30 | 61 | 68 | 70 | NA | NA | NA | NA | NA | NA |
| Zhang[167]] 2014 | Asian | 44 | 66 | 58 | 42 | 34 | 76 | 47 | 53 | NA | NA |
| Bag[168] 2014 | Indian | NA | NA | NA | NA | 20 | 6 | 28 | 5 | NA | NA |
| Pan[169] 2014 | Asian | 218 | 305 | 299 | 224 | 253 | 270 | 311 | 212 | NA | NA |

| | | | | | | | | | | | |
|---|---|---|---|---|---|---|---|---|---|---|---|
| Jiang[170] 2014 | Asian | 132 | 190 | 268 | 188 | 175 | 147 | 253 | 203 | 174 | 13 |
| Tao J[204] 2014 | Asian | NA | NA | NA | NA | NA | NA | NA | NA | 70 | 69 |
| Sharma[171] 2015 | Indian | 136 | 134 | 167 | 103 | 220 | 50 | 233 | 37 | 225 | 40 |
| Mota[172] 2015 | Caucasian | 108 | 75 | 127 | 101 | 122 | 61 | 174 | 54 | 71 | 94 |
| Peddireddy[174] 2016 | Indian | 182 | 64 | 187 | 63 | 200 | 46 | 224 | 26 | NA | N[illegible] |
| Masood[175] 2016 | Indian | 197 | 53 | 212 | 58 | 230 | 20 | 246 | 24 | NA | N[illegible] |
| Girdhar[176] 2016 | Indian | 160 | 160 | 198 | 122 | 258 | 62 | 263 | 57 | NA | N[illegible] |
| Liu[[178] 2016 | Asian | NA | NA | NA | NA | NA | NA | NA | NA | 215 | 80 |
| Wang J[209] 2016 | Asian | 48 | 102 | 78 | 72 | NA | NA | NA | NA | NA | N[illegible] |
| Wang[179] 2017 | Asian | 82 | 118 | 112 | 88 | 114 | 86 | 122 | 78 | NA | N[illegible] |
| Minina[181] 2017 | Caucasian | 210 | 143 | 172 | 128 | 267 | 86 | 233 | 67 | 135 | 18 |
| Liu AS[191] 2017 | Asian | 27 | 44 | 45 | 26 | NA | NA | NA | NA | NA | N[illegible] |
| He[182] 2018 | Asian | 179 | 134 | 217 | 113 | NA | NA | NA | NA | NA | N[illegible] |

NA = not available, HWE = Hardy-Weinberg equilibrium, [a] = IIe/Val + Val/Val

**Supplemental Table 5 Genotype frequencies of the *GSTM1, GSTT1, and GSTP1* IIe105Val polymorphisms between lung cancer and control groups by histological type**

| First author/Year | Ethnicity | *GSTM1* genotype distribution | | | | *GSTT1* genotype distribution | | | | *GSTP1* IIe10 | |
|---|---|---|---|---|---|---|---|---|---|---|---|
| | | Case | | Control | | Case | | Control | | Case | |
| | | present | null | present | null | present | null | present | null | IIe/IIe | IIe |
| Small cell carcinoma | | | | | | | | | | | |
| Seidegård[1] 1986 | Mixed | 3 | 6 | 46 | 32 | NA | NA | NA | NA | NA | N |
| Seidegård[2] 1990 | Mixed | 0 | 3 | 66 | 48 | NA | NA | NA | NA | NA | N |
| Brockmöller[7] 1993 | Caucasian | 6 | 8 | 174 | 181 | NA | NA | NA | NA | NA | N |
| Katoh[10] 1994 | Asian | 5 | 6 | 28 | 20 | NA | NA | NA | NA | NA | N |
| London[13] 1995 | Mixed | 29 | 17 | 404 | 312 | NA | NA | NA | NA | NA | N |
| Kihara[15] 1995 | Asian | 24 | 41 | 241 | 228 | NA | NA | NA | NA | NA | N |
| Moreira[19] 1996 | Caucasian | 10 | 9 | 40 | 44 | NA | NA | NA | NA | NA | N |
| To-Figueras[24] 1997 | Caucasian | 21 | 35 | 248 | 64 | 43 | 13 | 157 | 155 | NA | N |
| Sun[30] 1997 | Asian | 13 | 40 | 178 | 186 | NA | NA | NA | NA | NA | N |
| Hong[32] 1998 | Asian | 8 | 6 | 30 | 33 | NA | NA | NA | NA | NA | N |
| Jourenkova-Mironova[37] 1998 | Caucasian | 27 | 25 | 82 | 90 | 40 | 12 | 145 | 27 | 21 | 25 |
| To-Figueras[40] 1999 | Caucasian | NA | NA | NA | NA | NA | NA | NA | NA | 27 | 23 |
| Kihara[43] 1999 | Asian | 29 | 44 | 126 | 131 | NA | NA | NA | NA | 58 | 14 |
| Woodson[261] 1999 | Caucasian | 22 | 25 | 171 | 162 | NA | NA | NA | NA | NA | N |
| Ford[47] 2000 | African | 7 | 4 | 96 | 24 | NA | NA | NA | NA | NA | N |
| Hou[51] 2000 | Caucasian | 19 | 22 | 194 | 181 | NA | NA | NA | NA | NA | N |
| Stücker[68] 2002 | Caucasian | 20 | 29 | 136 | 118 | 42 | 6 | 216 | 52 | 48 | 23 |
| Lewis[69] 2002 | Caucasian | 11 | 4 | 68 | 75 | 11 | 4 | 115 | 28 | 4 | 10 |
| Reszka[73] 2003 | Caucasian | 17 | 18 | 101 | 64 | NA | NA | NA | NA | 22 | 13 |
| Pinarbasi[76] 2003 | Caucasian | 14 | 12 | 169 | 37 | NA | NA | NA | NA | NA | N |
| Nazar-Stewart[81] 2003 | Mixed | 25 | 27 | 255 | 246 | 41 | 11 | 410 | 90 | 17 | 25 |
| Sobti[92] 2004 | Indian | 15 | 9 | 52 | 24 | 22 | 2 | 65 | 11 | NA | N |
| Alexandrie[98] 2004 | Caucasian | 40 | 62 | 240 | 290 | 92 | 10 | 456 | 74 | NA | N |
| Schneider[100] 2004 | Caucasian | 33 | 34 | 328 | 294 | 54 | 13 | 507 | 115 | 31 | 27 |
| Sørensen[101] 2004 | Caucasian | NA | NA | NA | NA | 47 | 4 | 223 | 16 | NA | N |
| Li Y[282]] 2004 | Asian | 8 | 13 | 77 | 61 | NA | NA | NA | NA | NA | N |
| Lee[113] 2006 | Asian | 19 | 11 | 91 | 105 | 12 | 18 | 89 | 107 | NA | N |
| Miller[118] 2006 | Caucasian | NA | NA | NA | NA | NA | NA | NA | NA | 69 | 80 |
| Sobti[127] 2008 | Indian | 14 | 10 | 98 | 53 | 19 | 5 | 131 | 20 | 15 | 9[a] |
| Honma[128] 2008 | Mixed | 18 | 14 | 137 | 127 | 25 | 7 | 230 | 34 | 111 | 21 |
| Liu JN[276] 2009 | Asian | NA | NA | NA | NA | 10 | 8 | 79 | 56 | NA | N |
| Gervasini[142] 2010 | Caucasian | 5 | 10 | 127 | 120 | 12 | 3 | 206 | 41 | 6 | 9[a] |
| Cabral[144] 2010 | Mixed | 3 | 8 | 37 | 50 | 5 | 6 | 46 | 41 | NA | N |
| Altinisik[145] 2010 | Caucasian | 11 | 3 | 40 | 15 | 12 | 2 | 46 | 9 | NA | N |
| Zheng DJ[287] 2010 | Asian | 12 | 11 | 132 | 175 | NA | NA | NA | NA | NA | N |
| Young[150] 2011 | Caucasian | 31 | 47 | 465 | 680 | NA | NA | NA | NA | NA | N |
| Du GB[290] 2011 | Asian | 5 | 12 | 54 | 71 | 7 | 10 | 69 | 56 | NA | N |
| Ada[157] 2012 | Caucasian | 16 | 16 | 107 | 124 | 31 | 1 | 188 | 43 | 18 | 14 |
| López-Cima[159] 2012 | Caucasian | 56 | 72 | 358 | 418 | 103 | 25 | 611 | 165 | 59 | 71 |
| Liu[160] 2012 | Asian | 21 | 16 | 253 | 107 | NA | NA | NA | NA | NA | N |
| Vural[161] 2012 | Caucasian | NA | NA | NA | NA | NA | NA | NA | NA | 52 | 32 |
| Tao J[204] 2014 | Asian | NA | NA | NA | NA | NA | NA | NA | NA | 6 | 8[a] |
| Sharma[171] 2015 | Indian | 34 | 35 | 167 | 103 | 55 | 14 | 233 | 37 | 51 | 15 |
| Girdhar[176] 2016 | Indian | 35 | 41 | 198 | 122 | 61 | 15 | 263 | 57 | NA | N |
| Wang J[209] 2016 | Asian | 8 | 12 | 78 | 72 | NA | NA | NA | NA | NA | N |
| Large cell carcinoma | | | | | | | | | | | |
| Seidegård[1] 1986 | Mixed | 0 | 1 | 46 | 32 | NA | NA | NA | NA | NA | N |
| Seidegård[2] 1990 | Mixed | 4 | 5 | 66 | 48 | NA | NA | NA | NA | NA | N |
| Brockmöller[7] 1993 | Caucasian | 16 | 14 | 174 | 181 | NA | NA | NA | NA | NA | N |
| Kihara[15] 1995 | Asian | 5 | 1 | 241 | 228 | NA | NA | NA | NA | NA | N |

| | | | | | | | | | | |
|---|---|---|---|---|---|---|---|---|---|---|
| To-Figueras[24] 1997 | Caucasian | 9 | 3 | 248 | 64 | 10 | 2 | 157 | 155 | NA | NA |
| To-Figueras[40] 1999 | Caucasian | NA | NA | NA | NA | NA | NA | NA | NA | 6 | 6 |
| Pinarbasi[76] 2003 | Caucasian | 5 | 6 | 169 | 37 | NA | NA | NA | NA | NA | NA |
| Schneider[100] 2004 | Caucasian | 9 | 7 | 328 | 294 | 13 | 3 | 507 | 115 | 5 | 8 |
| Miller[118] 2006 | Caucasian | NA | NA | NA | NA | NA | NA | NA | NA | 69 | 62 |
| Gervasini[142] 2010 | Caucasian | 9 | 14 | 127 | 120 | 22 | 1 | 206 | 41 | 11 | 12 |
| Squamous carcinoma | | | | | | | | | | | |
| Seidegård[1] 1986 | Mixed | 11 | 16 | 46 | 32 | NA | NA | NA | NA | NA | NA |
| Seidegård[2] 1990 | Mixed | 16 | 19 | 66 | 48 | NA | NA | NA | NA | NA | NA |
| Zhong[3] 1991 | Mixed | 52 | 48 | 131 | 94 | NA | NA | NA | NA | NA | NA |
| Hirvonen[6] 1993 | Mixed | 27 | 44 | 80 | 62 | NA | NA | NA | NA | NA | NA |
| Hirvonen[6] 1993 | Mixed | 27 | 44 | 20 | 16 | NA | NA | NA | NA | NA | NA |
| Brockmöller[7] 1993 | Caucasian | 17 | 24 | 174 | 181 | NA | NA | NA | NA | NA | NA |
| Nakachi[8] 1993 | Asian | 33 | 52 | 86 | 84 | NA | NA | NA | NA | NA | NA |
| Katoh[10] 1994 | Asian | 11 | 12 | 28 | 20 | NA | NA | NA | NA | NA | NA |
| London[13] 1995 | Mixed | 44 | 38 | 404 | 312 | NA | NA | NA | NA | NA | NA |
| Kihara[15] 1995 | Asian | 60 | 80 | 241 | 228 | NA | NA | NA | NA | NA | NA |
| Kawajiri[256] 1995 | Asian | 39 | 65 | 191 | 167 | NA | NA | NA | NA | NA | NA |
| Moreira[19] 1996 | Caucasian | 33 | 20 | 40 | 44 | NA | NA | NA | NA | NA | NA |
| el-Zein[22] 1997 | Mixed | 13 | 10 | 27 | 23 | 18 | 5 | 43 | 7 | NA | NA |
| To-Figueras[24] 1997 | Caucasian | 20 | 30 | 155 | 37 | 35 | 15 | 97 | 95 | NA | NA |
| To-Figueras[24] 1997 | Caucasian | 20 | 30 | 93 | 27 | 35 | 15 | 60 | 60 | NA | NA |
| Ryberg[28] 1997 | Caucasian | 25 | 41 | 179 | 163 | NA | NA | NA | NA | 20 | 34 |
| Sun[30] 1997 | Asian | 25 | 61 | 178 | 186 | NA | NA | NA | NA | NA | NA |
| Hong[32] 1998 | Asian | 12 | 15 | 30 | 33 | NA | NA | NA | NA | NA | NA |
| Le Marchand[34] 1998 | Mixed | 27 | 47 | 182 | 268 | NA | NA | NA | NA | NA | NA |
| Saarikoski[36] 1998 | Caucasian | 44 | 50 | 157 | 137 | 76 | 15 | 255 | 39 | NA | NA |
| Jourenkova-Mironova[37] 1998 | Caucasian | 42 | 56 | 82 | 90 | 83 | 15 | 145 | 27 | 46 | 41 |
| Gao[39] 1999 | Asian | 13 | 10 | 29 | 30 | NA | NA | NA | NA | NA | NA |
| Gao[39] 1999 | Asian | 13 | 10 | 36 | 37 | NA | NA | NA | NA | NA | NA |
| To-Figueras[40] 1999 | Caucasian | NA | NA | NA | NA | NA | NA | NA | NA | 29 | 20 |
| Kihara[43] 1999 | Asian | 56 | 69 | 126 | 131 | NA | NA | NA | NA | 84 | 32 |
| Woodson[261] 1999 | Caucasian | 78 | 69 | 171 | 162 | NA | NA | NA | NA | NA | NA |
| Butkiewicz[259] 1999 | Caucasian | 56 | 54 | 174 | 151 | NA | NA | NA | NA | 50 | 52 |
| Ford[47] 2000 | African | 30 | 15 | 96 | 24 | NA | NA | NA | NA | NA | NA |
| Malats[49] 2000 | Caucasian | 9 | 16 | 68 | 53 | 18 | 7 | 68 | 53 | NA | NA |
| Hou[51] 2000 | Caucasian | 53 | 76 | 194 | 181 | NA | NA | NA | NA | NA | NA |
| Liu[56] 2001 | Mixed | 92 | 144 | 475 | 561 | 205 | 52 | 918 | 258 | NA | NA |
| Risch[57] 2001 | Caucasian | 70 | 97 | 161 | 185 | 149 | 18 | 281 | 65 | 76 | 77 |
| Gsur[63] 2001 | Caucasian | 33 | 25 | 68 | 66 | NA | NA | NA | NA | NA | NA |
| Stücker[68] 2002 | Caucasian | 63 | 50 | 136 | 118 | 97 | 18 | 216 | 52 | 54 | 46 |
| Lewis[69] 2002 | Caucasian | 21 | 8 | 68 | 75 | 23 | 6 | 115 | 28 | 14 | 17 |
| Lu[72] 2002 | Asian | 89 | 88 | 159 | 161 | NA | NA | NA | NA | NA | NA |
| Zhang[123] 2002 | Asian | 11 | 21 | 33 | 27 | NA | NA | NA | NA | NA | NA |
| Qiao G[264] 2002 | Asian | 42 | 64 | 104 | 95 | NA | NA | NA | NA | NA | NA |
| Zhang JK[296] 2002 | Asian | 25 | 29 | 73 | 92 | 29 | 25 | 72 | 93 | NA | NA |
| Nie LH[249] 2002 | Asian | NA | NA | NA | NA | NA | NA | NA | NA | 35 | 22 |
| Reszka[73] 2003 | Caucasian | 40 | 21 | 101 | 64 | NA | NA | NA | NA | 33 | 28 |
| Pinarbasi[76] 2003 | Caucasian | 19 | 24 | 169 | 37 | NA | NA | NA | NA | NA | NA |
| Nazar-Stewart[81] 2003 | Mixed | 37 | 44 | 255 | 246 | 67 | 14 | 410 | 90 | 35 | 29 |
| Lin[84] 2003 | Asian | NA | NA | NA | NA | NA | NA | NA | NA | 47 | 36 |
| Wang S[281] 2003 | Asian | 13 | 23 | 38 | 33 | NA | NA | NA | NA | NA | NA |
| Sobti[92] 2004 | Indian | 42 | 29 | 52 | 24 | 56 | 15 | 65 | 11 | NA | NA |
| Chan-Yeung[95] 2004 | Asian | 14 | 24 | 80 | 117 | 15 | 23 | 95 | 102 | 29 | 9[a] |
| Alexandrie[98] 2004 | Caucasian | 85 | 81 | 240 | 290 | 142 | 24 | 456 | 74 | NA | NA |
| Belogubova[99] 2004 | Caucasian | 37 | 51 | 178 | 146 | 71 | 17 | 261 | 63 | NA | NA |

| | | | | | | | | | | |
|---|---|---|---|---|---|---|---|---|---|---|
| Belogubova[99] 2004 | Caucasian | 37 | 51 | 155 | 184 | 71 | 17 | 277 | 62 | NA | NA |
| Schneider[100] 2004 | Caucasian | 83 | 100 | 328 | 294 | 144 | 39 | 507 | 115 | 81 | 75 |
| Sørensen[101] 2004 | Caucasian | NA | NA | NA | NA | 44 | 12 | 225 | 15 | NA | NA |
| Wang N[188] 2004 | Asian | 17 | 25 | 62 | 45 | 15 | 27 | 53 | 54 | NA | NA |
| Huang XH[227] 2004 | Asian | 21 | 33 | 65 | 73 | NA | NA | NA | NA | NA | NA |
| Li Y[282] 2004 | Asian | 26 | 38 | 77 | 61 | NA | NA | NA | NA | NA | NA |
| Raimondi[107] 2005 | Caucasian | 28 | 35 | 668 | 700 | 35 | 8 | 582 | 148 | NA | NA |
| Qiao GB[201] 2005 | Asian | 42 | 64 | 104 | 95 | NA | NA | NA | NA | NA | NA |
| Yuan TZ[205] 2005 | Asian | NA | NA | NA | NA | 25 | 45 | 94 | 58 | NA | NA |
| Li DR[260] 2005 | Asian | 36 | 34 | 91 | 61 | NA | NA | NA | NA | NA | NA |
| Liang GY[277] 2005 | Asian | 41 | 53 | 103 | 124 | 39 | 55 | 140 | 87 | 58 | 32 |
| Guo ZL[278] 2005 | Asian | NA | NA | NA | NA | NA | NA | NA | NA | 27 | 43 |
| Lee[113] 2006 | Asian | 24 | 49 | 91 | 105 | 39 | 34 | 89 | 107 | NA | NA |
| Larsen[117] 2006 | Caucasian | 227 | 266 | 258 | 367 | 399 | 92 | 510 | 114 | 230 | 21 |
| Miller[118] 2006 | Caucasian | NA | NA | NA | NA | NA | NA | NA | NA | 190 | 17 |
| Zhang TY[215] 2006 | Asian | NA | NA | NA | NA | NA | NA | NA | NA | 21 | 27 |
| Osawa[120] 2007 | Asian | 17 | 18 | 59 | 62 | NA | NA | NA | NA | NA | NA |
| Sobti[127] 2008 | Indian | 59 | 37 | 98 | 53 | 81 | 15 | 131 | 20 | 51 | 45 |
| Qi XS[220] 2008 | Asian | NA | NA | NA | NA | 18 | 9 | 45 | 27 | NA | NA |
| Qi XS[219] 2009 | Asian | 11 | 16 | 31 | 41 | NA | NA | NA | NA | NA | NA |
| Chen H[275] 2009 | Asian | 36 | 50 | 208 | 247 | NA | NA | NA | NA | NA | NA |
| Liu JN[276] 2009 | Asian | NA | NA | NA | NA | 10 | 19 | 79 | 56 | NA | NA |
| Gervasini[142] 2010 | Caucasian | 32 | 17 | 127 | 120 | 39 | 10 | 206 | 41 | 26 | 23 |
| Altinisik[145] 2010 | Caucasian | 19 | 13 | 40 | 15 | 24 | 8 | 46 | 9 | NA | NA |
| Song B[234] 2010 | Asian | 51 | 74 | 70 | 55 | NA | NA | NA | NA | NA | NA |
| Zheng DJ[287] 2010 | Asian | 41 | 79 | 132 | 175 | NA | NA | NA | NA | NA | NA |
| Hua F[279] 2010 | Asian | NA | NA | NA | NA | NA | NA | NA | NA | 77 | 41 |
| Young[150] 2011 | Caucasian | 41 | 66 | 465 | 680 | NA | NA | NA | NA | NA | NA |
| Singh[151] 2010-2011 | Indian | 129 | 71 | 147 | 53 | NA | NA | NA | NA | NA | NA |
| Zhou XL[271]] 2011 | Asian | 40 | 63 | 119 | 89 | NA | NA | NA | NA | NA | NA |
| Fan J[272] 2011 | Asian | 10 | 16 | 31 | 39 | 12 | 14 | 36 | 34 | NA | NA |
| Du GB[290] 2011 | Asian | 25 | 32 | 54 | 71 | 33 | 24 | 69 | 56 | NA | NA |
| Dzian[156] 2012 | Caucasian | 46 | 66 | 130 | 160 | 88 | 24 | 242 | 48 | 56 | 45 |
| Ada[157] 2012 | Caucasian | 32 | 34 | 107 | 124 | 51 | 15 | 188 | 43 | 37 | 29 |
| López-Cima[159] 2012 | Caucasian | 160 | 149 | 358 | 418 | 243 | 66 | 611 | 165 | 132 | 17 |
| Liu[160] 2012 | Asian | 82 | 60 | 253 | 107 | NA | NA | NA | NA | NA | NA |
| Lu QG[197] 2013 | Asian | 12 | 30 | 68 | 70 | NA | NA | NA | NA | NA | NA |
| Pan[169] 2014 | Asian | 116 | 164 | 299 | 224 | 135 | 145 | 311 | 212 | NA | NA |
| Tao J[204] 2014 | Asian | NA | NA | NA | NA | NA | NA | NA | NA | 21 | 32 |
| Wang[173] 2015 | Asian | NA | NA | NA | NA | NA | NA | NA | NA | 27 | 43 |
| Sharma[171] 2015 | Indian | 63 | 52 | 167 | 103 | 102 | 13 | 233 | 37 | 100 | 14 |
| Peddireddy[174] 2016 | Indian | 69 | 28 | 187 | 63 | 78 | 19 | 224 | 26 | NA | NA |
| Girdhar[176] 2016 | Indian | 69 | 63 | 198 | 122 | 116 | 16 | 263 | 57 | NA | NA |
| Wang J[209] 2016 | Asian | 24 | 51 | 78 | 72 | NA | NA | NA | NA | NA | NA |
| Adenocarcinoma | | | | | | | | | | | |
| Seidegård[1] 1986 | Mixed | 9 | 20 | 46 | 32 | NA | NA | NA | NA | NA | NA |
| Seidegård[2] 1990 | Mixed | 19 | 45 | 66 | 48 | NA | NA | NA | NA | NA | NA |
| Zhong[3] 1991 | Mixed | 16 | 40 | 131 | 94 | NA | NA | NA | NA | NA | NA |
| Hirvonen[6] 1993 | Mixed | 32 | 19 | 102 | 88 | NA | NA | NA | NA | NA | NA |
| Brockmöller[7] 1993 | Caucasian | 16 | 16 | 174 | 181 | NA | NA | NA | NA | NA | NA |
| Katoh[10] 1994 | Asian | 7 | 2 | 28 | 20 | NA | NA | NA | NA | NA | NA |
| London[13] 1995 | Mixed | 71 | 49 | 404 | 312 | NA | NA | NA | NA | NA | NA |
| Kihara[15] 1995 | Asian | 98 | 113 | 241 | 228 | NA | NA | NA | NA | NA | NA |
| Kawajiri[256] 1995 | Asian | 73 | 78 | 191 | 167 | NA | NA | NA | NA | NA | NA |
| Moreira[19] 1996 | Caucasian | 12 | 14 | 40 | 44 | NA | NA | NA | NA | NA | NA |
| el-Zein[22] 1997 | Mixed | 17 | 10 | 27 | 23 | 22 | 5 | 43 | 7 | NA | NA |

| | | | | | | | | | | | |
|---|---|---|---|---|---|---|---|---|---|---|---|
| To-Figueras[24] 1997 | Caucasian | 17 | 25 | 155 | 37 | 33 | 9 | 97 | 95 | NA | NA |
| To-Figueras[24] 1997 | Caucasian | 17 | 25 | 93 | 27 | 33 | 9 | 60 | 60 | NA | NA |
| Ryberg[28] 1997 | Caucasian | 20 | 21 | 179 | 163 | NA | NA | NA | NA | 17 | 20 |
| Sun[30] 1997 | Asian | 22 | 46 | 178 | 186 | NA | NA | NA | NA | NA | NA |
| Hong[32] 1998 | Asian | 12 | 16 | 30 | 33 | NA | NA | NA | NA | NA | NA |
| Le Marchand[34] 1998 | Mixed | 73 | 88 | 182 | 268 | NA | NA | NA | NA | NA | NA |
| Saarikoski[36] 1998 | Caucasian | 48 | 34 | 157 | 137 | 72 | 9 | 255 | 39 | NA | NA |
| Gao[39] 1999 | Asian | 6 | 20 | 29 | 30 | NA | NA | NA | NA | NA | NA |
| Gao[39] 1999 | Asian | 6 | 20 | 36 | 37 | NA | NA | NA | NA | NA | NA |
| To-Figueras[40] 1999 | Caucasian | NA | NA | NA | NA | NA | NA | NA | NA | 21 | 15 |
| Kihara[43] 1999 | Asian | 67 | 93 | 126 | 131 | NA | NA | NA | NA | 121 | 32 |
| Woodson[261] 1999 | Caucasian | 29 | 24 | 171 | 162 | NA | NA | NA | NA | NA | NA |
| Butkiewicz[259] 1999 | Caucasian | 20 | 25 | 174 | 151 | NA | NA | NA | NA | 22 | 16 |
| Ford[47] 2000 | African | 31 | 12 | 96 | 24 | NA | NA | NA | NA | NA | NA |
| Malats[49] 2000 | Caucasian | 34 | 31 | 68 | 53 | 51 | 14 | 77 | 44 | NA | NA |
| Hou[51] 2000 | Caucasian | 29 | 25 | 194 | 181 | NA | NA | NA | NA | NA | NA |
| Liu[56] 2001 | Mixed | 215 | 257 | 475 | 561 | 412 | 100 | 918 | 258 | NA | NA |
| Risch[57] 2001 | Caucasian | 73 | 77 | 161 | 185 | 128 | 22 | 281 | 65 | 76 | 77 |
| Gsur[63] 2001 | Caucasian | 37 | 39 | 68 | 88 | NA | NA | NA | NA | NA | NA |
| Stücker[68] 2002 | Caucasian | 21 | 36 | 136 | 118 | 52 | 8 | 216 | 52 | 32 | 23 |
| Lewis[69] 2002 | Caucasian | 1 | 9 | 68 | 75 | 2 | 8 | 115 | 28 | 4 | 5 |
| Sunaga[70] 2002 | Asian | 105 | 93 | 96 | 56 | 99 | 99 | 93 | 59 | NA | NA |
| Lu[72] 2002 | Asian | 67 | 70 | 159 | 161 | NA | NA | NA | NA | NA | NA |
| Zhang JK[296] 2002 | Asian | 23 | 48 | 73 | 92 | 36 | 35 | 72 | 93 | NA | NA |
| Nie LH[249] 2002 | Asian | NA | NA | NA | NA | NA | NA | NA | NA | 41 | 28 |
| Reszka[73] 2003 | Caucasian | 7 | 5 | 101 | 64 | NA | NA | NA | NA | 6 | 6[a] |
| Wang[75] 2003 | Asian | 43 | 69 | 59 | 60 | 59 | 53 | 65 | 54 | 67 | 44 |
| Pinarbasi[76] 2003 | Caucasian | 7 | 4 | 169 | 37 | NA | NA | NA | NA | NA | NA |
| Wang[80] 2003 | Asian | 67 | 97 | 91 | 90 | NA | NA | NA | NA | NA | NA |
| Nazar-Stewart[81] 2003 | Mixed | 40 | 56 | 255 | 246 | 77 | 19 | 410 | 90 | 41 | 41 |
| Hung[82] 2003 | Caucasian | 70 | 68 | 590 | 600 | NA | NA | NA | NA | NA | NA |
| Lin[84] 2003 | Asian | NA | NA | NA | NA | NA | NA | NA | NA | 69 | 36 |
| Wang S[281] 2003 | Asian | 14 | 24 | 38 | 33 | NA | NA | NA | NA | NA | NA |
| Chan-Yeung[95] 2004 | Asian | 58 | 69 | 80 | 117 | 45 | 82 | 95 | 102 | 84 | 43 |
| Alexandrie[98] 2004 | Caucasian | 62 | 82 | 240 | 290 | 128 | 16 | 456 | 74 | NA | NA |
| Belogubova[99] 2004 | Caucasian | 21 | 18 | 333 | 330 | 31 | 8 | 333 | 330 | NA | NA |
| Schneider[100] 2004 | Caucasian | 59 | 53 | 328 | 294 | 97 | 15 | 507 | 115 | 48 | 43 |
| Sørensen[101] 2004 | Caucasian | NA | NA | NA | NA | 70 | 13 | 233 | 16 | NA | NA |
| Wang N[188] 2004 | Asian | 12 | 12 | 62 | 45 | 13 | 11 | 53 | 54 | NA | NA |
| Huang XH[227] 2004 | Asian | 11 | 20 | 65 | 73 | NA | NA | NA | NA | NA | NA |
| Li Y[282] 2004 | Asian | 5 | 8 | 77 | 61 | NA | NA | NA | NA | NA | NA |
| Raimondi[107] 2005 | Caucasian | 119 | 117 | 781 | 818 | 130 | 30 | 642 | 154 | NA | NA |
| Yuan TZ[205] 2005 | Asian | NA | NA | NA | NA | 31 | 30 | 94 | 58 | NA | NA |
| Li DR[260] 2005 | Asian | 29 | 32 | 91 | 61 | NA | NA | NA | NA | NA | NA |
| Liang GY[277] 2005 | Asian | 55 | 78 | 103 | 124 | 69 | 64 | 140 | 87 | 77 | 51 |
| Guo ZL[278] 2005 | Asian | NA | NA | NA | NA | NA | NA | NA | NA | 34 | 27 |
| Lee[113] 2006 | Asian | 22 | 26 | 91 | 105 | 26 | 22 | 89 | 107 | NA | NA |
| Larsen[117] 2006 | Caucasian | 231 | 267 | 258 | 367 | 377 | 117 | 510 | 114 | 225 | 22 |
| Miller[118] 2006 | Caucasian | NA | NA | NA | NA | NA | NA | NA | NA | 402 | 34 |
| Zhang TY[215] 2006 | Asian | NA | NA | NA | NA | NA | NA | NA | NA | 25 | 19 |
| Osawa[120] 2007 | Asian | 34 | 34 | 59 | 62 | NA | NA | NA | NA | NA | NA |
| Sobti[127] 2008 | Indian | 5 | 6 | 98 | 53 | 9 | 2 | 131 | 20 | 4 | 7[a] |
| Yoon[131] 2008 | Asian | NA | NA | NA | NA | NA | NA | NA | NA | 110 | 54 |
| Sreeja[132] 2008 | Indian | 42 | 19 | 147 | 64 | 45 | 16 | 183 | 28 | 35 | 20 |
| Chen H[275] 2009 | Asian | 12 | 24 | 208 | 247 | NA | NA | NA | NA | NA | NA |
| Liu JN[276] 2009 | Asian | NA | NA | NA | NA | 17 | 23 | 79 | 56 | NA | NA |

| | | | | | | | | | | | |
|---|---|---|---|---|---|---|---|---|---|---|---|
| Gervasini[142] 2010 | Caucasian | 10 | 6 | 127 | 120 | 14 | 2 | 206 | 41 | 10 | 6[a] |
| Altinisik[145] 2010 | Caucasian | 13 | 8 | 40 | 15 | 15 | 6 | 46 | 9 | NA | NA |
| Zheng DJ[287] 2010 | Asian | 51 | 48 | 132 | 175 | NA | NA | NA | NA | NA | NA |
| Hua F[279] 2010 | Asian | NA | NA | NA | NA | NA | NA | NA | NA | 65 | 31 |
| Young[150] 2011 | Caucasian | 70 | 118 | 465 | 680 | NA | NA | NA | NA | NA | NA |
| Zhou XL[271] 2011 | Asian | 32 | 37 | 119 | 89 | NA | NA | NA | NA | NA | NA |
| Fan J[272] 2011 | Asian | 8 | 27 | 31 | 39 | 11 | 24 | 36 | 34 | NA | NA |
| Du GB[290] 2011 | Asian | 14 | 17 | 54 | 71 | 17 | 14 | 69 | 56 | NA | NA |
| Dzian[156] 2012 | Caucasian | 54 | 64 | 130 | 160 | 83 | 35 | 242 | 48 | 59 | 46 |
| Ada[157] 2012 | Caucasian | 28 | 31 | 107 | 124 | 41 | 18 | 188 | 43 | 40 | 19 |
| López-Cima[159] 2012 | Caucasian | 109 | 126 | 358 | 418 | 193 | 42 | 611 | 165 | 115 | 12 |
| Liu[160] 2012 | Asian | 89 | 51 | 253 | 107 | NA | NA | NA | NA | NA | NA |
| Lu QG[197] 2013 | Asian | 18 | 31 | 68 | 70 | NA | NA | NA | NA | NA | NA |
| Pan[169] 2014 | Asian | 102 | 141 | 299 | 224 | 118 | 125 | 311 | 212 | NA | NA |
| Tao J[204] 2014 | Asian | NA | NA | NA | NA | NA | NA | NA | NA | 37 | 47 |
| Wang[173] 2015 | Asian | NA | NA | NA | NA | NA | NA | NA | NA | 34 | 27 |
| Sharma[171] 2015 | Indian | 39 | 47 | 167 | 103 | 62 | 24 | 233 | 37 | 74 | 11 |
| Peddireddy[174] 2016 | Indian | 80 | 29 | 187 | 63 | 88 | 21 | 224 | 26 | NA | NA |
| Girdhar[176] 2016 | Indian | 51 | 55 | 198 | 122 | 78 | 28 | 263 | 57 | NA | NA |
| Wang J[209] 2016 | Asian | 6 | 16 | 78 | 72 | NA | NA | NA | NA | NA | NA |

NA = not available, [a] = IIe/Val + Val/Val

**Supplemental Table 6 Genotype frequencies of the *GSTM1, GSTT1, and GSTP1* Ile105Val polymorphisms between and lung cancer and control groups by smoking status**

| First author/Year | Ethnicity | *GSTM1* genotype distribution | | | | *GSTT1* genotype distribution | | | | *GSTP1* Ile1[illegible] | |
|---|---|---|---|---|---|---|---|---|---|---|---|
| | | Case | | Control | | Case | | Control | | Case | |
| | | present | null | present | null | present | null | present | null | Ile/Ile | Il[illegible] |
| Smoking | | | | | | | | | | | |
| Seidegård[1] 1986 | Mixed | 23 | 43 | 46 | 32 | NA | NA | NA | NA | NA | [illegible] |
| Seidegård[2] 1990 | Mixed | 47 | 78 | 66 | 48 | NA | NA | NA | NA | NA | [illegible] |
| Hirvonen[6] 1993 | Mixed | 59 | 69 | 13 | 11 | NA | NA | NA | NA | NA | [illegible] |
| Brockmöller[7] 1993 | Caucasian | 51 | 60 | 61 | 74 | NA | NA | NA | NA | NA | [illegible] |
| Nazar-Stewart[9] 1993 | Mixed | 9 | 26 | 23 | 20 | NA | NA | NA | NA | NA | [illegible] |
| Katoh[10] 1994 | Asian | 23 | 20 | 28 | 20 | NA | NA | NA | NA | NA | [illegible] |
| London[13] 1995 | Mixed | 194 | 131 | 270 | 198 | NA | NA | NA | NA | NA | [illegible] |
| Kihara[15] 1995 | Asian | 141 | 197 | 120 | 112 | NA | NA | NA | NA | NA | [illegible] |
| Deakin[21] 1996 | Caucasian | 56 | 50 | 58 | 70 | 91 | 17 | 111 | 18 | NA | [illegible] |
| el-Zein[22] 1997 | Mixed | 31 | 23 | 27 | 23 | 42 | 12 | 43 | 7 | NA | [illegible] |
| Jourenkova[23] 1997 | Caucasian | 69 | 81 | 82 | 90 | 123 | 27 | 145 | 27 | NA | [illegible] |
| Kelsey[26] 1997 | Mixed | 108 | 52 | 107 | 54 | 125 | 35 | 134 | 27 | NA | [illegible] |
| Sun[30] 1997 | Asian | 42 | 98 | 84 | 89 | NA | NA | NA | NA | NA | [illegible] |
| Nyberg[35] 1998 | Caucasian | 53 | 43 | 44 | 38 | NA | NA | NA | NA | NA | [illegible] |
| Cheng TJ[257] 1995 | Mixed | 30 | 37 | 21 | 34 | NA | NA | NA | NA | NA | [illegible] |
| Gao[39] 1999 | Asian | 18 | 20 | 28 | 19 | NA | NA | NA | NA | NA | [illegible] |
| Katoh[42] 1999 | Asian | NA | NA | NA | NA | NA | NA | NA | NA | 30 | [illegible] |
| Kihara[43] 1999 | Asian | 152 | 206 | 95 | 89 | NA | NA | NA | NA | 263 | [illegible] |
| Woodson[261] 1999 | Caucasian | 159 | 160 | 171 | 162 | NA | NA | NA | NA | NA | [illegible] |
| Ford[47] 2000 | African | 73 | 33 | 69 | 22 | NA | NA | NA | NA | NA | [illegible] |
| Stücker[48] 2000 | Caucasian | 86 | 93 | 93 | 73 | NA | NA | NA | NA | NA | [illegible] |
| Spitz[52] 2000 | Caucasian | 227 | 214 | 219 | 204 | 313 | 115 | 322 | 97 | NA | [illegible] |
| Zhao[59] 2001 | Asian | 35 | 61 | 8 | 10 | 39 | 57 | 10 | 8 | NA | [illegible] |
| Chen SQ[60] 2001 | Asian | 38 | 42 | 51 | 29 | NA | NA | NA | NA | NA | [illegible] |
| Hou[61] 2001 | Caucasian | 38 | 24 | 24 | 27 | NA | NA | NA | NA | NA | [illegible] |
| Hou[64] 2001 | Caucasian | NA | NA | NA | NA | 83 | 13 | 77 | 6 | NA | [illegible] |
| Perera[67] 2002 | Caucasian | 31 | 40 | 75 | 62 | NA | NA | NA | NA | 27 | [illegible] |
| Sunaga[70] 2002 | Asian | 69 | 51 | 57 | 40 | 60 | 60 | 60 | 37 | NA | [illegible] |
| Zhang[123] 2002 | Asian | 18 | 33 | 19 | 13 | NA | NA | NA | NA | NA | [illegible] |
| Zhang JK[296] 2002 | Asian | 49 | 55 | 25 | 40 | 57 | 47 | 37 | 28 | NA | [illegible] |
| Nie LH[249] 2002 | Asian | NA | NA | NA | NA | NA | NA | NA | NA | 59 | [illegible] |
| Wang[75] 2003 | Asian | NA | NA | NA | NA | 25 | 23 | 21 | 27 | 30 | [illegible] |
| Wang[80] 2003 | Asian | 26 | 44 | 38 | 38 | NA | NA | NA | NA | NA | [illegible] |
| Nazar-Stewart[81] 2003 | Mixed | 127 | 136 | 190 | 171 | 212 | 51 | 296 | 65 | 101 | [illegible] |
| Taioli[83] 2003 | Caucasian | 92 | 104 | 301 | 297 | 86 | 32 | 291 | 69 | NA | [illegible] |
| Lin[84] 2003 | Asian | NA | NA | NA | NA | NA | NA | NA | NA | 72 | [illegible] |
| Wang[85] 2003 | Caucasian | NA | NA | NA | NA | NA | NA | NA | NA | 134 | [illegible] |
| Ruano-Ravina[88] 2003 | Caucasian | 52 | 63 | 58 | 52 | 91 | 24 | 85 | 25 | NA | [illegible] |
| Xian XZ[224] 2003 | Asian | 24 | 31 | 28 | 34 | NA | NA | NA | NA | NA | [illegible] |
| Chen LJ[232] 2003 | Asian | 9 | 16 | 15 | 21 | NA | NA | NA | NA | NA | [illegible] |
| Wang S[281] 2003 | Asian | 21 | 46 | 9 | 25 | NA | NA | NA | NA | NA | [illegible] |
| Wang[90] 2004 | Caucasian | 293 | 381 | 271 | 336 | 540 | 129 | 470 | 133 | NA | [illegible] |
| Habalová[93] 2004 | Caucasian | 47 | 46 | 40 | 48 | NA | NA | NA | NA | NA | [illegible] |
| Alexandrie[98] 2004 | Caucasian | 102 | 94 | 130 | 142 | 172 | 24 | 231 | 41 | NA | [illegible] |
| Belogubova[99] 2004 | Caucasian | 69 | 79 | 187 | 186 | 125 | 23 | 306 | 67 | NA | [illegible] |
| Schneider[100] 2004 | Caucasian | 7 | 11 | 74 | 77 | 15 | 3 | 125 | 26 | 178 | [illegible] |
| Gallegos-Arreola[102] 2003-2004 | Mixed | NA | NA | NA | NA | 39 | 9 | 70 | 4 | NA | [illegible] |
| Li WY[29] 2004 | Asian | 49 | 72 | 40 | 25 | NA | NA | NA | NA | NA | [illegible] |
| Gu YF[210] 2004 | Asian | 42 | 54 | 32 | 27 | NA | NA | NA | NA | NA | [illegible] |
| Huang XH[227] 2004 | Asian | 24 | 31 | 28 | 34 | NA | NA | NA | NA | NA | [illegible] |
| Li Y[282] 2004 | Asian | 33 | 43 | 41 | 33 | NA | NA | NA | NA | NA | [illegible] |
| Sreeja[104] 2005 | Indian | 83 | 36 | 61 | 24 | 90 | 29 | 75 | 10 | NA | [illegible] |
| Adonis[106] 2005 | Mixed | 21 | 13 | 34 | 16 | NA | NA | NA | NA | NA | [illegible] |
| Cote[110] 2005 | Caucasian | 103 | 103 | 80 | 73 | 152 | 43 | 124 | 25 | 71 | [illegible] |
| Brennan[111] 2005 | Caucasian | 962 | 947 | 709 | 666 | 1,636 | 316 | 1,181 | 230 | NA | [illegible] |
| Li DR[192] 2005 | Asian | 28 | 35 | 6 | 10 | NA | NA | NA | NA | NA | [illegible] |
| Qiao GB[201] 2005 | Asian | 67 | 108 | 63 | 55 | NA | NA | NA | NA | NA | [illegible] |
| Yuan TZ[205] 2005 | Asian | NA | NA | NA | NA | 28 | 70 | 33 | 19 | NA | [illegible] |
| Luo CL[216] 2005 | Asian | 14 | 28 | 12 | 22 | NA | NA | NA | NA | NA | [illegible] |
| Li DR[260] 2005 | Asian | 46 | 52 | 25 | 27 | NA | NA | NA | NA | NA | [illegible] |
| Liang GY[277] 2005 | Asian | NA | NA | NA | NA | NA | NA | NA | NA | 69 | [illegible] |
| Guo ZL[278] 2005 | Asian | NA | NA | NA | NA | NA | NA | NA | NA | 49 | [illegible] |

| | | | | | | | | | | | |
|---|---|---|---|---|---|---|---|---|---|---|---|
| Miller[118] 2006 | Caucasian | NA | NA | NA | NA | NA | NA | NA | NA | 621 | 7[illegible] |
| Qian[65] 2006 | Asian | 31 | 54 | 31 | 31 | NA | NA | NA | NA | NA | N[illegible] |
| Chang FH[126] 2006 | Asian | 39 | 62 | 51 | 16 | NA | NA | NA | NA | NA | N[illegible] |
| Wang QM[184] 2006 | Asian | 7 | 30 | 11 | 12 | NA | NA | NA | NA | NA | N[illegible] |
| Zhang TY[215] 2006 | Asian | NA | NA | NA | NA | NA | NA | NA | NA | 29 | 3[illegible] |
| Osawa[120] 2007 | Asian | 38 | 41 | 29 | 32 | NA | NA | NA | NA | NA | N[illegible] |
| Hu XG[274] 2007 | Asian | 21 | 49 | 33 | 23 | NA | NA | NA | NA | NA | N[illegible] |
| Wang YS[266] 2007 | Asian | 30 | 43 | 21 | 33 | NA | NA | NA | NA | NA | N[illegible] |
| Chen H[180] 2008 | Asian | 46 | 73 | 88 | 120 | NA | NA | NA | NA | NA | N[illegible] |
| Qi XS[220] 2008 | Asian | NA | NA | NA | NA | 30 | 17 | 36 | 23 | NA | N[illegible] |
| Xia Y[246] 2008 | Asian | 21 | 31 | 44 | 48 | NA | NA | NA | NA | NA | N[illegible] |
| Hou Y[273] 2008 | Asian | 25 | 37 | 17 | 31 | NA | NA | NA | NA | NA | N[illegible] |
| Lam[137] 2009 | Mixed | 56 | 69 | 194 | 199 | 96 | 29 | 298 | 79 | NA | N[illegible] |
| Kumar[139] 2009 | Indian | 42 | 39 | 65 | 36 | 62 | 19 | 79 | 22 | NA | N[illegible] |
| Qi XS[219] 2009 | Asian | 18 | 29 | 26 | 33 | NA | NA | NA | NA | NA | N[illegible] |
| Yin Q[247] 2009 | Asian | NA | NA | NA | NA | NA | NA | NA | NA | 38 | 3[illegible] |
| Chen H[275] 2009 | Asian | 46 | 73 | 88 | 120 | NA | NA | NA | NA | NA | N[illegible] |
| Liu JN[276] 2009 | Asian | NA | NA | NA | NA | 18 | 31 | 32 | 18 | NA | N[illegible] |
| Jin[141] 2010 | Asian | 43 | 70 | 36 | 51 | NA | NA | NA | NA | NA | N[illegible] |
| Gervasini[142] 2010 | Caucasian | 53 | 44 | 92 | 98 | 82 | 15 | 160 | 30 | 51 | 4[illegible] |
| Timofeeva[143] 2010 | Caucasian | 262 | 309 | 398 | 410 | 476 | 94 | 667 | 140 | 258 | 2[illegible] |
| Cabral[144] 2010 | Mixed | 20 | 35 | 6 | 3 | 34 | 21 | 8 | 1 | NA | N[illegible] |
| Altinisik[145] 2010 | Caucasian | 40 | 27 | 11 | 8 | 53 | 15 | 15 | 4 | NA | N[illegible] |
| Song B[234] 2010 | Asian | 35 | 52 | 36 | 29 | NA | NA | NA | NA | NA | N[illegible] |
| Zheng DJ[287] 2010 | Asian | 65 | 108 | 63 | 78 | NA | NA | NA | NA | NA | N[illegible] |
| Jia SH[245] 2010 | Asian | NA | NA | NA | NA | 18 | 31 | 32 | 18 | NA | N[illegible] |
| Young[150] 2011 | Caucasian | 157 | 252 | 99 | 122 | NA | NA | NA | NA | NA | N[illegible] |
| Ihsan[152] 2011 | Indian | 91 | 41 | 86 | 53 | 106 | 26 | 104 | 35 | 69 | 5[illegible] |
| Bai TY[207] 2011 | Asian | NA | NA | NA | NA | 32 | 30 | 76 | 40 | NA | N[illegible] |
| Zhang JQ[226] 2011 | Asian | 7 | 13 | 21 | 13 | NA | NA | NA | NA | NA | N[illegible] |
| Zhou XL[271] 2011 | Asian | 44 | 86 | 43 | 34 | NA | NA | NA | NA | NA | N[illegible] |
| Fan J[272] 2011 | Asian | 11 | 22 | 11 | 18 | 9 | 22 | 16 | 11 | NA | N[illegible] |
| Bai TY[289] 2011 | Asian | 23 | 60 | 64 | 35 | 47 | 36 | 55 | 44 | 42 | 4[illegible] |
| Du GB[290] 2011 | Asian | 35 | 41 | 20 | 23 | 43 | 33 | 25 | 18 | NA | N[illegible] |
| López-Cima[159] 2012 | Caucasian | 349 | 376 | 255 | 299 | 578 | 147 | 425 | 129 | 330 | 4[illegible] |
| Liu[160] 2012 | Asian | 152 | 103 | 130 | 55 | NA | NA | NA | NA | NA | N[illegible] |
| Han RL[199] 2012 | Asian | 23 | 60 | 64 | 35 | NA | NA | NA | NA | NA | N[illegible] |
| Yao ZG[200] 2012 | Asian | 38 | 67 | 42 | 30 | 16 | 29 | 40 | 38 | NA | N[illegible] |
| Chen CM[288] 2012 | Asian | 57 | 89 | 50 | 63 | NA | NA | NA | NA | NA | N[illegible] |
| Shukla[163] 2013 | Indian | 102 | 47 | 42 | 26 | 85 | 64 | 53 | 15 | NA | N[illegible] |
| Lv XL[183] 2013 | Asian | NA | NA | NA | NA | NA | NA | NA | NA | 38 | 3[illegible] |
| Lu QG[197] 2013 | Asian | 22 | 33 | 30 | 32 | NA | NA | NA | NA | NA | N[illegible] |
| Pan[169] 2014 | Asian | 104 | 135 | 137 | 102 | 117 | 122 | 140 | 99 | NA | N[illegible] |
| Jiang[170] 2014 | Asian | 115 | 75 | 65 | 151 | 131 | 94 | 109 | 90 | 42 | 1[illegible] |
| Wang[173] 2015 | Asian | NA | NA | NA | NA | NA | NA | NA | NA | 49 | 4[illegible] |
| Peddireddy[174] 2016 | Indian | 106 | 42 | 54 | 21 | 111 | 37 | 66 | 9 | NA | N[illegible] |
| Liu AS[191] 2017 | Asian | 20 | 29 | 13 | 18 | NA | NA | NA | NA | NA | N[illegible] |
| He[182] 2018 | Asian | 66 | 48 | 50 | 28 | NA | NA | NA | NA | NA | N[illegible] |
| No-smoking | | | | | | | | | | | |
| Brockmöller[7] 1993 | Caucasian | 4 | 2 | 12 | 8 | NA | NA | NA | NA | NA | N[illegible] |
| Hirvonen[6] 1993 | Mixed | 6 | 1 | 6 | 5 | NA | NA | NA | NA | NA | N[illegible] |
| London[13] 1995 | Mixed | 8 | 7 | 128 | 112 | NA | NA | NA | NA | NA | N[illegible] |
| Kihara[15] 1995 | Asian | 52 | 50 | 121 | 116 | NA | NA | NA | NA | NA | N[illegible] |
| Kelsey[26] 1997 | Mixed | 3 | 5 | 82 | 35 | 6 | 2 | 98 | 19 | NA | N[illegible] |
| Sun[30] 1997 | Asian | 18 | 49 | 94 | 97 | NA | NA | NA | NA | NA | N[illegible] |
| Nyberg[35] 1998 | Caucasian | 47 | 41 | 36 | 43 | NA | NA | NA | NA | NA | N[illegible] |
| Cheng TJ[257] 1995 | Mixed | 2 | 5 | 14 | 8 | NA | NA | NA | NA | NA | N[illegible] |
| Gao[39] 1999 | Asian | 7 | 14 | 39 | 46 | NA | NA | NA | NA | NA | N[illegible] |
| Katoh[42] 1999 | Asian | NA | NA | NA | NA | NA | NA | NA | NA | 4 | 0[a] |
| Kihara[43] 1999 | Asian | 12 | 12 | 31 | 42 | NA | NA | NA | NA | 15 | 8 |
| Ford[47] 2000 | African | 4 | 3 | 22 | 2 | NA | NA | NA | NA | NA | N[illegible] |
| Malats[49] 2000 | Caucasian | 56 | 66 | 68 | 53 | 90 | 32 | 77 | 44 | NA | N[illegible] |
| Zhao[59] 2001 | Asian | 52 | 85 | 60 | 109 | 62 | 75 | 75 | 94 | NA | N[illegible] |
| Chen[60] 2001 | Asian | 10 | 16 | 16 | 10 | NA | NA | NA | NA | NA | N[illegible] |
| Hou[61] 2001 | Caucasian | 55 | 53 | 46 | 47 | NA | NA | NA | NA | NA | N[illegible] |
| Gsur[63] 2001 | Caucasian | 35 | 28 | 30 | 33 | NA | NA | NA | NA | NA | N[illegible] |
| Hou[64] 2001 | Caucasian | NA | NA | NA | NA | 84 | 4 | 66 | 13 | NA | N[illegible] |
| Perera[67] 2002 | Caucasian | 7 | 9 | 18 | 13 | NA | NA | NA | NA | 7 | 8[a] |

| | | | | | | | | | | |
|---|---|---|---|---|---|---|---|---|---|---|
| Sunaga[70] 2002 | Asian | 35 | 40 | 36 | 13 | 36 | 39 | 29 | 20 | NA | N |
| Zhang LZ[123]] 2002 | Asian | 6 | 8 | 14 | 14 | NA | NA | NA | NA | NA | N |
| Zhang JK[296] 2002 | Asian | 18 | 39 | 48 | 52 | 30 | 27 | 56 | 44 | NA | N |
| Nie LH[249] 2002 | Asian | NA | NA | NA | NA | NA | NA | NA | NA | 30 | 2[illegible] |
| Wang[75] 2003 | Asian | NA | NA | NA | NA | 34 | 30 | 44 | 27 | 37 | 2[illegible] |
| Kiyohara[79] 2003 | Asian | 64 | 4 | 124 | 135 | NA | NA | NA | NA | NA | N |
| Wang[80] 2003 | Asian | 41 | 53 | 53 | 52 | NA | NA | NA | NA | NA | N |
| Nazar-Stewart[81] 2003 | Mixed | 4 | 7 | 65 | 74 | 10 | 1 | 114 | 24 | 6 | 5[a] |
| Taioli[83] 2003 | Caucasian | 14 | 18 | 288 | 285 | 16 | 5 | 323 | 58 | NA | N |
| Lin[84] 2003 | Asian | NA | NA | NA | NA | NA | NA | NA | NA | 52 | 3[illegible] |
| Wang[85] 2003 | Caucasian | NA | NA | NA | NA | NA | NA | NA | NA | 15 | 2[illegible] |
| Ruano-Ravina[88] 2003 | Caucasian | 1 | 9 | 42 | 35 | 7 | 3 | 56 | 21 | NA | N |
| Xian XZ[224] 2003 | Asian | 11 | 25 | 37 | 39 | NA | NA | NA | NA | NA | N |
| Chen LJ[232] 2003 | Asian | 5 | 8 | 27 | 36 | NA | NA | NA | NA | NA | N |
| Wang S[281] 2003 | Asian | 14 | 15 | 29 | 8 | NA | NA | NA | NA | NA | N |
| Wang[90] 2004 | Caucasian | 19 | 23 | 152 | 180 | 33 | 9 | 280 | 52 | NA | N |
| Habalová[93] 2004 | Caucasian | 6 | 5 | 29 | 33 | NA | NA | NA | NA | NA | N |
| Alexandrie[98] 2004 | Caucasian | 13 | 18 | 99 | 133 | 26 | 5 | 200 | 32 | NA | N |
| Belogubova[99] 2004 | Caucasian | 7 | 12 | 146 | 144 | 12 | 7 | 232 | 58 | NA | N |
| Schneider[100] 2004 | Caucasian | 191 | 207 | 253 | 216 | 330 | 68 | 380 | 89 | 13 | 5[a] |
| Gallegos-Arreola[102] 2003-2004 | Mixed | NA | NA | NA | NA | 2 | 2 | 99 | 5 | NA | N |
| Li[29] 2004 | Asian | 41 | 55 | 65 | 70 | NA | NA | NA | NA | NA | N |
| Gu YF[210]] 2004 | Asian | 28 | 35 | 60 | 48 | NA | NA | NA | NA | NA | N |
| Huang XH[227] 2004 | Asian | 11 | 25 | 37 | 39 | NA | NA | NA | NA | NA | N |
| Li Y[282] 2004 | Asian | 7 | 20 | 36 | 28 | NA | NA | NA | NA | NA | N |
| Sreeja[104] 2005 | Indian | 17 | 10 | 46 | 15 | 24 | 3 | 58 | 3 | NA | N |
| Adonis[106] 2005 | Mixed | 12 | 9 | 33 | 20 | NA | NA | NA | NA | NA | N |
| Wenzlaff[109] 2005 | Caucasian | 62 | 69 | 70 | 77 | 100 | 24 | 115 | 30 | 39 | 5[illegible] |
| Wenzlaff[109] 2005 | African | 20 | 9 | 22 | 8 | 25 | 4 | 23 | 7 | 8 | 1[illegible] |
| Brennan[111] 2005 | Caucasian | 81 | 75 | 388 | 320 | 139 | 24 | 615 | 114 | NA | N |
| Li DR[192] 2005 | Asian | 14 | 22 | 33 | 17 | NA | NA | NA | NA | NA | N |
| Qiao GB[201] 2005 | Asian | 17 | 21 | 41 | 40 | NA | NA | NA | NA | NA | N |
| Yuan TZ[205] 2005 | Asian | NA | NA | NA | NA | 40 | 12 | 61 | 39 | NA | N |
| Luo CL[216] 2005 | Asian | 4 | 17 | 18 | 40 | NA | NA | NA | NA | NA | N |
| Li DR[260] 2005 | Asian | 29 | 23 | 62 | 38 | NA | NA | NA | NA | NA | N |
| Liang GY[277] 2005 | Asian | NA | NA | NA | NA | NA | NA | NA | NA | 66 | 3[illegible] |
| Guo ZL[278] 2005 | Asian | NA | NA | NA | NA | NA | NA | NA | NA | 25 | 2[illegible] |
| Miller[118] 2006 | Caucasian | NA | NA | NA | NA | NA | NA | NA | NA | 64 | 6[illegible] |
| Qian[65] 2006 | Asian | 8 | 15 | 24 | 22 | NA | NA | NA | NA | NA | N |
| Chang FH[126] 2006 | Asian | 18 | 44 | 34 | 62 | NA | NA | NA | NA | NA | N |
| Wang QM[184] 2006 | Asian | 9 | 10 | 12 | 7 | NA | NA | NA | NA | NA | N |
| Zhang TY[215]] 2006 | Asian | NA | NA | NA | NA | NA | NA | NA | NA | 30 | 3[illegible] |
| Osawa[120] 2007 | Asian | 17 | 15 | 26 | 29 | NA | NA | NA | NA | NA | N |
| Hu XG[274] 2007 | Asian | 8 | 34 | 20 | 28 | NA | NA | NA | NA | NA | N |
| Wang YS[266] 2007 | Asian | 8 | 13 | 17 | 23 | NA | NA | NA | NA | NA | N |
| Yoon[131] 2008 | Asian | NA | NA | NA | NA | NA | NA | NA | NA | 137 | 6[illegible] |
| Chen H[180] 2008 | Asian | 13 | 26 | 120 | 126 | NA | NA | NA | NA | NA | N |
| Qi XS[220] 2008 | Asian | NA | NA | NA | NA | 5 | 0 | 9 | 4 | NA | N |
| Hou Y[273] 2008 | Asian | 7 | 8 | 12 | 17 | NA | NA | NA | NA | NA | N |
| Lam[137] 2009 | Mixed | 6 | 6 | 25 | 23 | 10 | 0 | 37 | 8 | NA | N |
| Kumar[139] 2009 | Indian | 7 | 5 | 89 | 63 | 7 | 5 | 118 | 34 | NA | N |
| Qi XS[219] 2009 | Asian | 1 | 4 | 5 | 8 | NA | NA | NA | NA | NA | N |
| Yin Q[247] 2009 | Asian | NA | NA | NA | NA | NA | NA | NA | NA | 24 | 1[illegible] |
| Chen H[275] 2009 | Asian | 13 | 26 | 120 | 126 | NA | NA | NA | NA | NA | N |
| Liu JN[276] 2009 | Asian | NA | NA | NA | NA | 25 | 26 | 47 | 38 | NA | N |
| Jin[141] 2010 | Asian | 12 | 25 | 35 | 28 | NA | NA | NA | NA | NA | N |
| Cabral[144] 2010 | Mixed | 4 | 5 | 34 | 44 | 4 | 5 | 37 | 41 | NA | N |
| Altinisik[145] 2010 | Caucasian | 6 | 1 | 29 | 7 | 6 | 1 | 31 | 5 | NA | N |
| Song B[234] 2010 | Asian | 16 | 22 | 34 | 26 | NA | NA | NA | NA | NA | N |
| Zheng DJ[287] 2010 | Asian | 49 | 36 | 68 | 96 | NA | NA | NA | NA | NA | N |
| Zhu XX[291] 2010 | Asian | 67 | 93 | 88 | 72 | NA | NA | NA | NA | NA | N |
| Jia SH[245] 2010 | Asian | NA | NA | NA | NA | 25 | 26 | 47 | 38 | NA | N |
| Ihsan[152] 2011 | Indian | 31 | 25 | 91 | 60 | 49 | 7 | 113 | 38 | 33 | 2[illegible] |
| Fowke[154] 2011 | Asian | 98 | 110 | 329 | 456 | 100 | 108 | 403 | 381 | NA | N |
| Bai TY[207] 2011 | Asian | NA | NA | NA | NA | 24 | 20 | 63 | 71 | NA | N |
| Zhang JQ[226] 2011 | Asian | 9 | 15 | 13 | 9 | NA | NA | NA | NA | NA | N |
| Zhou XL[271] 2011 | Asian | 43 | 36 | 76 | 55 | NA | NA | NA | NA | NA | N |
| Fan J[272] 2011 | Asian | 10 | 25 | 20 | 21 | 14 | 23 | 19 | 24 | NA | N |

| | | | | | | | | | | | |
|---|---|---|---|---|---|---|---|---|---|---|---|
| Bai TY[289] 2011 | Asian | 19 | 26 | 61 | 54 | 25 | 20 | 63 | 52 | 28 | [illegible] |
| Du GB[290] 2011 | Asian | 17 | 32 | 36 | 46 | 22 | 27 | 45 | 37 | NA | NA |
| López-Cima[159] 2012 | Caucasian | 26 | 25 | 102 | 119 | 40 | 11 | 185 | 36 | 22 | [illegible] |
| Liu[160]] 2012 | Asian | 63 | 42 | 123 | 52 | NA | NA | NA | NA | NA | NA |
| Han RL[199] 2012 | Asian | 26 | 19 | 61 | 54 | NA | NA | NA | NA | NA | NA |
| Yao ZG[200] 2012 | Asian | 16 | 29 | 40 | 38 | NA | NA | NA | NA | NA | NA |
| Chen CM[288] 2012 | Asian | 20 | 34 | 29 | 47 | NA | NA | NA | NA | NA | NA |
| Shukla[163] 2013 | Indian | 32 | 37 | 106 | 64 | 51 | 18 | 127 | 43 | NA | NA |
| Lv XL[183] 2013 | Asian | NA | NA | NA | NA | NA | NA | NA | NA | 24 | [illegible] |
| Lu QG[197]] 2013 | Asian | 8 | 28 | 38 | 38 | NA | NA | NA | NA | NA | NA |
| Pan[169] 2014 | Asian | 114 | 170 | 162 | 122 | 136 | 148 | 171 | 113 | NA | NA |
| Jiang[170] 2014 | Asian | 130 | 136 | 48 | 58 | 120 | 111 | 73 | 50 | 51 | [illegible] |
| Wang[173] 2015 | Asian | NA | NA | NA | NA | NA | NA | NA | NA | 25 | [illegible] |
| Peddireddy[174] 2016 | Indian | 76 | 22 | 133 | 42 | 89 | 9 | 158 | 17 | NA | NA |
| Liu AS[191] 2017 | Asian | 7 | 15 | 32 | 8 | NA | NA | NA | NA | NA | NA |
| He[182]] 2018 | Asian | 113 | 86 | 167 | 85 | NA | NA | NA | NA | NA | NA |

NA = not available, [a] = IIe/Val + Val/Val

**Supplemental Table 7 Genotype frequencies of the *GSTM1, GSTT1, and GSTP1* IIe105Val polymorphisms between and lung cancer and control groups by gender**

| First author/Year | Ethnicity | *GSTM1* genotype distribution | | | | *GSTT1* genotype distribution | | | | *GSTP1* IIe105V | |
|---|---|---|---|---|---|---|---|---|---|---|---|
| | | Case | | Control | | Case | | Control | | Case | |
| | | present | null | present | null | present | null | present | null | IIe/IIe | IIe/V |
| Male | | | | | | | | | | | |
| Seidegård[2] 1990 | Mixed | 25 | 45 | 27 | 27 | NA | NA | NA | NA | NA | NA |
| Brockmöller[7] 1993 | Caucasian | 43 | 46 | 108 | 105 | NA | NA | NA | NA | NA | NA |
| London[13] 1995 | Mixed | 111 | 89 | 270 | 205 | NA | NA | NA | NA | NA | NA |
| Kihara[15] 1995 | Asian | 150 | 185 | 150 | 153 | NA | NA | NA | NA | NA | NA |
| Kelsey[26] 1997 | Mixed | 80 | 46 | 127 | 65 | 102 | 24 | 164 | 28 | NA | NA |
| Ryberg[28] 1997 | Caucasian | 61 | 74 | 179 | 163 | NA | NA | NA | NA | 53 | 63 |
| Sun[30] 1997 | Asian | 38 | 101 | 129 | 139 | NA | NA | NA | NA | NA | NA |
| Kihara[43] 1999 | Asian | 152 | 206 | 126 | 131 | NA | NA | NA | NA | 263 | 78 |
| Woodson[261] 1999 | Caucasian | 159 | 160 | 171 | 162 | NA | NA | NA | NA | NA | NA |
| Kiyohara[44] 2000 | Asian | 33 | 53 | 39 | 49 | 39 | 47 | 49 | 39 | 61 | 23 |
| Dresler[45] 2000 | Mixed | 34 | 53 | 24 | 35 | NA | NA | NA | NA | NA | NA |
| London[46] 2000 | Asian | 110 | 122 | 283 | 427 | 98 | 134 | 284 | 426 | NA | NA |
| Hou[51] 2000 | Caucasian | 132 | 150 | 194 | 181 | NA | NA | NA | NA | NA | NA |
| Risch[57] 2001 | Caucasian | 105 | 126 | 99 | 131 | 200 | 31 | 182 | 48 | 105 | 107 |
| Perera[67] 2002 | Caucasian | 39 | 47 | 91 | 69 | NA | NA | NA | NA | 33 | 37 |
| Stücker[68] 2002 | Caucasian | 118 | 129 | 136 | 118 | 213 | 38 | 216 | 52 | 120 | 101 |
| Lewis[69] 2002 | Caucasian | 37 | 19 | 37 | 44 | 46 | 10 | 66 | 15 | 21 | 34 |
| Zhang JK[296] 2002 | Asian | 53 | 66 | 48 | 62 | 64 | 55 | 59 | 51 | NA | NA |
| Nazar-Stewart[81] 2003 | Mixed | 131 | 143 | 255 | 246 | 222 | 52 | 410 | 90 | 106 | 118 |
| Lin[84] 2003 | Asian | NA | NA | NA | NA | NA | NA | NA | NA | 89 | 54[a] |
| Wang[85] 2003 | Caucasian | NA | NA | NA | NA | NA | NA | NA | NA | 74 | 106[a] |
| Habalová[93] 2004 | Caucasian | 44 | 59 | 53 | 61 | NA | NA | NA | NA | NA | NA |
| Belogubova[99] 2004 | Caucasian | 70 | 78 | 156 | 147 | 124 | 24 | 248 | 55 | NA | NA |
| Chan[105] 2005 | Asian | 38 | 24 | 59 | 80 | NA | NA | NA | NA | NA | NA |
| Liang[112] 2005 | Asian | NA | NA | NA | NA | NA | NA | NA | NA | 98 | 70[a] |
| Lee[113] 2006 | Asian | 71 | 98 | 91 | 105 | 89 | 80 | 89 | 107 | NA | NA |
| Larsen[117] 2006 | Caucasian | 366 | 420 | 178 | 247 | 617 | 165 | 346 | 78 | 355 | 360 |
| Miller[118] 2006 | Caucasian | NA | NA | NA | NA | NA | NA | NA | NA | 448 | 427 |
| Sørensen[122] 2007 | Caucasian | 90 | 137 | 204 | 220 | 191 | 36 | 367 | 56 | 110 | 93 |
| Zupa[136] 2009 | Caucasian | 23 | 40 | 21 | 35 | NA | NA | NA | NA | NA | NA |
| Lam[137] 2009 | Mixed | 28 | 39 | 106 | 98 | 54 | 13 | 150 | 54 | NA | NA |
| Liu JN[276] 2009 | Asian | NA | NA | NA | NA | 23 | 34 | 43 | 31 | NA | NA |
| Timofeeva[143] 2010 | Caucasian | 185 | 208 | 397 | 382 | 327 | 66 | 647 | 129 | 187 | 163 |
| Cabral[144] 2010 | Mixed | 18 | 27 | 19 | 28 | 23 | 22 | 20 | 25 | NA | NA |
| Song B[234] 2010 | Asian | 51 | 74 | 70 | 55 | NA | NA | NA | NA | NA | NA |
| Young[150] 2011 | Caucasian | 83 | 141 | 130 | 185 | NA | NA | NA | NA | NA | NA |
| Singh[151] 2010-2011 | Indian | 129 | 71 | 147 | 53 | NA | NA | NA | NA | NA | NA |
| Du GB[290] 2011 | Asian | 40 | 53 | 42 | 48 | 54 | 39 | 50 | 40 | NA | NA |
| Han RL[199] 2012 | Asian | 49 | 79 | 125 | 89 | NA | NA | NA | NA | NA | NA |
| Piao[165] 2013 | Asian | 1331 | 1792 | 369 | 451 | 1469 | 1654 | 412 | 408 | NA | NA |
| Pan[169] 2014 | Asian | 122 | 143 | 144 | 121 | 93 | 172 | 167 | 98 | NA | NA |
| Mota[172] 2015 | Caucasian | 79 | 58 | 78 | 66 | 91 | 46 | 113 | 31 | 59 | 69 |
| Minina[181] 2017 | Caucasian | 210 | 143 | 172 | 128 | 267 | 86 | 233 | 67 | 135 | 180 |
| He[182] 2018 | Asian | 134 | 93 | 141 | 84 | NA | NA | NA | NA | NA | NA |
| Female | | | | | | | | | | | |
| Seidegård[2] 1990 | Mixed | 22 | 33 | 39 | 21 | NA | NA | NA | NA | NA | NA |
| Brockmöller[7] 1993 | Caucasian | 12 | 16 | 66 | 76 | NA | NA | NA | NA | NA | NA |
| London[13] 1995 | Mixed | 93 | 49 | 134 | 107 | NA | NA | NA | NA | NA | NA |
| Kihara[15] 1995 | Asian | 47 | 65 | 91 | 75 | NA | NA | NA | NA | NA | NA |
| Kelsey[26] 1997 | Mixed | 31 | 11 | 62 | 24 | 29 | 13 | 68 | 18 | NA | NA |
| Sun[30] 1997 | Asian | 22 | 46 | 49 | 47 | NA | NA | NA | NA | NA | NA |
| Qu YH[211] 1998 | Asian | 80 | 102 | 85 | 94 | NA | NA | NA | NA | NA | NA |
| Dresler[45] 2000 | Mixed | 32 | 50 | 42 | 63 | NA | NA | NA | NA | NA | NA |
| Risch[57] 2001 | Caucasian | 28 | 35 | 35 | 30 | 57 | 6 | 52 | 13 | 27 | 31 |
| Lewis[69] 2002 | Caucasian | 19 | 12 | 31 | 31 | 22 | 9 | 49 | 13 | 13 | 19 |
| Zhang JK[296] 2002 | Asian | 14 | 28 | 25 | 30 | 23 | 19 | 34 | 21 | NA | NA |
| Kiyohara[79] 2003 | Asian | 64 | 4 | 124 | 135 | NA | NA | NA | NA | NA | NA |
| Lin[84] 2003 | Asian | NA | NA | NA | NA | NA | NA | NA | NA | 35 | 20[a] |
| Wang[85] 2003 | Caucasian | NA | NA | NA | NA | NA | NA | NA | NA | 75 | 107[a] |
| Habalová[93] 2004 | Caucasian | 9 | 9 | 16 | 20 | NA | NA | NA | NA | NA | NA |
| Yang[94] 2004 | Asian | 78 | 108 | 64 | 75 | NA | NA | NA | NA | NA | NA |
| Belogubova[99] 2004 | Caucasian | 6 | 13 | 177 | 56 | 13 | 6 | 290 | 70 | NA | NA |

| | | | | | | | | | | | |
|---|---|---|---|---|---|---|---|---|---|---|---|
| Chan[105] 2005 | Asian | 6 | 7 | 12 | 11 | NA | NA | NA | NA | NA | NA |
| Liang[112] 2005 | Asian | NA | NA | NA | NA | NA | NA | NA | NA | 37 | 22 [a] |
| Larsen[117] 2006 | Caucasian | 137 | 171 | 80 | 120 | 245 | 61 | 164 | 36 | 146 | 124 |
| Miller[118] 2006 | Caucasian | NA | NA | NA | NA | NA | NA | NA | NA | 437 | 389 |
| Sørensen[122] 2007 | Caucasian | 96 | 105 | 168 | 172 | 171 | 31 | 288 | 52 | 84 | 87 |
| Yoon[131] 2008 | Asian | NA | NA | NA | NA | NA | NA | NA | NA | 137 | 65 |
| Zupa[136] 2009 | Caucasian | 9 | 3 | 31 | 34 | NA | NA | NA | NA | NA | NA |
| Lam[137] 2009 | Mixed | 34 | 42 | 113 | 130 | 52 | 24 | 185 | 58 | NA | NA |
| Cote[138] 2009 | Caucasian | 178 | 210 | 206 | 197 | 311 | 77 | 324 | 82 | 171 | 177 |
| Cote[138] 2009 | African | 78 | 36 | 93 | 28 | 87 | 28 | 95 | 26 | 28 | 61 |
| Liu JN[276] 2009 | Asian | NA | NA | NA | NA | 20 | 23 | 36 | 25 | NA | NA |
| Timofeeva[143] 2010 | Caucasian | 94 | 126 | 210 | 262 | 184 | 35 | 388 | 85 | 92 | 106 |
| Cabral[144] 2010 | Mixed | 6 | 13 | 18 | 22 | 15 | 4 | 24 | 16 | NA | NA |
| Zhu XX[291] 2010 | Asian | 67 | 93 | 88 | 72 | NA | NA | NA | NA | NA | NA |
| Young[150] 2011 | Caucasian | 85 | 129 | 83 | 150 | NA | NA | NA | NA | NA | NA |
| Fowke[154] 2011 | Asian | 98 | 110 | 329 | 456 | 100 | 108 | 403 | 381 | NA | NA |
| Du GB[290] 2011 | Asian | 12 | 20 | 13 | 23 | 14 | 18 | 20 | 16 | NA | NA |
| Piao[165] 2013 | Asian | 365 | 445 | 407 | 472 | 394 | 416 | 429 | 450 | NA | NA |
| Pan[169]] 2014 | Asian | 96 | 162 | 155 | 103 | 160 | 98 | 144 | 114 | NA | NA |
| Mota[172] 2015 | Caucasian | 29 | 17 | 49 | 35 | 31 | 15 | 61 | 23 | 12 | 25 |
| He[182] 2018 | Asian | 45 | 41 | 76 | 29 | NA | NA | NA | NA | NA | NA |

NA = not available, [a] = IIe/Val + Val/Val

**Supplemental Table 8 Genotype frequencies of the combined effects of *GSTM1* present/null and *GSTT1* present/null between lung cancer and control groups**

| First author/Year | Country | Ethnicity | SC | Genotype | | | | | | | |
|---|---|---|---|---|---|---|---|---|---|---|---|
| | | | | A | | B | | C | | D | |
| | | | | Cases | Controls | Cases | Controls | Cases | Controls | Cases | Cc |
| Sharma[171] 2015 | India | Indian | HB | 27 | 22 | 23 | 15 | 111 | 88 | 109 | 14 |
| Zhang[221] 2014 | China | Asian | HB | 24 | 35 | 51 | 28 | 15 | 24 | 20 | 23 |
| Pan[169] 2014 | China | Asian | PB | 113 | 121 | 157 | 91 | 148 | 133 | 105 | 17 |
| Piao[165] 2013 | Korea | Asian | PB | 873 | 391 | 1197 | 467 | 1040 | 456 | 823 | 38 |
| Dzian[156] 2012 | Slovak | Caucasian | HB | 31 | 26 | 28 | 22 | 102 | 138 | 69 | 10 |
| Ada[157] 2012 | Turkey | Caucasian | HB | NA | NA | 34 | 22 | NA | NA | 73 | 86 |
| López-Cima[159] 2012 | Australia | Caucasian | HB | 71 | 82 | 87 | 83 | 327 | 363 | 304 | 27 |
| Liang KC[242] 2012 | China | Asian | HB | NA | NA | 34 | 19 | NA | NA | NA | N/ |
| Cabral[144] 2010 | Brazil | Mixed | HB | 6 | 14 | 20 | 27 | 19 | 23 | 19 | 23 |
| Fan J[272] 2011 | China | Asian | HB | 18 | 14 | 34 | 19 | 15 | 16 | 11 | 17 |
| Du GB[290] 2011 | China | Asian | HB | NA | NA | 36 | 30 | NA | NA | 31 | 28 |
| Matakova[133] 2009 | Slovak | Caucasian | HB | 21 | 20 | 20 | 26 | 74 | 79 | 45 | 95 |
| Sreeja[132] 2008 | India | Indian | HB | NA | NA | 29 | 10 | NA | NA | 102 | 12 |
| Sobti[127] 2008 | India | Indian | HB | 15 | 13 | 12 | 7 | 51 | 46 | 73 | 85 |
| Qi XS[220] 2008 | China | Asian | HB | 7 | 10 | 10 | 17 | 24 | 24 | 12 | 21 |
| Honma[128] 2008 | Brazil | Mixed | HB | NA | NA | 12 | 13 | NA | NA | 94 | 11 |
| Li SF[268] 2007 | China | Asian | HB | 7 | 18 | 10 | 30 | 14 | 27 | 11 | 28 |
| Chen[114] 2006 | China | Asian | PB | 23 | 41 | 36 | 44 | 24 | 45 | 14 | 67 |
| Yao W[189]] 2006 | China | Asian | HB | 18 | 29 | 26 | 25 | 19 | 20 | 14 | 33 |
| Cote[110] 2005 | USA | Caucasian | PB | NA | NA | 87 | 124 | NA | NA | 19 | 29 |
| Cote[110] 2005 | USA | African | PB | NA | NA | 53 | 67 | NA | NA | 8 | 6 |
| Wenzlaff[109] 2005 | USA | Caucasian | PB | NA | NA | 48 | 56 | NA | NA | 12 | 17 |
| Wenzlaff[109] 2005 | USA | African | PB | NA | NA | 16 | 14 | NA | NA | 1 | 0 |
| Brennan[111] 2005 | Poland | Caucasian | PB | NA | NA | 175 | 171 | NA | NA | 861 | 92 |
| Sørensen[101]] 2004 | Denmark | Caucasian | PB | 17 | 9 | 20 | 7 | 120 | 154 | 97 | 95 |
| Cao YF[252] 2004 | China | Asian | HB | 26 | 41 | 43 | 46 | 22 | 49 | 13 | 69 |
| Wang[90] 2004 | USA | Caucasian | HB | NA | NA | 77 | 112 | NA | NA | NA | N/ |
| Vineis[91] 2004 | Multiple | Caucasian | ND | 169 | 266 | 196 | 276 | 856 | 1139 | 746 | 10 |
| Wang J[75] 2003 | China | Asian | HB | 17 | 25 | 36 | 29 | 33 | 31 | 26 | 34 |
| Cajas-Salazar[74]] 2003 | USA | Caucasian | HB | NA | NA | 16 | 9 | NA | NA | 47 | 65 |
| Ruano-Ravina[88] 2003 | Spain | Caucasian | HB | 12 | 18 | 15 | 28 | 57 | 59 | 41 | 82 |
| Dialyna[77] 2003 | Greece | Caucasian | HB | 12 | 7 | 9 | 13 | 54 | 83 | 47 | 75 |
| Stücker[68] 2002 | France | Caucasian | HB | NA | NA | 111 | 98 | NA | NA | 25 | 19 |
| Zhang JK[193] 2002 | China | Asian | HB | 7 | 11 | 12 | 10 | 15 | 20 | 7 | 14 |
| Zhao[59] 2001 | China | Asian | HB | NA | NA | 82 | 66 | NA | NA | NA | N/ |
| London[46] 2000 | China | Asian | PB | NA | NA | 85 | 275 | NA | NA | NA | N/ |
| Malats[49] 2000 | Multiple | Caucasian | HB | 15 | 24 | 17 | 20 | 49 | 33 | 41 | 44 |
| Spitz[52] 2000 | USA | Caucasian | HB | NA | NA | 54 | 42 | NA | NA | NA | N/ |
| Salagovic[31] 1998 | Slovak | Caucasian | PB | NA | NA | 10 | 17 | NA | NA | NA | N/ |
| Saarikoski[36] 1998 | Finland | Caucasian | PB | 23 | 10 | 16 | 15 | 82 | 122 | 96 | 13 |
| To-Figueras[24] 1997 | Spain | Caucasian | HB+PB | 16 | 32 | 23 | 32 | 70 | 123 | 51 | 12 |
| El-Zein[22] 1997 | USA | Mixed | HB | NA | NA | 6 | 2 | NA | NA | NA | N/ |
| Jourenkova[23] 1997 | France | Caucasian | HB | NA | NA | 15 | 12 | NA | NA | NA | N/ |
| Kelsey[26] 1997 | USA | African | HB | NA | NA | 9 | 8 | NA | NA | NA | N/ |
| Kelsey[26] 1997 | USA | Mixed | HB | NA | NA | 5 | 5 | NA | NA | NA | N/ |

NA = not available, [a] = Ile/Val + Val/Val, A = M1 *GSTM1* present/*GSTT1* null, B = *GSTM1* null/*GSTT1* null, C = *GSTM1* null/*GSTT1* present, D = *GSTM1* present/*GSTT1* present, E = *GSTM1* present/*GSTT1* null + *GSTM1* null/*GSTT1* present, F = *GSTM1* present/*GSTT1* present + *GSTM1* present/*GSTT1* null + *GSTM1* null/*GSTT1* present

**Supplemental Table 9 Genotype frequencies of the combined effects of *GSTM1* present/null and *GSTP1* IIe105Val polymorphism between lung cancer and control groups**

| First author/Year | Ethnicity | *GSTM1* present/ *GSTP1* IIe/IIe | | *GSTM1* null/ *GSTP1* IIe/IIe | | *GSTM1* present/*GSTP1* Val [1] | | Total one risk genotype | |
|---|---|---|---|---|---|---|---|---|---|
| | | Case | Control | Case | Control | Case | Control | Case | Control |
| Sharma[171] 2015 | Indian | 109 | 148 | 116 | 85 | 27 | 19 | 143 | 104 |
| López-Cima[159] 2012 | Caucasian | 161 | 146 | 184 | 175 | 212 | 209 | 396 | 384 |
| Dzian[156] 2012 | Caucasian | 51 | 69 | 64 | 84 | 49 | 61 | 113 | 145 |
| Ada[157] 2012 | Caucasian | 61 | 63 | NA | NA | NA | NA | 101 | 114 |
| Matakova[133]] 2009 | Caucasian | 35 | 66 | 46 | 60 | 31 | 49 | 77 | 109 |
| Honma[128] 2008 | Mixed | 47 | 50 | 35 | 55 | 62 | 87 | 97 | 142 |
| Sreeja[132] 2008 | Indian | 73 | 86 | 45 | 49 | 66 | 61 | 111 | 110 |
| Sobti[127] 2008 | Indian | 50 | 40 | 28 | 22 | 38 | 58 | 66 | 80 |
| Chen[114] 2006 | Asian | 26 | 74 | 40 | 69 | 11 | 34 | 51 | 103 |
| Wenzlaff[109] 2005 | Caucasian | 16 | 30 | NA | NA | NA | NA | 55 | 69 |
| Wenzlaff[109] 2005 | African | 5 | 5 | NA | NA | NA | NA | 14 | 17 |
| Cote[110] 2005 | Caucasian | 40 | 54 | NA | NA | NA | NA | 118 | 150 |
| Cote[110] 2005 | African | 10 | 29 | NA | NA | NA | NA | 58 | 65 |
| Reszka[73] 2003 | Caucasian | 48 | 47 | NA | NA | NA | NA | 62 | 90 |
| Wang[75] 2003 | Asian | 27 | 45 | 40 | 39 | 16 | 14 | 56 | 53 |
| Perera[67] 2002 | Caucasian | 13 | 43 | 21 | 39 | 26 | 48 | 47 | 87 |
| Stücker[68] 2002 | Caucasian | 99 | 124 | NA | NA | NA | NA | 133 | 112 |
| Miller[71] 2002 | Caucasian | 158 | 185 | 195 | 221 | 186 | 238 | 381 | 459 |
| To-Figueras[40] 1999 | Caucasian | 37 | 73 | 46 | 81 | 31 | 94 | 77 | 175 |
| Jourenkova-Mironova[37] 1998 | Caucasian | 31 | 36 | 35 | 48 | 35 | 45 | 70 | 93 |
| Ryberg[28] 1997 | Caucasian | 25 | 81 | 24 | 72 | 35 | 76 | 59 | 148 |

Val [1] = IIe/Val + Val/Val, Total one risk genotype = *GSTM1* null/ *GSTP1* IIe/IIe + *GSTM1* present/*GSTP1* Val [1], All risk genotypes = *GSTM1* null/ *GSTP1* IIe/IIe + *GSTM1* present/*GSTP1* Val [1] + *GSTM1* null/ *GSTP1* Val [1], NA = not available

**Supplemental Table 10 Genotype frequencies of the combined effects of *GSTT1* present/null and *GSTP1* IIe105Val between lung cancer and control groups**

| First author/Year | Ethnicity | *GSTT1* present/ *GSTP1* IIe/IIe | | *GSTT1* null/ *GSTP1* IIe/IIe | | *GSTT1* present/ *GSTP1* Val [1] | | Total one risk genotype | |
|---|---|---|---|---|---|---|---|---|---|
| | | Case | Control | Case | Control | Case | Control | Case | Control |
| Sharma[171] 2015 | Indian | 184 | 201 | 39 | 32 | 36 | 32 | 75 | 64 |
| Dzian[156] 2012 | Caucasian | 85 | 123 | 30 | 30 | 86 | 119 | 116 | 149 |
| López-Cima[159] 2012 | Caucasian | 276 | 256 | 69 | 65 | 341 | 352 | 410 | 417 |
| Ada[157] 2012 | Caucasian | 105 | 106 | NA | NA | NA | NA | 85 | 109 |
| Matakova[133]] 2009 | Caucasian | 60 | 104 | 21 | 22 | 59 | 70 | 80 | 92 |
| Sreeja[132] 2008 | Indian | 86 | 117 | 32 | 17 | 59 | 66 | 91 | 83 |
| Honma[128]] 2008 | Mixed | 71 | 93 | 11 | 12 | 102 | 137 | 113 | 149 |
| Sobti[127] 2008 | Indian | 66 | 57 | 12 | 5 | 58 | 74 | 70 | 79 |
| Chen[114] 2006 | Asian | 31 | 79 | 35 | 64 | 7 | 33 | 42 | 97 |
| Wenzlaff[109] 2005 | Caucasian | 26 | 46 | NA | NA | NA | NA | 63 | 80 |
| Wenzlaff[109] 2005 | African | 7 | 5 | NA | NA | NA | NA | 15 | 19 |
| Cote[110] 2005 | Caucasian | 58 | 82 | NA | NA | NA | NA | 124 | 171 |
| Cote[110] 2005 | African | 12 | 28 | NA | NA | NA | NA | 61 | 72 |
| Sørensen[101] 2004 | Caucasian | 107 | 107 | 10 | 8 | 110 | 142 | 120 | 150 |
| Wang[80] 2003 | Asian | 32 | 45 | 35 | 39 | 27 | 20 | 62 | 59 |
| Stücker[68] 2002 | Caucasian | 188 | 197 | NA | NA | NA | NA | 58 | 62 |
| To-Figueras[40] 1999 | Caucasian | 63 | 117 | 20 | 37 | 60 | 144 | 80 | 181 |

NA = not available, Val[1] = IIe/Val + Val/Val, Total one risk genotype = *GSTT1* null/ *GSTP1* IIe/IIe + *GSTT1* present/ *GSTP1* Val [1], All risk genotypes = *GSTT1* null/ *GSTP1* IIe/IIe + *GSTT1* present/ *GSTP1* Val [1] + *GSTT1* null/*GSTP1* Val [1]

**Supplemental Table 11** Genotype frequencies of the combined effects of *GSTM1*, *GSTT1* and *GSTP1* IIe105Val polymorphisms between lung cancer and control groups

| First author/Year | A | | B | | C | | D | | E | | F | | G | | [illegible] |
|---|---|---|---|---|---|---|---|---|---|---|---|---|---|---|---|
| | Case | Control | Case | Control | Case | Control | Case | Control | Case | Control | Case | Control | Case | Control | [illegible] |
| Sharma[171] 2015 | 87 | 128 | NA | NA | NA | NA | NA | NA | NA | NA | NA | NA | NA | NA | [illegible] |
| Dzian[156] 2012 | 33 | 50 | 52 | 73 | 18 | 19 | 36 | 54 | 106 | 146 | 12 | 11 | 50 | 65 | [illegible] |
| Ada[157] 2012 | 51 | 50 | NA | NA | NA | NA | NA | NA | NA | NA | NA | NA | NA | NA | [illegible] |
| Sørensen[122] 2007 | 139 | 277 | NA | NA | NA | NA | NA | NA | 218 | 382 | NA | NA | NA | NA | [illegible] |
| Chen[114] 2006 | 13 | 44 | NA | NA | NA | NA | NA | NA | 32 | 88 | NA | NA | NA | NA | [illegible] |
| Cote[110] 2005 | 28 | 42 | NA | NA | NA | NA | NA | NA | 97 | 134 | NA | NA | NA | NA | [illegible] |
| Cote[110] 2005 | 9 | 20 | NA | NA | NA | NA | NA | NA | 47 | 62 | NA | NA | NA | NA | [illegible] |

NA = not available, A = *M1* Present/*T1* Present/*P1* IIe/IIe, B = *M1* Null/*T1* Present/*P1* IIe/IIe, C = *M1* Present/*T1* Null/*P1* IIe/IIe, D = *M1* Present/*T1* Present/*P1* Val [1], E = *M1* Null/*T1* Present/*P1* IIe/IIe + *M1* Present/*T1* Null/*P1* IIe/IIe + *M1* Present/*T1* Present/*P1* Val [1], F = *M1* Null/*T1* Null/*P1* IIe/IIe, G = *M1* Null/*T1* Present/*P1* Val [1], H = *M1* Present/*T1* Null/*P1* Val [1], I = *M1* Null/*T1* Null/*P1* IIe/IIe + *M1* Null/*T1* Present/*P1* Val [1] + *M1* Present/*T1* Null/*P1* Val [1], J = *M1* Null/*T1* Null/*P1* Val[1], Val[1]: IIe/Val + Val/Val

**Supplemental Table 12** Scale for quality assessment of molecular association studies of lung cancer

| Criterion | Score |
|---|---|
| Source of case | |
| Selected from population or cancer registry | 3 |
| Selected from hospital | 2 |
| Selected from pathology archives, but without description | 1 |
| Not described | 0 |
| Source of control | |
| Population-based | 3 |
| Blood donors or volunteers | 2 |
| Hospital-based | 1 |
| Not described | 0 |
| Ascertainment of cancer | |
| Histological or pathological confirmation | 2 |
| Diagnosis of lung cancer by patient medical record | 1 |
| Not described | 0 |
| Ascertainment of control | |
| Controls were tested to screen out lung cancer | 2 |
| Controls were subjects who did not report lung cancer, no objective testing | 1 |
| Not described | 0 |
| Matching | |
| Controls matched with cases by age and sex | 2 |
| Controls matched with cases only by age or sex | 1 |
| Not matched or not described | 0 |
| Genotyping examination | |
| Genotyping done blindly and quality control | 2 |
| Only genotyping done blindly or quality control | 1 |
| Unblinded and without quality control | 0 |
| Specimens used for determining genotypes | |
| Blood cells or normal tissues | 1 |
| Tumor tissues or exfoliated cells of tissue | 0 |
| HWE | |
| HWE in the control group | 1 |
| Hardy-Weinberg disequilibrium in the control group | 0 |
| Association assessment | |
| Assess association between genotypes and lung cancer with appropriate statistics and adjustment for confounders | 2 |
| Assess association between genotypes and lung cancer with appropriate statistics without adjustment for confounders | 1 |
| Inappropriate statistics used | 0 |
| Total sample size | |
| >1000 | 3 |
| 500-1000 | 2 |

| | |
|---|---|
| 200-500 | 1 |
| <200 | 0 |

HWE: Hardy-Weinberg equilibrium

**Supplemental Table 13** Meta-analysis of the association of *GSTM1* polymorphism with risk of lung cancer

| Variable | n | Cases/Controls | Test of association | Test of heterogeneity | | Model |
|---|---|---|---|---|---|---|
| | | | OR (95% CI) | $P_h$ | $I^2$ (%) | |
| Overall | 205 | 45,726/58,788 | **1.24 (1.19–1.30)** | <0.001 | 58.5 | Random-effect |
| Ethnicity | | | | | | |
| African | 8 | 698/916 | 1.20 (0.96–1.50) | 0.739 | 0.0 | Fixed-effect |
| Indian | 16 | 2,998/3,360 | 1.17 (0.93–1.46) | <0.001 | 76.6 | Random-effect |
| Asian | 95 | 18,240/19,218 | **1.43 (1.33–1.53)** | <0.001 | 54.8 | Random-effect |
| Caucasian | 66 | 19,961/30,395 | **1.07 (1.01–1.13)** | 0.001 | 39.4 | Random-effect |
| Country | | | | | | |
| China | 74 | 10,271/13,195 | **1.52 (1.40–1.65)** | <0.001 | 53.3 | Random-effect |
| Japan | 13 | 2,886/2,952 | **1.30 (1.17–1.44)** | 0.617 | 0.0 | Fixed-effect |
| Korea | 4 | 4,504/2,304 | 1.09 (0.99–1.21) | 0.712 | 0.0 | Fixed-effect |
| North India | 11 | 2,059/2,423 | 1.15 (0.84–1.58) | <0.001 | 83.8 | Random-effect |
| South India | 4 | 689/667 | 1.20 (0.95–1.53) | 0.686 | 0.0 | Fixed-effect |
| Source of control | | | | | | |
| HB | 116 | 21,670/25,884 | **1.30 (1.21–1.39)** | <0.001 | 64.0 | Random-effect |
| PB | 41 | 12,988/15,069 | **1.14 (1.05–1.24)** | <0.001 | 55.6 | Random-effect |
| Matching | | | | | | |
| Yes | 90 | 19,316/25,042 | **1.18 (1.10–1.25)** | <0.001 | 55.1 | Random-effect |
| No | 115 | 26,410/33,746 | **1.30 (1.23–1.39)** | <0.001 | 60.8 | Random-effect |
| Quality score | | | | | | |
| > 12 | 64 | 22,879/29,881 | **1.14 (1.07–1.21)** | <0.001 | 57.8 | Random-effect |
| ⩽12 | 141 | 22,847/28,907 | **1.31 (1.24–1.39)** | <0.001 | 56.9 | Random-effect |
| Sample size | | | | | | |
| > 200 | 154 | 42,466/54,925 | **1.21 (1.16–1.27)** | <0.001 | 63.2 | Random-effect |
| ⩽ 200 | 51 | 3,260/3,863 | **1.42 (1.29–1.57)** | 0.127 | 18.8 | Fixed-effect |
| Histological type | | | | | | |
| SCLC | 39 | 1,511/11,179 | **1.38 (1.16–1.63)** | <0.001 | 50.2 | Random-effect |
| SCLC/Asian | 11 | 364/2,477 | **1.43 (1.04–1.97)** | 0.062 | 43.3 | Random-effect |
| SCLC/Caucasian | 17 | 790/6005 | **1.33 (1.01–1.76)** | <0.001 | 65.7 | Random-effect |
| SCLC/Indian | 4 | 193/817 | **1.66 (1.21–2.28)** | 0.852 | 0.0 | Fixed-effect |
| LCLC | 8 | 108/2,403 | 1.23 (0.83–1.81) | 0.145 | 35.5 | Fixed-effect |
| SC | 75 | 7,008/21,733 | **1.33 (1.22–1.45)** | <0.001 | 55.2 | Random-effect |
| SC/Asian | 32 | 2,571/6,485 | **1.52 (1.38-1.66)** | 0.292 | 10.9 | Fixed-effect |
| SC/Caucasian | 27 | 2,952/10,513 | 1.16 (0.97–1.38) | <0.001 | 72.6 | Random-effect |
| SC/Indian | 6 | 711/1,267 | **1.37 (1.13–1.67)** | 0.956 | 0.0 | Fixed-effect |
| AC | 71 | 6,542/22,646 | **1.24 (1.13–1.36)** | <0.001 | 52.0 | Random-effect |
| AC/Asian | 29 | 2,490/6,192 | **1.35 (1.22–1.48)** | 0.107 | 25.5 | Fixed-effect |
| AC/Caucasian | 27 | 2,560/11,784 | 1.07 (0.94–1.22) | 0.009 | 43.5 | Random-effect |
| AC/Indian | 3 | 373/1,202 | **1.49 (1.17–1.90)** | 0.292 | 19.2 | Fixed-effect |

| | | | | | | |
|---|---|---|---|---|---|---|
| Smoking | | | | | | |
| Yes | 90 | 14,118/13,575 | **1.27 (1.17–1.39)** | <0.001 | 61.7 | Random-effect |
| No | 82 | 4,885/10,612 | **1.36 (1.21–1.53)** | <0.001 | 50.4 | Random-effect |
| Gender | | | | | | |
| Male | 39 | 10,409/10,390 | **1.16 (1.06–1.26)** | 0.001 | 47.1 | Random-effect |
| Female | 32 | 4,303/6,371 | 1.16 (0.98–1.39) | <0.001 | 72.3 | Random-effect |

HB = hospital-based studies, PB = population-based studies, SCLC = small-cell lung cancer, LCLC = large cell lung carcinoma, SC = squamous carcinoma, AC = adenocarcinoma

**Supplemental Table 14** Meta-analysis of the association of *GSTT1* polymorphism with risk of lung cancer

| Variable | n | Cases/Controls | Test of association | Test of heterogeneity | | Model |
|---|---|---|---|---|---|---|
| | | | OR (95% CI) | $P_h$ | $I^2$ (%) | |
| Overall | 103 | 29,476/35,305 | **1.16 (1.08–1.24)** | <0.001 | 59.2 | Random-effect |
| Ethnicity | | | | | | |
| African | 5 | 362/413 | 0.98 (0.70–1.37) | 0.564 | 0.0 | Fixed-effect |
| Indian | 15 | 2,624/2,993 | **1.54 (1.13–2.11)** | <0.001 | 78.5 | Random-effect |
| Asian | 33 | 9,442/8,865 | **1.23 (1.12–1.36)** | 0.001 | 49.1 | Random-effect |
| Caucasian | 41 | 14,782/19,972 | 1.05 (0.97–1.14) | 0.005 | 40.1 | Random-effect |
| Country | | | | | | |
| China | 24 | 3,766/5,535 | **1.31 (1.16–1.49)** | 0.004 | 48.9 | Random-effect |
| Japan | 4 | 938/822 | **1.22 (1.01–1.47)** | 0.352 | 8.2 | Fixed-effect |
| Korea | 3 | 4,418/2,240 | 1.08 (0.97–1.19) | 0.156 | 46.2 | Fixed-effect |
| North India | 4 | 689/667 | **2.99 (1.88–4.78)** | 0.101 | 51.8 | Random-effect |
| South India | 10 | 1,685/2,056 | 1.25 (0.90–1.75) | <0.001 | 75.0 | Random-effect |
| Source of control | | | | | | |
| HB | 53 | 12,703/14,711 | **1.17 (1.06–1.29)** | <0.001 | 63.1 | Random-effect |
| PB | 23 | 9,110/9,751 | 1.11 (0.99–1.24) | 0.008 | 46.3 | Random-effect |
| Matching | | | | | | |
| Yes | 47 | 11,595/15,282 | **1.12 (1.02–1.24)** | <0.001 | 56.3 | Random-effect |
| No | 56 | 17,881/20,023 | **1.19 (1.08–1.30)** | <0.001 | 61.9 | Random-effect |
| Quality score | | | | | | |
| > 12 | 42 | 16,562/19,771 | **1.11 (1.02–1.21)** | <0.001 | 54.8 | Random-effect |
| ≤12 | 61 | 12,914/15,534 | **1.20 (1.08–1.33)** | <0.001 | 62.0 | Random-effect |
| Sample size | | | | | | |
| > 200 | 88 | 28,568/34,347 | **1.15 (1.08–1.23)** | <0.001 | 60.7 | Random-effect |
| ≤ 200 | 15 | 908/958 | 1.20 (0.89–1.63) | 0.013 | 50.6 | Random-effect |
| Histological type | | | | | | |
| SCLC | 21 | 975/5,719 | 0.96 (0.80–1.14) | 0.103 | 29.2 | Fixed-effect |
| LCLC | 3 | 51/1,181 | **0.39 (0.17–0.94)** | 0.208 | 36.3 | Fixed-effect |
| SC | 37 | 3,832/11,426 | 1.13 (0.98–1.31) | <0.001 | 54.4 | Random-effect |
| SC/Asian | 11 | 790/1,969 | **1.38 (1.02–1.87)** | 0.002 | 63.5 | Random-effect |
| SC/Caucasian | 18 | 2,170/6,664 | 1.02 (0.85–1.23) | 0.024 | 44.0 | Random-effect |
| SC/Indian | 5 | 511/1,067 | 1.13 (0.72–1.78) | 0.064 | 54.1 | Random-effect |
| AC | 37 | 4,020/11,663 | 1.18 (0.99–1.39) | <0.001 | 68.6 | Random-effect |
| AC/Asian | 12 | 1,123/2,168 | **1.36 (1.17–1.58)** | 0.150 | 30.2 | Fixed-effect |
| AC/Caucasian | 17 | 1,889/6,567 | 0.96 (0.70–1.31) | <0.001 | 77.1 | Random-effect |
| AC/Indian | 5 | 373/1,202 | **2.02 (1.51–2.70)** | 0.865 | 0.0 | Fixed-effect |
| Smoking | | | | | | |
| Yes | 43 | 8,464/8,513 | **1.23 (1.08–1.40)** | <0.001 | 56.1 | Random-effect |
| No | 38 | 2,799/6,570 | 1.09 (0.94–1.25) | 0.055 | 28.4 | Random-effect |

| | | | | | | |
|---|---|---|---|---|---|---|
| Gender | | | | | | |
| Male | 21 | 7,234/6,243 | 1.10 (0.93–1.29) | <0.001 | 67.2 | Random-effect |
| Female | 18 | 2,919/4,553 | 1.04 (0.93–1.16) | 0.458 | 0.0 | Fixed-effect |

HB = hospital-based studies, PB = population-based studies, SCLC = small-cell lung cancer, LCLC = large cell lung carcinoma, SC = squamous carcinoma, AC = adenocarcinoma

**Supplemental Table 15** Meta-analysis of the association of *GSTP1* polymorphism with risk of lung cancer

| Variable | n (Cases/Controls) | Val/Val vs. IIe/IIe | | IIe/Val vs. IIe/IIe | | Val/Val vs. IIe/IIe + IIe/Val | | Val/Val |
|---|---|---|---|---|---|---|---|---|
| | | OR (95% CI) | $P_h/I^2$ | OR (95% CI) | $P_h/I^2$ | OR (95% CI) | $P_h/I^2$ | OR (95 |
| Overall | 69 (18,852/21,941) | 1.06 (0.98–1.14) | 0.116/18.3 | 1.05 (0.99–1.11) | 0.037/26.1 | 1.05 (0.98–1.13) | 0.208/12.6 | **1.06 (1.** |
| Ethnicity | | | | | | | | |
| African | 3 (232/268) | 1.22 (0.71–2.10) | 0.763/0.0 | 1.42 (0.93–2.17) | 0.210/35.9 | 0.97 (0.62–1.53) | 0.741/0.0 | 1.37 (0. |
| Asian | 23 (4,359/5,032) | **1.45 (1.16–1.80)** | 0.361/7.6 | **1.13 (1.02–1.24)** | 0.305/12.0 | **1.39 (1.12–1.72)** | 0.620/0.0 | **1.16 (1.** |
| Caucasian | 32 (12,148/13,968) | 1.00 (0.91–1.09) | 0.151/21.8 | 0.99 (0.94–1.05) | 0.161/21.0 | 1.00 (0.92–1.09) | 0.152/21.8 | 0.98 (0. |
| Indian | 5 (913/1,175) | 1.17 (0.76–1.80) | 0.197/33.6 | 1.05 (0.78–1.42) | 0.060/55.7 | 1.14 (0.74–1.75) | 0.309/16.5 | 1.14 (0. |
| Source of control | | | | | | | | |
| HB | 41 (11,475/11,549) | **1.12 (1.01–1.25)** | 0.279/11.5 | 1.07 (0.99–1.16) | 0.020/36.2 | **1.12 (1.01–1.24)** | 0.368/6.0 | **1.08 (1.** |
| PB | 16 (5,459/7,173) | 0.93 (0.83–1.06) | 0.278/15.7 | 1.00 (0.92–1.08) | 0.368/7.6 | 0.93 (0.83–1.04) | 0.317/12.2 | 0.98 (0. |
| Matching | | | | | | | | |
| Yes | 36 (9,330/11,950) | 1.06 (0.93–1.21) | 0.202/17.1 | 1.04 (0.97–1.12) | 0.156/20.6 | 1.01 (0.92–1.12) | 0.370/6.1 | 1.04 (0. |
| No | 33 (9,522/9,991) | 1.09 (0.98–1.22) | 0.157/21.0 | 1.06 (0.98–1.16) | 0.043/33.3 | 1.09 (0.98–1.20) | 0.174/19.7 | 1.07 (0. |
| Quality score | | | | | | | | |
| > 12 | 37 (13,546/15,202) | 1.00 (0.92–1.09) | 0.215/16.4 | 0.98 (0.93–1.04) | 0.263/13.1 | 1.01 (0.93–1.10) | 0.192/18.0 | 0.98 (0. |
| ≤12 | 32 (5,306/6,739) | **1.23 (1.06–1.42)** | 0.314/9.8 | **1.13 (1.05–1.23)** | 0.113/24.6 | **1.16 (1.01–1.34)** | 0.437/1.8 | **1.16 (1.** |
| Sample size | | | | | | | | |
| > 200 | 65 (18,601/21,594) | 1.09 (0.99–1.20) | 0.088/21.0 | 1.06 (0.99–1.12) | 0.021/29.9 | 1.05 (0.98–1.13) | 0.182/14.5 | **1.06 (1.** |
| ≤ 200 | 4 (251/347) | 0.88 (0.40–1.94) | 0.492/0.0 | 1.07 (0.74–1.55) | 0.721/0.0 | 0.93 (0.44–1.96) | 0.370/4.6 | 1.03 (0. |
| HWE | | | | | | | | |
| Yes | 63 (17,634/20,618) | **1.08 (1.00–1.17)** | 0.137/17.6 | 1.03 (0.98–1.08) | 0.146/17.0 | 1.08 (0.99–1.16) | 0.249/11.0 | 1.03 (0. |
| No | 6 (1,218/1,323) | **0.73 (0.54–0.99)** | 0.865/0.0 | 1.11 (0.80–1.53) | 0.007/68.6 | **0.71 (0.53–0.95)** | 0.986/0.0 | 1.03 (0. |
| Histological type | | | | | | | | |
| SCLC | 17 (1,113/6,012) | **1.34 (1.01–1.77)** | 0.601/0.0 | 1.07 (0.82–1.38) | 0.060/45.0 | **1.32 (1.01–1.72)** | 0.242/21.8 | 0.90 (0. |
| SCLC/Caucasian | 11 (756/4,423) | **1.42 (1.05–1.92)** | 0.718/0.0 | 1.00 (0.82–1.23) | 0.123/40.2 | **1.41 (1.07–1.87)** | 0.273/20.6 | 1.01 (0. |
| LCLC | 4 (193/2,544) | 0.74 (0.41–1.32) | 0.204/37.0 | 0.92 (0.66–1.27) | 0.393/0.0 | 0.77 (0.44–1.35) | 0.371/0.0 | 0.87 (0. |
| SC | 27 (3,309/9,035) | 1.10 (0.86–1.40) | 0.060/37.6 | 0.98 (0.88–1.09) | 0.624/0.0 | 1.06 (0.90–1.24) | 0.159/25.7 | 1.05 (0. |
| SC/Asian | 9 (692/1,921) | 1.02 (0.58–1.78) | 0.127/47.3 | 0.96 (0.75–1.24) | 0.899/0.0 | 1.03 (0.59–1.78) | 0.138/45.6 | 1.30 (0. |
| SC/Caucasian | 15 (2,333/6,206) | 1.13 (0.85–1.50) | 0.037/48.2 | 1.00 (0.89–1.12) | 0.323/12.7 | 1.06 (0.89–1.26) | 0.116/35.3 | 0.99 (0. |
| AC | 30 (3,745/9,598) | 1.07 (0.91–1.25) | 0.472/0.0 | 0.95 (0.86–1.04) | 0.641/0.0 | 1.03 (0.88–1.22) | 0.514/0.0 | 0.97 (0. |
| Smoking | | | | | | | | |
| Yes | 23 (5,858/5,287) | **1.33 (1.08–1.64)** | 0.688/0.0 | 1.03 (0.91–1.17) | 0.330/13.2 | **1.29 (1.01–1.57)** | 0.732/0.0 | 1.05 (0. |
| No | 23 (1,543/3,027) | 1.06 (0.72–1.56) | 0.676/0.0 | 1.12 (0.91–1.39) | 0.673/0.0 | 1.04 (0.72–1.50) | 0.641/0.0 | 1.02 (0. |
| Gender | | | | | | | | |
| Male | 17 (4,863/5,175) | 1.09 (0.94–1.27) | 0.203/23.2 | 1.01 (0.92–1.10) | 0.300/14.0 | 1.08 (0.94–1.25) | 0.316/12.5 | 1.03 (0. |
| Female | 13 (2,812/3,101) | 1.01 (0.76–1.34) | 0.049/47.0 | 0.92 (0.82–1.04) | 0.499/0.0 | 1.04 (0.79–1.36) | 0.042/48.4 | 0.95 (0. |

HB = hospital-based studies, PB = population-based studies, SCLC = small-cell lung cancer, LCLC = large cell lung carcinoma, SC = squamous carcinoma, AC = adenocarcinoma

**Supplemental Table 16** Meta-analysis of the combined effects of *GSTM1* present/null and *GSTT1* present/null on lung cancer risk

| Variable | N | Model 1 | | Model 2 | | Model 3 | | Model 4 | | M |
|---|---|---|---|---|---|---|---|---|---|---|
| | (Case/Control) | OR (95% CI) | $P_h$/$I^2$ | OR (95% CI) | $P_h$/$I^2$ | OR (95% CI) | $P_h$/$I^2$ | OR (95% CI) | $P_h$/$I^2$ | C |
| Overall | 45 | **1.34** | 0.001/ | **1.27** | <0.001/ | **1.53** | <0.001/ | **1.20** | <0.001/ | **1** |
| | (15,560/15,914) | **(1.11–1.61)** | 54.7 | **(1.11–1.46)** | 57.0 | **(1.30–1.80)** | 61.6 | **(1.08–1.33)** | 51.5 | ( |
| Ethnicity | | | | | | | | | | |
| Caucasian | 20 | 1.36 | <0.001/ | 1.12 | 0.005/ | **1.14** | 0.220/ | 1.12 | 0.020/ | **1** |
| | (8,618/10,118) | (0.98–-1.87) | 66.7 | (0.94–1.35) | 61.9 | **(1.02–1.28)** | 20.5 | (0.99–1.26) | 46.9 | ( |
| Asian | 15 (5,813/4,339) | **1.40** | 0.041/47.1 | **1.52** | 0.036/48.2 | **1.99** | <0.001/ | **1.40** | 0.008/ | **1** |
| | | **(1.06–1.84)** | | **(1.17–1.98)** | | **(1.40–2.85)** | 75.6 | **(1.10–1.79)** | 56.8 | ( |
| Indian | 3 (632/632) | 1.52 | 0.706/0.0 | **1.53** | 0.415/0.0 | **2.53** | 0.473/0.0 | **1.49** | 0.674/0.0 | **1** |
| | | (0.93–2.48) | | **(1.13–2.07)** | | **(1.61–3.98)** | | **(1.18–1.88)** | | ( |
| African | 3 (219/278) | – | – | – | – | 0.56 | 0.800/0.0 | 0.38 | 0.636/0.0 | 0 |
| | | | | | | (0.20–1.62) | | (0.13–1.14) | | (( |
| Source of control | | | | | | | | | | |
| HB | 31 (5,581/6,341) | **1.30** | 0.034/42.2 | **1.36** | 0.014/48.2 | **1.57** | 0.011/45.1 | **1.24** | 0.003/50.3 | **1** |
| | | **(1.01–1.68)** | | **(1.12–1.66)** | | **(1.27–1.94)** | | **(1.06–1.45)** | | ( |
| PB | 12 (7,852/6,542) | **1.73** | 0.003/75.1 | 1.21 | 0.001/79.0 | **1.54** | <0.001/76.1 | 1.17 | 0.009/59.0 | **1** |
| | | **(1.13–2.65)** | | (0.87–1.70) | | **(1.12–2.13)** | | (0.98–1.40) | | ( |
| Matching | | | | | | | | | | |
| Yes | 17 (3,341/4,069) | 1.34 | 0.004/74.1 | 1.54 | <0.001/82. | **1.43** | 0.023/51.7 | 1.30 | <0.001/68. | **1** |
| | | (0.78–2.31) | | (0.96–2.46) | 9 | **(1.04–1.97)** | | (0.98–1.73) | 9 | ( |
| No | 28 | **1.34** | 0.009/49.0 | **1.21** | 0.037/40.1 | **1.57** | <0.001/66.0 | **1.16** | 0.028/38.9 | **1** |
| | (12,319/11,845) | **(1.10–1.64)** | | **(1.06–1.38)** | | **(1.29–1.91)** | | **(1.05–1.28)** | | ( |
| Quality score | | | | | | | | | | |
| > 12 | 20 | 1.26 | 0.003/66.3 | **1.24** | <0.001/78. | **1.50** | <0.001/71.8 | **1.19** | <0.001/69. | **1** |
| | (10,000/9,217) | (0.99–1.61) | | **(1.01–1.55)** | 4 | **(1.17–1.92)** | | **(1.00–1.41)** | 0 | ( |
| ⩽12 | 25 (5,660/6,697) | **1.39** | 0.072/37.4 | **1.28** | 0.510/0.0 | **1.56** | 0.012/47.5 | **1.17** | 0.333/10.0 | **1** |
| | | **(1.06–1.84)** | | **(1.10–1.49)** | | **(1.24–1.96)** | | **(1.07–1.28)** | | ( |
| Sample size | | | | | | | | | | |
| > 200 | 36 | **1.37** | <0.001/64. | **1.25** | <0.001/65. | **1.53** | <0.001/66.5 | **1.19** | <0.001/58. | **1** |
| | (15,173/15,284) | **(1.11–1.69)** | 1 | **(1.08–1.46)** | 7 | **(1.29–1.83)** | | **(1.07–1.33)** | 6 | ( |
| ⩽ 200 | 9 (487/630) | 1.19 | 0.646/0.0 | **1.49** | 0.873/0.0 | **1.48** | 0.288/18.6 | 1.33 | 0.685/0.0 | **1** |
| | | (0.77–1.84) | | **(1.02–2.20)** | | **(1.01–2.17)** | | (0.94–1.87) | | ( |

Model 1 = M1 present/T1 null vs. M1 present/T1 present, Model 2 = M1 null/T1 present vs. M1 present/T1 present, Model 3 = M1 null/T1 null vs. M1 present/T1 present, Model 4 = All one risk genotypes vs. M1 present/T1 present, Model 5 = All risk genotypes vs. M1 present/T1 present, Model 6 = M1 null/T1 null vs. M1 present/T1 present + M1 present/T1 null + M1 null/T1 present, HB = hospital-based studies, PB = population-based studies

**Supplemental Table 17** Meta-analysis of the combined effects of *GSTM1* present/null and *GSTP1* IIe105Val on lung cancer risk

| Variable | Sample size | Model 1 | | Model 2 | | Model 3 | | Model 4 | | Mo[illegible] |
|---|---|---|---|---|---|---|---|---|---|---|
| | | OR (95% CI) | $P_h/I^2$ | OR (95% CI) | $P_h/I^2$ | OR (95% CI) | $P_h/I^2$ | OR (95% CI) | $P_h/I^2$ | OR [illegible] |
| Overall | 21 (4,538/5,604) | **1.15 (1.01–1.31)** | 0.186/24.9 | 1.02 (0.86–1.22) | 0.086/36.2 | 1.12 (0.98–1.29) | 0.012/45.9 | **1.31 (1.09–1.56)** | 0.010/46.8 | **1.1[illegible] (1.0[illegible]** |
| Ethnicity | | | | | | | | | | |
| Caucasian | 13 (3,384/4,246) | 1.06 (0.90–1.24) | 0.810/0.0 | 0.98 (0.84–1.15) | 0.411/2.5 | 1.04 (0.93–1.17) | 0.347/9.8 | **1.21 (1.00–1.47)** | 0.049/41.2 | 1.1[illegible] (0.9[illegible] |
| Asian | 2 (209/316) | **1.68 (1.08–2.60)** | 0.937/0.0 | 1.29 (0.72–2.31) | 0.229/30.8 | **1.56 (1.03–2.35)** | 0.597/0.0 | **2.54 (1.50–4.33)** | 0.695/0.0 | **1.7[illegible] (1.1[illegible]** |
| Indian | 3 (632/632) | **1.44 (1.09–1.90)** | 0.142/48.8 | 1.08 (0.54–2.20) | 0.008/79.1 | 1.17 (0.66–2.05) | 0.005/81.0 | 1.34 (0.91–1.98) | 0.212/35.6 | 1.2[illegible] (0.7[illegible] |
| African | 2 (113/146) | – | – | – | – | **1.99 (1.00–3.94)** | 0.170/46.8 | 1.96 (0.88–4.40) | 0.368/0.0 | **1.9[illegible] (1.0[illegible]** |
| Source of control | | | | | | | | | | |
| HB | 12 (3,323/3,720) | 1.14 (0.92–1.42) | 0.054/47.7 | 0.98 (0.79–1.22) | 0.092/41.3 | 1.06 (0.88–1.29) | 0.003/60.4 | 1.17 (0.94–1.46) | 0.031/48.3 | 1.1[illegible] |
| PB | 5 (528/745) | 1.78 (0.79–4.03) | – | 1.79 (0.82–3.92) | – | **1.43 (1.05–1.94)** | 0.334/12.5 | **1.46 (1.04–2.05)** | 0.428/0.0 | **1.4[illegible] (1.0[illegible]** |
| Matching | | | | | | | | | | |
| Yes | 13 (2,456/3,029) | **1.34 (1.12–1.61)** | 0.140/37.9 | 1.18 (0.96–1.45) | 0.219/27.4 | **1.32 (1.09–1.61)** | 0.032/46.7 | **1.55 (1.17–2.06)** | 0.006/56.5 | **1.3[illegible] (1.1[illegible]** |
| No | 8 (2,082/2,575) | 0.99 (0.83–1.18) | 0.890/0.0 | 0.88 (0.74–1.05) | 0.272/20.7 | 0.93 (0.80–1.07) | 0.708/0.0 | 1.05 (0.89–1.24) | 0.505/0.0 | 0.9[illegible] |
| Quality score | | | | | | | | | | |
| > 12 | 12 (3,053/3,606) | **1.32 (1.01–1.71)** | 0.056/53.6 | 1.03 (0.86–1.23) | 0.208/30.3 | **1.26 (1.05–1.52)** | 0.034/47.6 | **1.31 (1.02–1.68)** | 0.017/52.5 | **1.2[illegible] (1.0[illegible]** |
| ≤12 | 9 (1,485/1,998) | 1.03 (0.84–1.27) | 0.680/0.0 | 0.96 (0.73–1.27) | 0.074/45.8 | 0.96 (0.81–1.13) | 0.173/30.7 | **1.30 (1.07–1.58)** | 0.107/39.1 | 1.0[illegible] (0.8[illegible] |
| HWE | | | | | | | | | | |
| Yes | 19 (4,117/5,183) | 1.08 (0.94–1.24) | 0.516/0.0 | 1.00 (0.87–1.15) | 0.397/5.0 | 1.08 (0.97–1.20) | 0.136/26.9 | **1.34 (1.10–1.62)** | 0.006/51.0 | **1.1[illegible] (1.0[illegible]** |
| No | 2 (421/421) | 1.48 (0.84–2.61) | 0.137/54.7 | 1.00 (0.28–3.58) | 0.003/88.6 | 1.13 (0.41–3.13) | 0.001/90.3 | 1.09 (0.68–1.74) | 0.398/0.0 | 1.1[illegible] (0.4[illegible] |

Model 1 = *M1* null/*P1* IIe/IIe vs. *M1* present/*P1* IIe/IIe, Model 2 = *M1* present/P1 Val* vs. *M1* present/*P1* IIe/IIe, Model 3 = (*M1* null/*P1* IIe/IIe + *M1* present/*P1* Val*) vs. *M1* present/*P1* IIe/IIe; Model 4 = *M1* null/*P1* Val* vs. *M1* present/ *P1* IIe/IIe, Model 5 = All risk genotypes vs. *M1* present/*P1* IIe/IIe, Model 6 = *M1* null/*P1* Val* vs. (*M1* present/*P1* IIe/IIe + *M1* null/*P1* IIe/IIe + *M1* Present/*P1* Val*), HB = hospital-based studies, PB = population-based studies

**Supplemental Table 18** Meta-analysis of the combined effects of *GSTT1* present/null and *GSTP1* Ile105Val on lung cancer risk

| Variable | Sample size | Model 1 | | Model 2 | | Model 3 | | Model 4 | | [illegible] |
|---|---|---|---|---|---|---|---|---|---|---|
| | | OR (95% CI) | $P_h/I^2$ | OR (95% CI) | $P_h/I^2$ | OR (95% CI) | $P_h/I^2$ | OR (95% CI) | $P_h/I^2$ | [illegible] |
| Overall | 17 | **1.32** | 0.600/0.0 | 0.96 | 0.149/31.3 | 1.03 | 0.162/25.4 | **1.55** | 0.005/53.7 | [illegible] |
| | (3,507/4,151) | **(1.10–1.58)** | | (0.85–1.08) | | (0.93–1.14) | | **(1.18–2.02)** | | [illegible] |
| Ethnicity | | | | | | | | | | |
| Caucasian | 9 (2,356/2,794) | 1.16 | 0.632/0.0 | 0.93 | 0.228/29.1 | 0.97 | 0.321/13.6 | **1.42** | 0.042/50.2 | [illegible] |
| | | (0.90–1.49) | | (0.80–1.05) | | (0.86–1.09) | | **(1.03–1.95)** | | [illegible] |
| Asian | 2 (209/316) | 1.33 | 0.823/0.0 | 1.04 | 0.036/77.3 | 1.27 | 0.473/0.0 | **2.29** | 0.327/0.0 | [illegible] |
| | | (0.86–2.05) | | (0.31–3.58) | | (0.85–1.89) | | **(1.33–3.93)** | | [illegible] |
| Indian | 3 (632/632) | **1.75** | 0.284/20.5 | 1.01 | 0.154/46.5 | 1.18 | 0.101/56.4 | 2.06 | 0.016/75.8 | [illegible] |
| | | **(1.21–2.55)** | | (0.77–1.34) | | (0.93–1.51) | | (0.75–5.64) | | [illegible] |
| African | 2 (110/145) | – | – | – | – | 1.20 | 0.109/61.1 | 1.28 | 0.303/5.9 | [illegible] |
| | | | | | | (0.36–3.99) | | (0.53–3.06) | | [illegible] |
| Source of control | | | | | | | | | | |
| HB | 9 (2,278/2,476) | **1.32** | 0.319/14.5 | 0.96 | 0.172/33.5 | 1.01 | 0.236/23.3 | **1.54** | 0.002/67.9 | [illegible] |
| | | **(1.06–1.64)** | | (0.82–1.12) | | (0.89–1.15) | | **(1.01–2.37)** | | [illegible] |
| PB | 5 (675/833) | 1.25 | – | 0.78 | – | 1.02 | 0.162/38.9 | **1.70** | 0.244/26.7 | [illegible] |
| | | (0.48–3.29) | | (0.54–1.12) | | (0.81–1.28) | | **(1.16–2.49)** | | [illegible] |
| Matching | | | | | | | | | | |
| Yes | 10 | 1.22 | 0.676/0.0 | 1.12 | 0.074/53.2 | 1.10 | 0.294/16.2 | **1.41** | 0.088/40.5 | [illegible] |
| | (2,084/2,407) | (0.97–1.54) | | (0.81–1.56) | | (0.96–1.25) | | **(1.02–1.95)** | | [illegible] |
| No | 7 (1,423/1,744) | **1.50** | 0.426/0.0 | 0.90 | 0.434/0.0 | 0.95 | 0.195/30.5 | **1.71** | 0.012/63.4 | [illegible] |
| | | **(1.11–2.01)** | | (0.76–1.06) | | (0.82–1.10) | | **(1.09–2.67)** | | [illegible] |
| Quality score | | | | | | | | | | |
| > 12 | 11 | 1.21 | 0.677/0.0 | 0.94 | 0.133/43.2 | 1.01 | 0.186/27.1 | **1.52** | 0.029/50.1 | [illegible] |
| | (2,439/2,784) | (0.95–1.55) | | (0.80–1.11) | | (0.90–1.14) | | **(1.09–2.12)** | | [illegible] |
| ⩽12 | 6 (1,068/1,367) | **1.47** | 0.413/0.5 | 0.98 | 0.191/32.7 | 1.06 | 0.186/33.4 | 1.59 | 0.020/62.6 | [illegible] |
| | | **(1.11–1.94)** | | (0.81–1.18) | | (0.89–1.26) | | (0.98–2.56) | | [illegible] |
| HWE | | | | | | | | | | |
| Yes | 15 | **1.29** | 0.473/0.0 | 0.96 | 0.162/32.0 | 1.03 | 0.175/25.3 | **1.58** | 0.004/56.5 | [illegible] |
| | (3,086/3,730) | **(1.06–1.58)** | | (0.85–1.10) | | (0.93–1.14) | | **(1.18–2.10)** | | [illegible] |
| No | 2 (421/421) | 1.45 | 0.474/0.0 | 0.90 | 0.102/62.6 | 1.01 | 0.102/62.6 | 1.35 | 0.134/55.5 | [illegible] |
| | | (0.91–2.29) | | (0.63–1.28) | | (0.61–1.67) | | (0.50–3.65) | | [illegible] |

Model 1 = *T1* null/*P1* Ile/Ile vs. *T1* present/*P1* Ile/Ile, Model 2 = T1 present/P1 Val* vs. *T1* present/*P1* Ile/Ile, Model 3 = (*T1* null/*P1* Ile/Ile + *T1* present/*P1* Val*) vs. *T1* present/*P1* Ile/Ile, Model 4 = *T1 n*ull/*P1* Val* vs. *T1* present/*P1* Ile/Ile, Model 5 = All risk genotypes vs. *T1* present/*P1* Ile/Ile, Model 6 = T*1* null/*P1* Val* vs. (T*1* present/*P1* Ile/Ile + T*1* null/*P1* Ile/Ile + T*1* Present/*P1* Val*), HB = hospital-based studies, PB = population-based studies

**Supplemental Table 19** Meta-analysis of the combined effects of *GSTM1* present/null, *GSTT1* present/null and *GSTP1* present/null on lung cancer risk

| Variable | Sample size | Model 1 | | Model 2 | | Model 3 | | Model 4 | | Model 5 | | Model 6 | | Model 7 |
|---|---|---|---|---|---|---|---|---|---|---|---|---|---|---|
| | | OR (95% CI) | $P_h/I^2$ | OR (95% CI) | $P_h/I^2$ | OR (95% CI) | $P_h/I^2$ | OR (95% CI) | $P_h/I^2$ | OR (95% CI) | $P_h/I^2$ | OR (95% CI) | $P_h/I^2$ | OR (95% CI) |
| Overall | 7 (436/672) | 1.08 (0.61–1.90) | – | 1.44 (0.66–3.13) | – | 1.01 (0.55–1.86) | – | 1.16 (0.95–1.41) | 0.932/0.0 | 1.65 (0.65–4.18) | – | 1.65 (0.65–4.18) | – | **2.81 (1.02–7.79)** |
| HWE | | | | | | | | | | | | | | |
| Yes | 6 (345/541) | – | – | – | – | – | – | – | – | – | – | – | – | – |

Model 1 = M1 null/T1 present/P1 IIe/IIe vs. M1 present/T1 present/P1 IIe/IIe, Model 2 = M1 present/T1 null/P1 IIe/IIe vs. M1 present/T1 present/P1 IIe/IIe, Model 3 = M1 present/T1 present/P1 Val 1 vs. M1 present/T1 present/P1 IIe/IIe, Model 4 = all one high-risk genotype vs. vs. M1 present/T1 present/P1 IIe/IIe, Model 5 = M1 null/T1 null/P1 IIe/IIe vs. M1 present/T1 present/P1 IIe/IIe, Model 6 = M1 null/T1 present/P1 Val 1 vs. M1 present/T1 present/P1 IIe/IIe, Model 7 = M1 present/T1 null/P1 Val1 vs. M1 present/T1 present/P1 IIe/IIe, Model 8 = all two high-risk genotype vs. M1 present/T1 present/P1 IIe/IIe, Model 9 = M1 null/T1 null/P1 Val 1 vs. M1 present/T1 present/P1 IIe/IIe, Model 10 = M1 null/T1 null/P1 Val 1 vs. M1 present/T1 present/P1 IIe/IIe + all one high-risk genotype + all two high-risk genotypes
