# Supplementary material for: Individual and combined effects of GSTM1, GSTT1, and GSTP1 polymorphisms on lung cancer risk: A meta-analysis and re-analysis of systematic meta-analyses
Source: Medicine (Baltimore). 2021 Jul 2;100(26):e26104. doi: 10.1097/MD.0000000000026104 (PMC8257913; doi:10.1097/MD.0000000000026104)
Supplement: Supplemental Digital Content [file medi-100-e26104-s002.pdf]

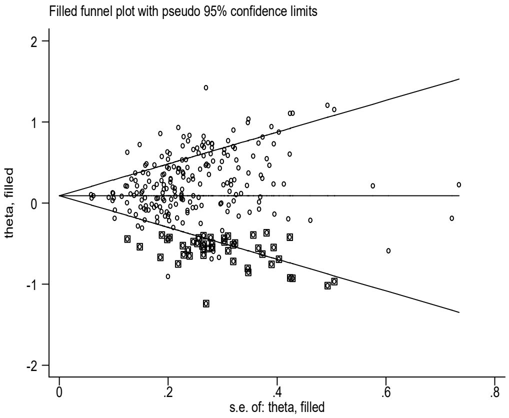


251658240

**Supplemental Fig. 1** The Duval and Tweedie nonparametric "trim and fill" method's funnel plot of the *GSTM1* present/null polymorphism

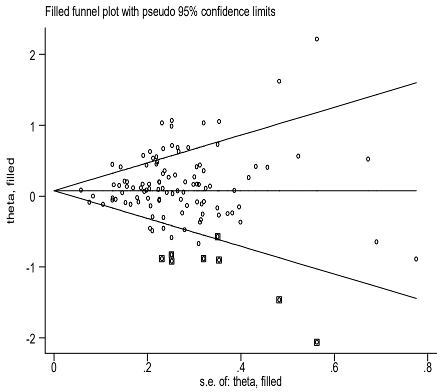


251658240

**Supplemental Fig. 2** The Duval and Tweedie nonparametric "trim and fill" method's funnel plot of the *GSTT1* present/null polymorphism

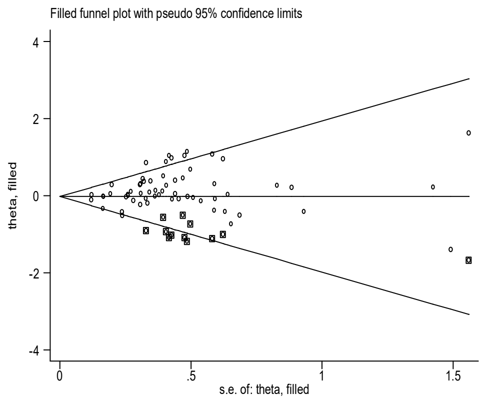


251658240

**Supplemental Fig. 3** The Duval and Tweedie nonparametric “trim and fill” method’s funnel plot of the *GSTP1* IIe105Val polymorphism (Val/Val vs. IIe/IIe)

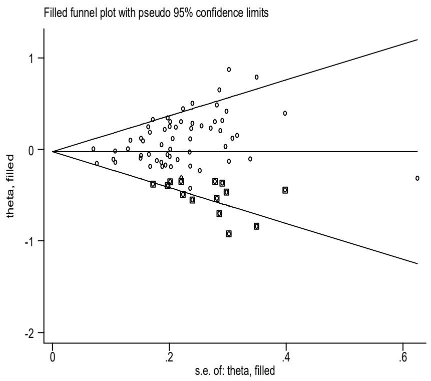


251658240

**Supplemental Fig. 4** The Duval and Tweedie nonparametric “trim and fill” method’s funnel plot of the *GSTP1* IIe105Val polymorphism (IIe/Val vs. IIe/IIe)

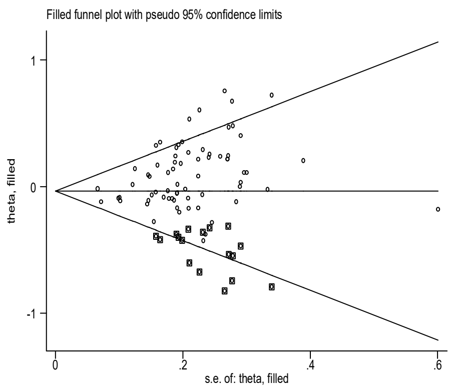


251658240

**Supplemental Fig. 5** The Duval and Tweedie nonparametric “trim and fill” method’s funnel plot of the *GSTP1* IIe105Val polymorphism (Val/Val + IIe/Val vs. IIe/IIe)

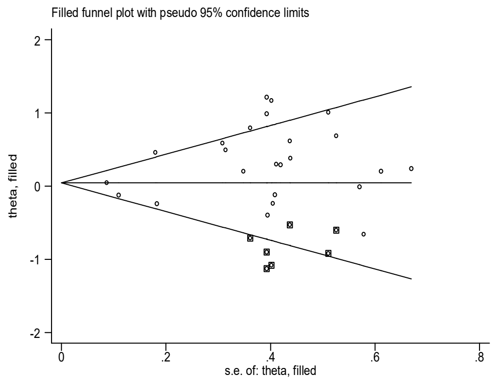


251658240

**Supplemental Fig. 6** The Duval and Tweedie nonparametric "trim and fill" method's funnel plot of the combined effects of *GSTM1* and *GSTT1* present/null polymorphisms (model 1)

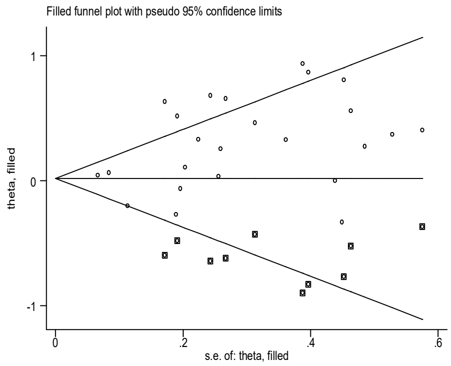


251658240

**Supplemental Fig. 7** The Duval and Tweedie nonparametric "trim and fill" method's funnel plot of the combined effects of *GSTM1* and *GSTT1* present/null polymorphisms (model 2)

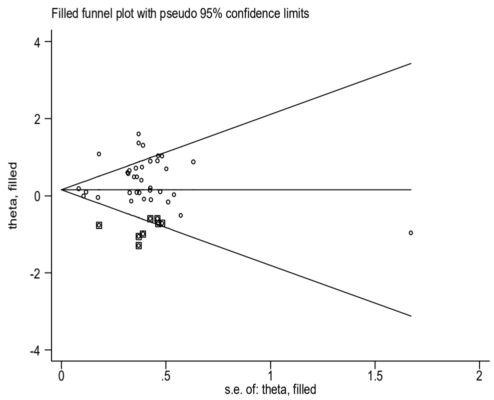


251658240

**Supplemental Fig. 8** The Duval and Tweedie nonparametric "trim and fill" method's funnel plot of the combined effects of *GSTM1* and *GSTT1* present/null polymorphisms (model 3)

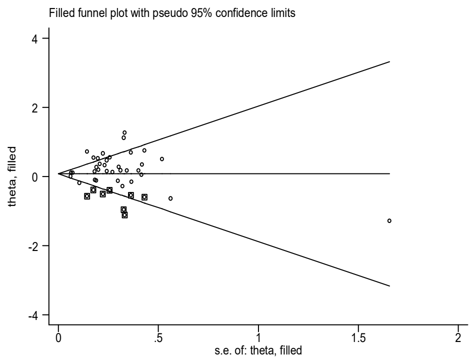

251658240

**Supplemental Fig. 9** The Duval and Tweedie nonparametric “trim and fill” method’s funnel plot of the combined effects of *GSTM1* and *GSTT1* present/null polymorphisms (model 5)

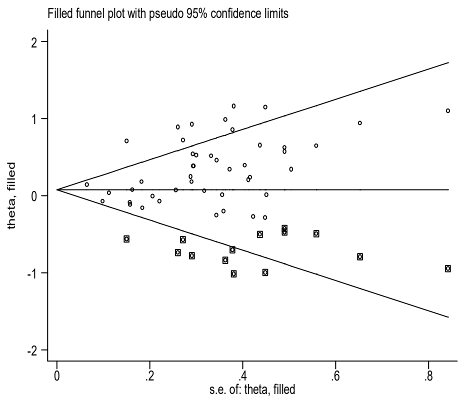


251658240

**Supplemental Fig. 10** The Duval and Tweedie nonparametric “trim and fill” method’s funnel plot of the combined effects of *GSTM1* and *GSTT1* present/null polymorphisms (model 6)

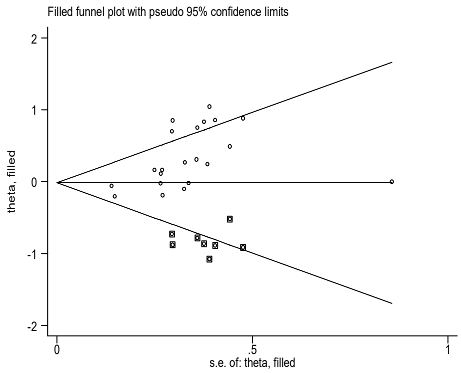


251658240

**Supplemental Fig. 11** The Duval and Tweedie nonparametric “trim and fill” method’s funnel plot of the combined effects of *GSTM1* and *GSTP1* IIe105Val polymorphisms (model 4)

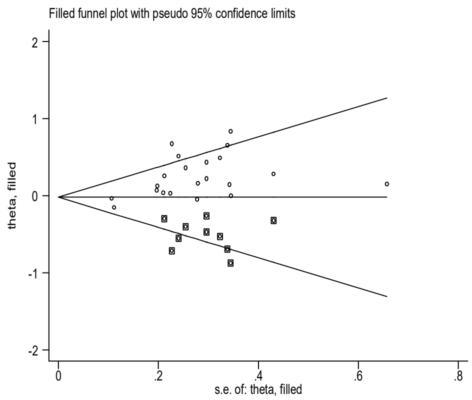


251658240

**Supplemental Fig. 12** The Duval and Tweedie nonparametric “trim and fill” method’s funnel plot of the combined effects of *GSTM1* and *GSTP1* IIe105Val polymorphisms (model 6)
